# Synthesis of non-racemic 4-nitro-2-sulfonylbutan-1-ones via Ni(II)-catalyzed asymmetric Michael reaction of β-ketosulfones

**DOI:** 10.3762/bjoc.15.127

**Published:** 2019-06-12

**Authors:** Alexander N Reznikov, Anastasiya E Sibiryakova, Marat R Baimuratov, Eugene V Golovin, Victor B Rybakov, Yuri N Klimochkin

**Affiliations:** 1 Department of Organic Chemistry, Samara State Technical University, Molodogvardeyskaya str., 244, 443100 Samara, Russian Federation; 2 Department of Chemistry, Moscow State University, Leninskie Gory, 1, 119991, Mosсow, Russian Federation

**Keywords:** asymmetric catalysis, chiral diamine ligands, ketosulfones, Michael addition, nickel complexes, nitroalkenes

## Abstract

Functionally substituted sulfones with stereogenic centers are valuable reagents in organic synthesis and key motifs in some bioactive compounds. The asymmetric Michael addition of β-ketosulfones to conjugated nitroalkenes in the presence of Ni(II) complexes with various chiral vicinal diamines was studied. This reaction provides convenient access to non-racemic 4-nitro-2-sulfonylbutan-1-ones with two stereocenters with high yield and excellent enantioselectivity (up to 99%). It has been established that the catalytic Michael reaction itself was carried out with high diastereoselectivity, but the Michael adducts may epimerize at the C-2 position at a significant rate. Conditions for the preparation of individual diastereomers were found.

## Introduction

**Figure 1:**
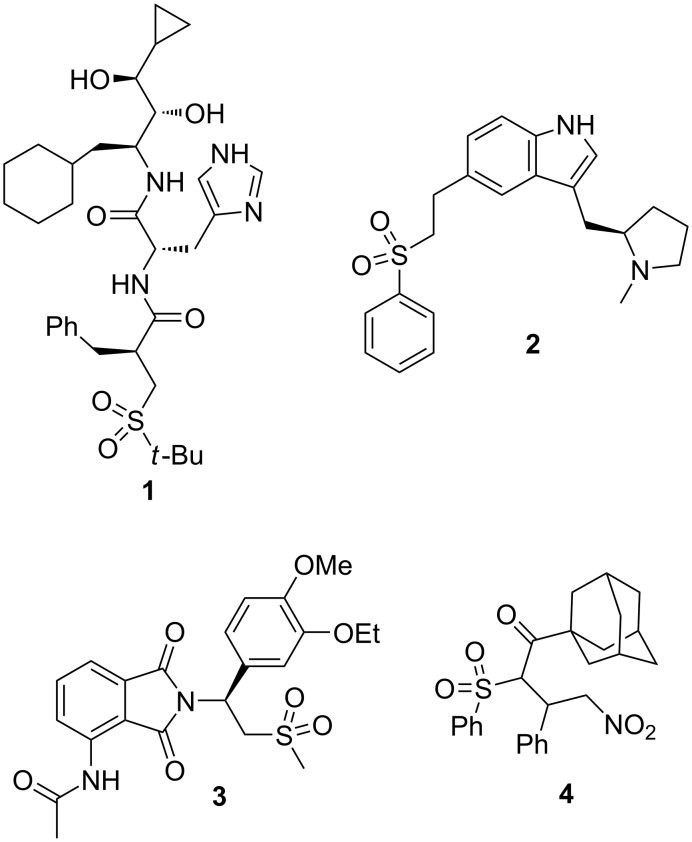
Рharmacologically active sulfones.

Sulfones are widely used in organic synthesis, particularly, in various reactions of C–C and C=C-bond formation [1–4]. The use of sulfones in Julia–Kocienski [1] and Ramberg–Bäcklund reactions [2] made this class of compounds frequently used in the synthesis of organic fine chemicals and natural compounds. In addition to using the sulfonyl group as an auxiliary, it is also included in some chiral bioactive molecules, such as remikiren (**1**, renin inhibitor for the treatment of hypertension) [5–6], eletriptan (**2**, Relpax^®^, serotonin 5-HT_1_ receptor agonist for the treatment of migraine) [7], and apremilast (**3**, Otezla^®^, inhibitor of the PDE4 for the treatment of certain types of psoriasis and psoriatic arthritis) [8] (Figure 1). Recently we have shown that racemic sulfone **4** exhibits high antiviral activity against BVDV with low cytotoxicity [9]. However, it is of great importance to obtain all stereoisomers for the study of biological activity.

Therefore, the development of methods for the asymmetric synthesis of polyfunctional sulfones is valuable. The most notable of them are Ag- and Cu-catalyzed 1,3-dipolar cycloaddition reactions, which allows to obtain chiral cyclic sulfones with high enantioselectivity [10–12]. Also non-racemic cyclic sulfones can be obtained by the Diels–Alder reaction, catalyzed by chiral Lewis acids or organocatalysts. Rh- and Cu-catalyzed CH-insertion reactions occurring at moderate or high enantioselectivity are also known [13–18]. The studied methods for obtaining acyclic sulfones with stereogenic centers in the side chain are more limited. One of the most significant approaches to obtaining both cyclic and acyclic chiral sulfones is asymmetric hydrogenation in the presence of transition metal complexes. The preparation of hydroxy sulfones from β-ketosulfones in the presence of Ru [19–20], Ir [21] and Rh [22] complexes was described. Chiral sulfones were also obtained by hydrogenation of the C=C bond with α,β-unsaturated sulfones in the presence of Ir(I) complexes with P,N-ligands [23].

The asymmetric addition of various nucleophiles to unsaturated sulfones is also considered as an effective route to chiral sulfones. The conjugated addition of arylboronic acids to unsaturated sulfones under catalysis of Rh complexes was reported [24–26]. It was shown that arylboronic acids are attached to 1,2-disubstituted α,β-unsaturated sulfones in the presence of the Rh/(*S*,*S*)-chiraphos catalytic system. Modern methods for the synthesis of functionalized sulfones, with stereocenters in the side chain, by Michael addition are based, mainly, on the use of vinyl sulfones as Michael acceptors and aldehydes [27–32], ketones [33], α-cyano esters [34–35], β-keto esters [35], β-keto acids [36], thiomalonates [37], nitroalkanes [38], oxindoles [39] and thiols [40] as Michael donors. There are only few examples of the use of sulfones as Michael donors in asymmetric addition reactions. Thus, ketonitrosulfones were obtained with good enantiomeric excesses via asymmetric addition of α-nitrosulfones to enones in the presence of organocatalysts [41]. Asymmetric addition of β-ketosulfones to nitroalkenes was implemented using various organocatalysts [42]. The reaction of ketosulfones with nitroalkenes in the presence of organocatalysts shows high enantioselectivity, however, leads to a mixture of diastereomers.

For the catalytic activation of β-ketosulfones by metal complexes chiral Lewis acids may be considered as an alternative way to carry out the asymmetric Michael reaction with their participation. Asymmetric conjugate addition of activated methylene compounds (such as diketones, keto esters and malonates) to nitroalkenes in the presence of Mg [43], Co [44–45], Mn [45] and Ru [46] complexes was performed. The most remarkable results were obtained with Ni(II) complexes as catalyst for the Michael addition of 1,3-dicarbonyl compounds to nitroalkenes [47–54]. This reaction was used as a key stage in the synthesis of non-racemic analogues of GABA and substituted pyrrolidinones with neurotropic activity [47,51–52,54].

It should be noted that the reaction of β-keto phosphonates with nitroalkenes in the presence of Ni(II) complexes with chiral vicinal diamines was carried out not only with excellent enantioselectivity, but also diastereoselectivity [55]. Moreover, β-keto sulfoxides react with nitroalkenes under catalysis by Ni(II) complexes [56].

The above considerations lead to the use of β-ketosulfones in the Ni(II)-catalyzed reaction, since the proposed mechanism [47], that involves the formation of cyclic Ni enolate, and the high CH acidity of ketosulfones (p*K*_a_ 9.8–10.5 [4]). The formation of the key intermediate can be provided by the coordination of β-ketosulfones through the oxygen atom of the sulfonyl group with Ni. For example, sulfoxide complexes with O-coordination of the corresponding ligands are widely known [57]. Although the donor properties of sulfones are lower than those of sulfoxides.

## Results and Discussion

**Figure 2:**
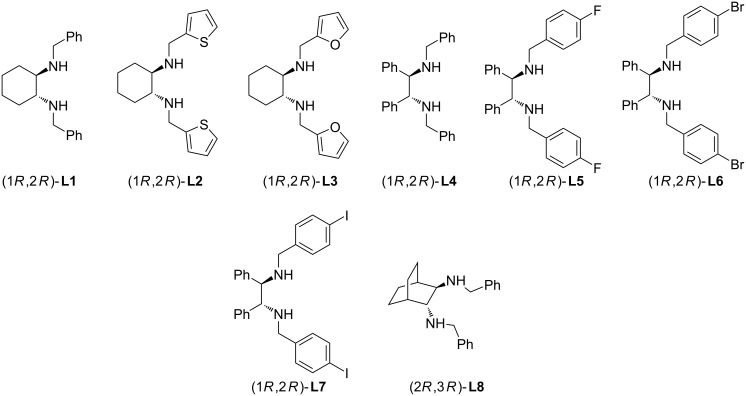
Structures of the ligands **L1**–**L8**.

Initially we carried out a screening of catalysts using the Ni(II) complexes **7a–h** with chiral vicinal diamines **L1**–**L8** (Figure 2) in a model reaction of 1-phenyl-2-(phenylsulfonyl)ethan-1-one (**5a**) with ω-nitrostyrene (**6a**). The results of the study are shown in Table 1.

**Table 1:** Screening of Ni(II) complexes with chiral diamines in the asymmetric addition of 1-phenyl-2-(phenylsulfonyl)ethan-1-one (**5**) to ω-nitrostyrene (**6a**)^a^.

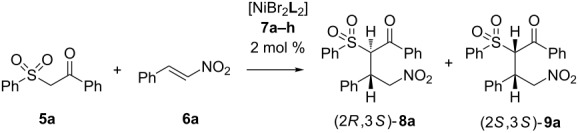				

Entry	Catalyst	Conversion, %^b^	dr^b,c^ **8a**:**9a**	ee, %^d^

**1**	[NiBr_2_**L1**_2_] **7a**	**86**	**2:1**	**91:82**
2	[NiBr_2_**L2**_2_] **7b**	82	7.3/1	89:80
3	[NiBr_2_**L3**_2_] **7c**	80	2.2:1	91:82
4	[NiBr_2_**L4**_2_] **7d**	40	6.5:1	70:65
5	[NiBr_2_**L5**_2_] **7e**	42	1.4:1	92:71
6	[NiBr_2_**L6**_2_] **7f**	40	2.4:1	96:51
7	[NiBr_2_**L7**_2_] **7g**	39	1:1.4	>99:87
8	[NiBr_2_**L8**_2_] **7h**	86	1.4:1	87:70
9	no cat.	–	–	–

^a^Reaction conditions: **5a** 1.00 mmol, **6a** 1.05 mmol, THF 1.5 mL, catalysts **7a**–**h** 0.02 mmol, 20 °C, 24 h; ^b^determined by ^1^H NMR; ^c^the absolute configuration of compound **8a** was assumed by analogy with **8d**; the relative configurations of the compounds **8a** and **9a** were assigned by studying their ^1^H NMR spectra in comparison with compound **8d**, for which X-ray diffraction data were obtained (see below); ^d^determined by chiral HPLC.

The Michael addition of the β-ketosulfone **5a** was carried out with moderate to high enantioselectivity but low diastereoselectivity and led to the formation of two diastereomers (2*R*,3*S*)-**8a** and (2*S*,3*S*)-**9a**. Since we did not succeed in growing a crystal that was suitable for X-ray determination of the absolute configuration of **8а**, we defined the absolute configuration as compared to the analogous adamantyl derivative, which will be discussed below. It should be noted that low diastereoselectivity was previously observed in the asymmetric addition of various Michael donors to nitroalkenes in the presence of both metal complexes [47] and organocatalysts [58–61]. This was explained by the authors as a result of the high CH acidity of the corresponding Michael adducts. The highest reaction rate with good enantioselectivity is achieved using catalyst **7a** (Table 1, entry 1). For this reason, further studies were carried out with catalyst **7a**. The study of the solvent effect on the reaction are summarized in Table 2.

**Table 2:** Solvent effect on the reaction of 1-phenyl-2-(phenylsulfonyl)ethan-1-one (**5a**) with ω-nitrostyrene (**6a**)^a^.

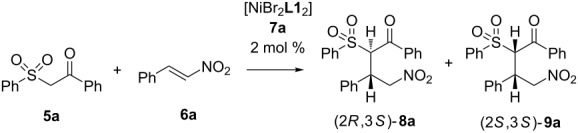				

Entry	Solvent	Conversion, %^b^	dr^b^ **8a**:**9a**	ee^c^ **8a**:**9a**

1	toluene	**98**	**1:–**	**94**
2	EtOAc	82	1.6:1	87:57
3	THF	86	2:1	91:82
4	CH_2_Cl_2_	89	2.2:1	81:63
5	CHF_2_CF_2_CH_2_OH	40	1.8:1	83:75
6	MeOH	47	1.8:1	83:35
7	CH_3_NO_2_	42	1:1	80:75
8	DMF	43	1:1	0:0
9	ethane-1,2-diol	18	1:1.9	ND^d^
10	MeCN	50	1.2:1	89:75

^a^Reaction conditions: **5a** 1.00 mmol, **6a** 1.05 mmol, solvent 1.5 mL, catalyst **7a** 0.02 mmol, 20 °C, 24 h; ^b^determined by ^1^H NMR; ^c^determined by chiral HPLC; ^d^not determined.

As can be seen from Table 2, the dr value decreases when using more polar solvents. An individual diastereomer **8a** is formed when toluene is used as a solvent (Table 2, entry 1). The highest reaction rate and enantioselectivity are also achieved in toluene. Considering these factors, toluene was chosen as the best solvent for this reaction.

**Figure 3:**
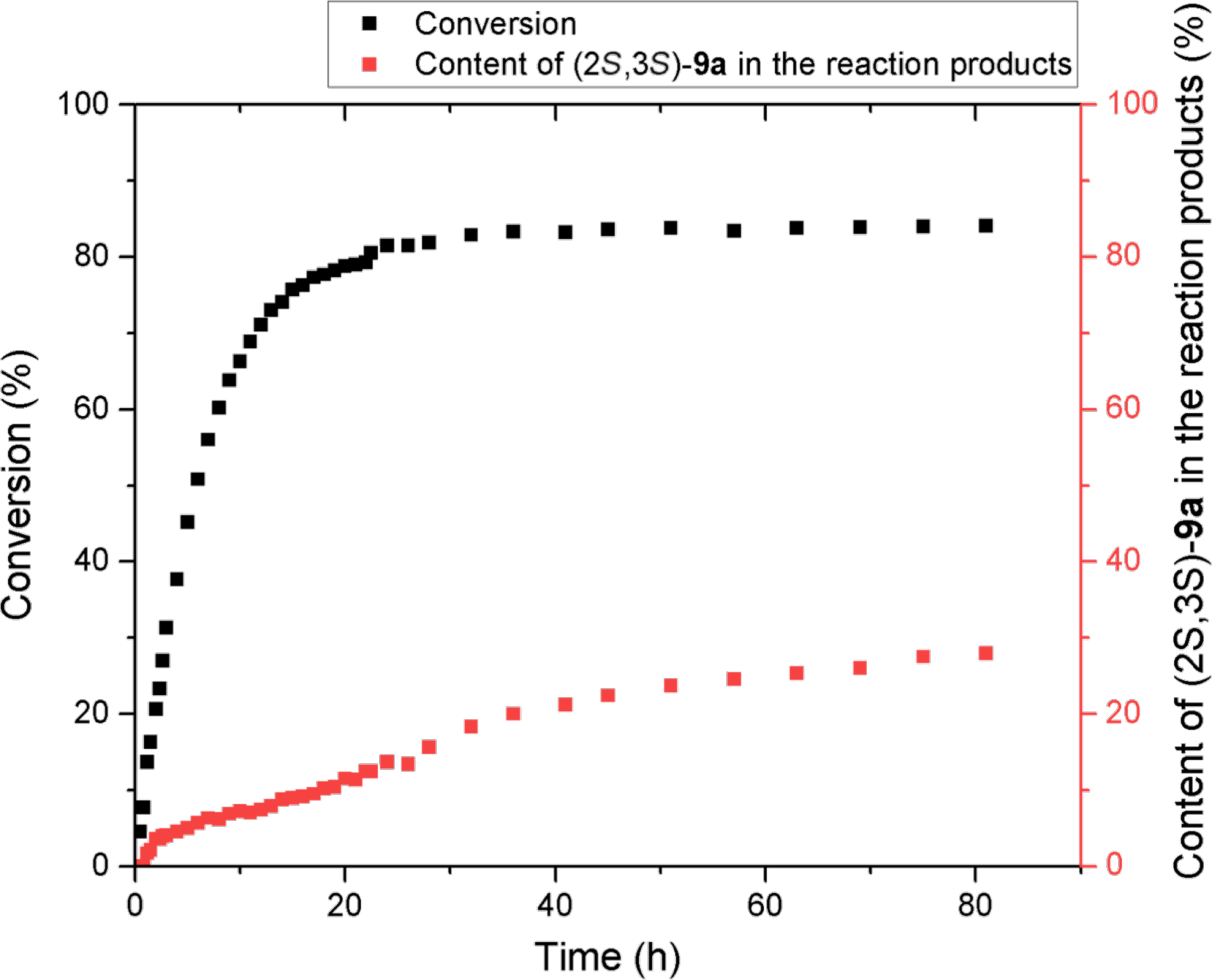
Evolution of the conversion of **5** and diastereomeric composition of the products of reaction of **5a** with **6a** in the presence of catalyst **7a** (2 mol %) in chloroform-*d*.

The obtained experimental data show that in some cases the formation of diastereomer (2*R*,3*S*)-**8a** or diastereomer (2*S*,3*S*)-**9a** as the major one is observed depending on the type of catalyst, and, on the other hand, the ratio of diastereomers in the presence of the same catalyst strongly depends on the solvent used. This suggests that the dr is determined by the rates ratio of the catalytic reaction (which can occur with high or low diastereoselectivity) and the epimerization of products **8a** or **9a**. Оne of the stereoisomers can be formed directly during the reaction. To test this hypothesis, we decided to study the evolution of dr on the course of reaction by ^1^H NMR spectroscopy (Figure 3). The reaction of sulfone **5a** with ω-nitrostyrene (**6a**) was chosen as the model reaction.

Conversion was determined by decrease of the integral intensity of the sulfone **5a** methylene group signal at 4.75 ppm. The diastereomers **8a**:**9a** ratio was determined by the ratio of the integral intensities of signals of the methine groups of **8a** and **9a** at 4.62–4.57 and 4.53–4.47 ppm, respectively. As the studies have shown, during the first 12 hours, one diastereomer (2*R*,3*S*)-**8a** was formed predominantly (dr **8a**:**9a** of more than 14:1). During this time, the conversion reached 71%. Only after this period, the appearance of the significant amount of second diastereomer (2*S*,3*S*)-**9a** was recorded. After 24 hours, the dr (**8a**:**9a**) reached 6.3:1, while the conversion is 82%. After 50 hours, the dr reached 3:1, while the reaction practically stopped.

**Figure 4:**
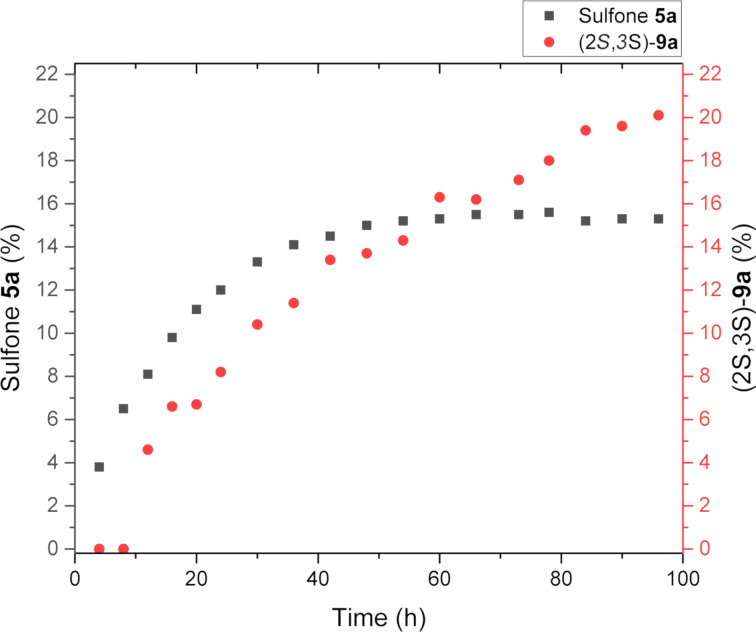
Time profile of epimerization and retro-Michael reaction of (2*R*,3*S*)-**8a** in chloroform-*d* solution.

Further, we carried out a control experiment to evaluate the epimerization rate of product **8a** in solution under the same conditions. For this purpose the individual diastereomer **8a** was dissolved in chloroform-*d* and the formation of the second diastereomer **9a** was monitored by ^1^H NMR. Surprisingly, we found that, along with the epimerization of (2*R*,3*S*)-**8a** to (2*S*,3*S*)-**9a**, retro-Michael reaction occurred (Figure 4).

It is noteworthy that firstly the formation of sulfone **5a** was more rapid than the epimerization of product **8a**. After about 60 hours the content of sulfone **5a** in solution became almost constant, while the amount of epimer **9a** continued to increase. This fact may indicate that the formation of compound **9a** may occur not only as a result of keto–enol tautomerism in solution, but also as a result of dynamic equilibrium between the Michael/retro-Michael products. This assumption is confirmed by a partial racemization of sulfones **8** at a prolonged storage, passing both the stereocenter at position 2 and the stereocenter at the position 3 (according to the HPLC data).

This study shows that the asymmetric addition of sulfone **5a** to ω-nitrostyrene (**6a**) occurs enantio- and diastereoselectively and leads to isomer **8a**, and the formation of **9a** is explained by subsequent epimerization of **8a**. The study of the reaction of various β-ketosulfones with nitroalkenes in the presence of complex **7a** was carried out under the optimized conditions (Table 3).

**Table 3:** Asymmetric addition of β-ketosulfones to nitroalkenes in the presence of complex **7a**^a^.

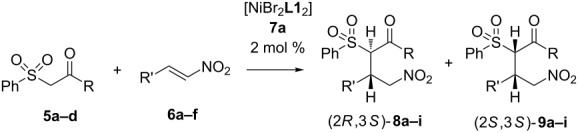									

Entry	R	R'	Compd	Conv, %^b^	dr^c^, **8**:**9**	ee^c^, % for **8**:for **9**	Yield^d^, % **8**:**9**	dr^e^, **8**:**9**	ee^e^, % for **8**: for **9**

1	Ph	Ph	**8a**/**9a**	98	1:–	94:–	77:–	1:–	>99:–
2	4-ClC_6_H_4_	Ph	**8b**/**9b**	90	1:–	92:–	72:–	1:–	>99:–
3	3-MeOC_6_H_4_	Ph	**8c**/**9c**	80	1.9:1	88:82	–:46	1: 29	–:>99
4	1-Ad	Ph	**8d**/**9d**	87	1:–	93:–	67:–	1:–	>99:–
5	Ph	4-FC_6_H_4_	**8e**/**9e**	88	1:–	96:–	74:–	1:–	>99:–
6	Ph	4-ClC_6_H_4_	**8f**/**9f**	93	1:–	>99:–	71	1:–	>99:–
7	Ph	2-ClC_6_H_4_	**8g**/**9g**	98	2.8:1	92:93	93^f^	2.8:1	92:93
8	Ph	4-NO_2_C_6_H_4_	**8h**/**9h**	95	1:–	76:–	65:–	1:–	>99:–
9	Ph	3-MeO C_6_H_4_	**8i**/**9i**	93	1:1.13	78:89	52^f^	1:2	>99:>99

^a^Reaction conditions: β-ketosulfone **5a**–**d** 1.00 mmol, nitroalkene **6a**–**f** 1.05 mmol, toluene 1.5 mL, catalyst **7a** 0.02 mmol, 20 °C, 48 h; ^b^determined by ^1^H NMR; ^c^dr (by ^1^H NMR) and ee (by chiral HPLC) in reaction mixture; the absolute configuration of compounds **8a–i** was assumed by analogy with **8d**; the relative configurations of the compounds **8a–i** and **9a–i** were assigned by studying their ^1^H NMR spectra in comparison with compound **8d**, for which X-ray diffraction data were obtained (see below); ^d^isolated yields; ^e^dr and ee after crystallization (for entries 1–6, 8, 9) or column chromatography (for entry 7); ^f^isolated yields for a mixtures of diastereomers.

The dr values for **8**:**9** are ranged from 1:– to 1:1.13. The formation of а mixture of diastereomers is observed in the presence of substituents in the 2- or 3-position of the aryl ring of sulfones or nitroalkenes.

**Figure 5:**
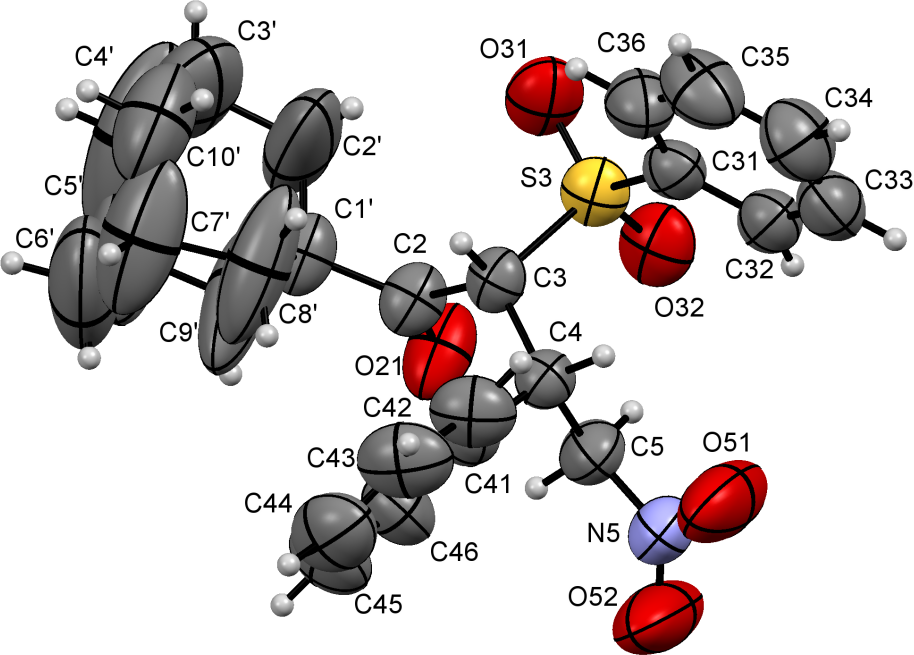
ORTEP diagram of (2*R*,3*S*)-**8d**.

Crystals suitable for X-ray analysis were obtained for compound **8d** (which is formed as an individual diastereomer). This made it possible to determine its absolute (2*R*,3*S*)-configuration, as well as to assign the absolute configuration for the other compounds obtained. The molecular structure of compound **8d** is shown in Figure 5.

The relative configurations of other Michael adducts **8** and **9** were determined by comparing their NMR data with those of compound **8d**. For (2*R*,3*S*)-isomers the value of ^3^*J*_HH_ for proton at 2-C was 5 Hz, while for (2*S*,3*S*)-isomers this value was 11 Hz.

**Scheme 1:**
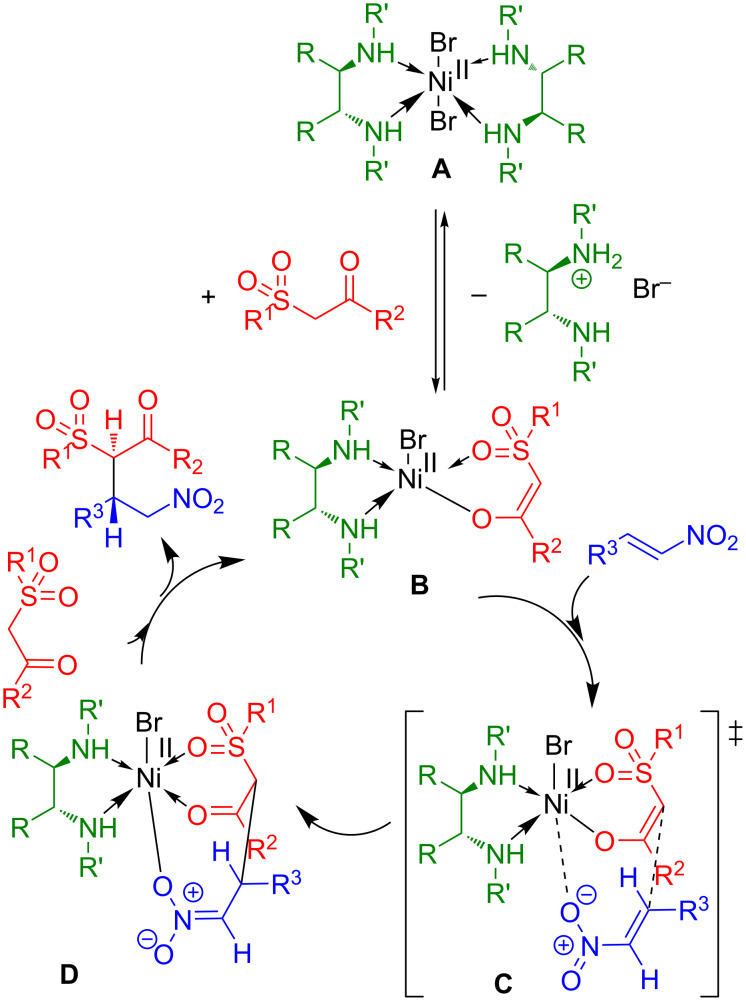
The proposed mechanism of asymmetric addition of β-ketosulfones to nitroalkenes.

It is possible to use the previously proposed mechanism for 1,3-dicarbonyl compounds [47] to explain how Ni catalysts are able to activate the substrates. The postulated catalytic cycle is summarized in Scheme 1.

**Scheme 2:**
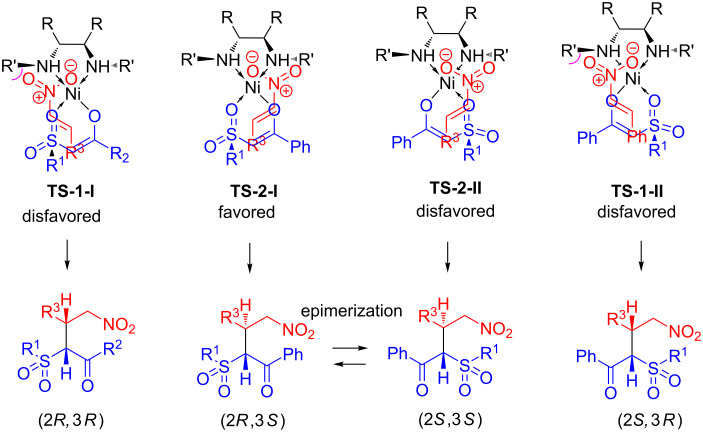
Transition state models for asymmetric addition of β-ketosulfones to nitroalkenes.

We assume that the β-ketosulfone coordinates to the Ni complex generating Ni-enolate **B**. The nitroalkene is activated by coordination to Ni. The complex **B** regenerates after the conjugate addition via transition state **C** and coordination of a new β-ketosulfone molecule to Ni. **TS1** and **TS2** are proposed by analogy with 1,3-dicarbonyl compounds [47] to rationalize the asymmetric induction. As illustrated in Scheme 2, β-ketosulfone is coordinated to Ni in more Lewis acidic equatorial position, whereas the nitroalkene is positioned in apical by avoiding the steric repulsion of benzyl groups (**TS2-I** and **2-II** vs **TS1-I** and **1-II**, Scheme 2). Additional hydrogen bonding between the hydrogen atom of the amino group and the oxygen atom of the nitroalkene in **TS2-I** and **2-II** may also help to rigidify the transition state and improve the stereoselectivities.

The observed (2*R*,3*S*)-diastereoselectivity in the presence of catalyst **7a** stems from the addition of the *Re* face of the β-ketosulfone to the *Si* face of the nitroalkene in **TS2-I**. We suppose that the CO group of sulfone is placed on the side of the nitro group, whereas the bulkier sulfonyl group is oriented opposed to the nitro group to minimize steric interactions.

## Conclusion

In summary, a convenient synthetic protocol for the preparation of valuable non-racemic 4-nitro-2-sulfonylbutan-1-ones via Ni(II)-catalyzed Michael addition was developed. Corresponding sulfones were obtained with high enantiomeric excesses (up to 99%) by asymmetric addition of β-ketosulfones to nitroalkenes in the presence of Ni(II) complexes with chiral vicinal diamines. In some cases, a high diastereoselectivity of the reaction was observed.

## Supporting Information

File 1 Experimental procedures, copies of NMR, FTIR, mass spectra, HPLC and X-ray diffraction data.
