# Supplementary material for: Synthesis of non-racemic 4-nitro-2-sulfonylbutan-1-ones via Ni(II)-catalyzed asymmetric Michael reaction of β-ketosulfones
Source: Beilstein J Org Chem. 2019 Jun 12;15:1289–97. doi: 10.3762/bjoc.15.127 (PMC6604683; doi:10.3762/bjoc.15.127)
Supplement: File 1 — Experimental procedures, copies of NMR, FTIR, mass spectra, HPLC and X-ray diffraction data. [file Beilstein_J_Org_Chem-15-1289-s001.pdf]

BEILSTEIN JOURNAL OF ORGANIC CHEMISTRY

Supporting Information

for

Synthesis of non-racemic 4-nitro-2-sulfonylbutan-1-ones via Ni(II)-catalyzed asymmetric Michael reaction of β-ketosulfones

Alexander N. Reznikov, Anastasiya E. Sibiryakova, Marat R. Baimuratov, Eugene V. Golovin, Victor B. Rybakov and Yuri N. Klimochkin

Beilstein J. Org. Chem. **2019,** *15,* 1289–1297. doi:10.3762/bjoc.15.127

Experimental procedures, copies of NMR, FTIR, mass spectra, HPLC and X-ray diffraction data

License and Terms: This is a supporting information file under the terms of the Creative Commons Attribution License (http://creativecommons.org/licenses/by/4.0). Please note that the reuse, redistribution and reproduction in particular requires that the authors and source are credited.
The license is subject to the *Beilstein Journal of Organic Chemistry* terms and conditions: (https://www.beilstein-journals.org/bjoc)

Table of Contents

Experimental procedures... S2

Copies of NMR, FTIR and mass spectra for compounds **8** and **9**.............. S15

Copies of HPLC chromatograms for compounds **8** and **9**........................... S40

X-Ray diffraction data of compound **8d**... S58

References... S61

Experimental procedures

General information

^{1}H, ^{13}C and ^{19}F NMR spectra were recorded with a JEOL JNM-ECX400 spectrometer (^{1}H NMR, 399.78 MHz, ^{13}C NMR, 100.53 MHz, ^{19}F NMR, 161.83 MHz) in $CDCl_3$ or DMSO-d_6 solution using TMS as the internal reference. FTIR spectra were recorded with a Shimadzu IRAffinity-1 spectrophotometer with Specac® Quest ATR. Melting points were measured with an OptiMelt Automated Melting Point System. High-resolution mass spectrometry was recorded with a mass-spectometer Agilent AccuTOF 6230. Optical rotations were measured on Rudolph Research Analytical (Autopol V Plus Automatic Polarimeter) with a sodium lamp at 589 nm. The enantiomeric purity of the products was determined by HPLC analysis on Waters LC equipped with a chiral stationary phase column Chiralpak AD-3 with hexane/2-propanol as eluent. Column chromatography was performed on silica gel 60, Merck (230–400 mesh). X-ray crystallographic data were collected using a Stoe STADI-VARI Pilatus-100K diffractometer.

Synthesis of initial compounds

β-Keto sulfones **5a–d** [1] and nitroalkenes **6a–f** [2] were prepared by the described procedure. Synthesis of complexes **7a, 7b–d,h** and **7e–g** were described in the work [3-5], respectively.

General procedure for the Michael addition of β-keto sulfone (5a) to ω-nitrostyrene (6a) in the presence of Ni(II) complexes

A mixture of β-keto sulfone **5a** (1.00 mmol), ω-nitrostyrene (**6a**, 1.05 mmol) and catalyst **7a–e** (0.02 mmol) in 1.5 mL of the corresponding solvent (see Tables 1,2) was stirred at

20 °C for 48 h. The reaction mixture was evaporated in vacuo, dissolved in chloroform-*d* and analyzed by NMR.

General procedure for the synthesis of compounds 8a–i/9a–i

A mixture of β-keto sulfone **5a–d** (1.00 mmol), nitroalkene **6a–f** (1.05 mmol) and catalyst **7a** (0.02 mmol) in 1.5 mL of toluene was stirred at 20 °C for 48 h. The reaction mixture was concentrated under reduced pressure and the crude product was recrystallized from toluene. In the case of compounds **8g/9g**, the crude product was purified by silica column chromatography ($CHCl_3$) to give the product as a yellow oil (mixture of diastereomers).

(2*R*,3*S*)-4-Nitro-1,3-diphenyl-2-(phenylsulfonyl)butan-1-one (**8a**)

Yield: 77 %, white crystals, m.p. 139−141 °C (toluene). $[\alpha]_D^{20}$ = +31.9 (*c* 1.0, $CHCl_3$).

^{1}H NMR (400 MHz, $CDCl_3$, 298 K) δ [ppm] 7.79−7.04 (m, 15H, Ph), 5.46 (d, 1H, H-2, $^3J_{HH}$ 5.0 Hz), 5.32 (dd,1H, H-4, $^2J_{HH}$ 14.0 Hz, $^3J_{HH}$ 3.4 Hz), 5.09 (dd, 1H, H-4, $^2J_{HH}$ 14.0 Hz, $^3J_{HH}$ 10.1 Hz), 4.57−4.52 (m, 1H, H-3); ^{13}C NMR (101 MHz, $CDCl_3$, 298 K) δ [ppm] 192.22 (C-1), 137.78, 137.18, 135.95, 134.63, 134.39, 129.45, 129.42, 129.15, 128.85, 128.80, 128.66, 127.81, 76.60 (C-4), 71.36 (C-2), 42.64 (C-3); IR (neat) ν [cm^{-1}] 3065 w, 3036 w, 2970 w, 2924 w, 1682 s, 1597 m, 1582 m, 1547 s, 1497 w, 1447 s, 1377 w, 1366 w, 1346 w, 1304 s, 1275 s, 1221 m, 1142 s, 1082 s, 991 m, 935 m, 839 w, 762 m, 746 s, 735 s, 721 s, 698 s, 683 vs, 650 m, 633 m, 611 m, 563 s, 544 m, 521 vs, 451 w, 419 m; APPI-HRMS (*m*/*z*): $[M+NH_4]^+$ calcd for $C_{22}H_{19}NO_5S\cdot NH_4^+$, 427.1322; found 427.1326. $[\alpha]_D^{20}$ = +31.9 (*c* 1.0, $CHCl_3$).

4-Nitro-1,3-diphenyl-2-(phenylsulfonyl)butan-1-one (a mixture of diastereomers (2*R*,3*S*)-**8a** and (2*S*,3*S*)-**9a**)

^{1}H NMR (400 MHz, $CDCl_3$, 298 K) δ [ppm] 7.79−7.04 (m, 15H, **8a**, 15H, **9a**, Ph), 5.61 (d, 1H, H-2, $^3J_{HH}$ 11.4 Hz, **9a**), 5.58 (dd, 1H, H-4, $^2J_{HH}$ 13.3 Hz, $^3J_{HH}$ 3.9 Hz, **9a**), 5.46 (d,

1H, H-2, $^3J_{HH}$ 5.0 Hz, **8a**), 5.32 (dd,1H, H-4, $^2J_{HH}$ 14.0 Hz, $^3J_{HH}$ 3.4 Hz, **8a**), 5.12 (dd, 1H, H-4, $^2J_{HH}$ 13.3 Hz, $^3J_{HH}$ 10.6 Hz, **9a**), 5.09 (dd, 1H, H-4, $^2J_{HH}$ 14.0 Hz, $^3J_{HH}$ 10.1 Hz, **8a**), 4.57−4.52 (m, 1H, H-3, **8a**), 4.47−4.40 (m, 1H, H-3, **9a**); ^{13}C NMR (101 MHz, $CDCl_3$, 298 K) δ [ppm] 192.23 (C-1, **8a**), 191.03 (C-1, **9a**), 137.78 (**8a**), 137.18 (**8a**), 136.62 (**9a**), 136.39 (**9a**), 135.96 (**8a**), 134.91 (**9a**), 134.63 (**8a**), 134.40 (**8a**), 133.91 (**9a**), 129.96 (**9a**), 129.46 (**8a**), 129.42 (**8a**), 129.15 (**8a**), 129.13 (**9a**), 128.85 (**8a**), 128.80 (**8a**), 128.66 (**8a**), 128.57 (**9a**), 128.25 (**9a**), 127.81 (**8a**), 78.05 (C-4, **9a**), 76.60 (C-4, **8a**), 71.36 (C-2, **8a**), 71.14 (C-2, **9a**), 43.35 (C-3, **9a**), 42.65 (C-3, **8a**).

HPLC (*n*-hexane/*i*-PrOH = 85/15, 210 nm, 1.2 mL/min) Chiralcel AD-H, t_R = 21.1 min (2*S*,3*S*), 23.2 min (2*R*,3*S*); 27.4 min (2*R*,3*R*); 44.10 min (2*S*,3*R*).

(2*R*,3*S*)-1-(4-Chlorophenyl)-4-nitro-3-phenyl-2-(phenylsulfonyl)-butan-1-one (**8b**)

Yield: 72 %, white crystals, m.p. 220−222 °C (ethanol). $[\alpha]_D^{20}$ = +26.1 (*c* 0.15, $CHCl_3$). ^{1}H NMR (400 MHz, $CDCl_3$, 298 K) δ [ppm] 7.74−7.00 (m, 14H, aromatic), 5.41 (d, 1H, H-2, $^3J_{HH}$ 5.0 Hz), 5.26 (dd, 1H, H-4, $^2J_{HH}$ 14.0 Hz, $^3J_{HH}$ 3.2 Hz), 5.06 (dd, 1H, H-4, $^2J_{HH}$ 14.0 Hz, $^3J_{HH}$ 9.8 Hz), 4.55−4.45 (m, 1H, H-3); ^{13}C NMR (101 MHz, $CDCl_3$, 298 K) δ [ppm] 191.03 (C-1), 136.63, 136.39, 134.91, 133.89, 129.96, 129.25, 129.13, 128.60, 128.57, 128.37, 128.25, 77.32 (C-4), 71.14 (C-2), 43.35 (C-3); IR (neat) ν [cm^{-1}] 3082 w, 3030 w, 2984 w, 2954 w, 1680 s, 1589 m, 1573 m, 1549 vs, 1495 w, 1450 m, 1441 m, 1402 m, 1387 m, 1365 m, 1323 w, 1304 vs, 1277 s, 1221 m, 1142 s, 1092 s, 1080 w, 993 s, 837 m, 829 m, 787 s, 762 m, 750 s, 729 s, 702 s, 681 s, 646 s, 629 s, 586 m, 561 vs, 538 m, 521 vs, 486 s, 449 w; APPI-HRMS (*m*/*z*): $[M+NH_4]^+$ calcd for $C_{22}H_{18}ClNO_5S{\cdot}NH_4^+$, 461.0932; found 461.0935. HPLC (*n*-hexane/*i*-PrOH = 70/30, 210 nm, 1.2 mL/min) Chiralcel AD-H, t_R = 9.71 min (2*R*,3*S*), 15.33 min (2*S*,3*R*).

1-(4-Chlorophenyl)-4-nitro-3-phenyl-2-(phenylsulfonyl)-butan-1-one (a mixture of diastereomers (2*R*,3*S*)-**8b** and (2*S*,3*S*)-**9b**)

^{1}H NMR (400 MHz, DMSO-d_6, 298 K) δ [ppm] 8.10−6.95 (m, 14H, **8b**, 14H, **9b**, aromatic), 6.49 (d, 1H, H-2, $^3J_{HH}$ 11.2 Hz, **9b**), 6.16 (d, 1H, H-2, $^3J_{HH}$ 9.2 Hz, **8b**), 5.72 (s), 5.70−5.63 (dd, 1H, H-4, $^2J_{HH}$ 13.0 Hz, $^3J_{HH}$ 3.6 Hz, **9b**), 5.15−5.08 (m, 1H, H-4, **9b**), 4.90−4.80 (m, 2H, C-4, **8b**), 4.35−4.25 (m, 1H, C-3, **8b**), 4.15−4.00 (m, 1H, C-3, **9b**); ^{13}C NMR (101 MHz, $CDCl_3$, 298 K) δ [ppm] 191.51 (C-1, **8b**), 190.75 (C-1, **9b**), 140.54, 140.50, 139.75, 138.92, 137.81, 136.69, 136.11, 136.01, 135.59, 135.25, 135.00, 134.60, 131.62, 130.78, 129.96, 129.64, 129.45, 129.24, 129.16, 129.07, 128.81, 128.61, 128.59, 78.87 (C-4, **8b**), 78.31 (C-4, **9b**), 69.67 (C-2, **9b**), 69.53 (C-2, **8b**), 43.96 (C-3, **8b**), 43.71 (C-3, **9b**).

(2*S*,3*S*)-1-(3-Methoxyphenyl)-4-nitro-3-phenyl-2-(phenylsulfonyl)-butan-1-one (**9c**). Yield: 46 %, white crystals, m.p. 154−155 °C (ethanol). $[\alpha]_D^{20}$ = -28.0 (*c* 1.0, $CHCl_3$). ^{1}H NMR (400 MHz, $CDCl_3$, 298 K) δ [ppm] 7.80−6.83 (m, 14H, aromatic), 5.62-5.54 (m, 1H, H-4 + 1H, H-2), 5.12 (dd, 1H, H-4, $^2J_{HH}$ 13.2 Hz, $^3J_{HH}$ 11.0 Hz), 4.47−4.41 m (1H, H-3), 3.68 (s, 3H, CH_3O); ^{13}C NMR (101 MHz, $CDCl_3$, 298 K) δ [ppm] 190.93 (C-1), 159.56, 134.88, 129.99, 129.52, 129.24, 129.14, 128.60, 128.40, 120.86, 120.52, 112.32, 78.04 (C-4), 71.44 (C-2), 55.45 (CH_3O), 43.38 (C-3); IR (neat) ν [cm^{-1}] 3091 w, 3065 w, 2976 w, 2943 w, 1682 m, 1674 m, 1599 m, 1584 m, 1549 vs, 1493 m, 1449 m, 1425 m, 1144 vs, 1082 s, 1038 m, 984 m, 924 w, 889 m, 833 m, 804 m, 789 m, 775 s, 752 s, 719 s, 700 s, 687 s, 629 w, 611 vs, 550 vs, 517 vs, 459 w, 420 m; APPI-HRMS (*m*/*z*): $[M+NH_4]^+$ calcd for $C_{23}H_{21}NO_6S\cdot NH_4^+$, 457.1428; found 457.1431.

1-(3-Methoxyphenyl)-4-nitro-3-phenyl-2-(phenylsulfonyl)-butan-1-one (a mixture of diastereomers (2*R*,3*S*)-**8c** and (2*S*,3*S*)-**9c**).

^{1}H NMR (400 MHz, $CDCl_3$, 298 K) δ [ppm] 7.80−6.83 (m, 14H, **8c**, 14H, **9c**, aromatic), 5.62-5.54 (m, 1H, H-4 + 1H, H-2, **9c**), 5.43 (d, 1H, H-2, $^3J_{HH}$ 5.0 Hz, **8c**), 5.32 (dd, 1H, H-4, $^2J_{HH}$ 14.0 Hz, $^3J_{HH}$ 3.4 Hz, **8c**), 5.12 (dd, 1H, H-4, $^2J_{HH}$ 13.2 Hz, $^3J_{HH}$ 11.0 Hz, **9c**),

5.08 (dd, 1H, H-4, $^{2}J_{HH}$ 14.0 Hz, $^{3}J_{HH}$ 10.3 Hz, **8c**), 4.57−4.52 (m, 1H, H-3, **8c**), 4.47−4.41 (m, 1H, H-3, **9c**), 3.76 (s, 3H, CH_3O, **8c**), 3.68 (s, 3H, CH_3O, **9c**).

HPLC (*n*-hexane/*i*-PrOH = 75/25, 210 nm, 1.2 mL/min) Chiralcel AD-H, t_R = 13.92 min (2*S*,3*S*), 15.76 min (2*R*,3*S*), 16.50 min (2*S*,3*R*), 30.23 min (2*R*,3*R*).

(2*R*,3*S*)-1-Adamantyl-4-nitro-3-phenyl-2-(phenylsulfonyl)butan-1-one (**8d**).

Yield: 67 %, white crystals, m.p. 163−164 °C (ethanol). $[\alpha]_D^{20}$ = +112.6 (*c* 1.0, $CHCl_3$). ^{1}H NMR (400 MHz, $CDCl_3$, 298 K) δ [ppm] 7.81−6.90 (m, 10H, Ph), 5.18 (dd, 1H, H-4, $^{2}J_{HH}$ 14.0 Hz, $^{3}J_{HH}$ 3.2 Hz), 5.02 (d, 1H, H-2, $^{3}J_{HH}$ 5.0 Hz), 4.90 (dd, 1H, H-4, $^{2}J_{HH}$ 14.0 Hz, $^{3}J_{HH}$ 10.8 Hz), 4.22−4.14 m (1H, H-3), 1.94 (broad s, 3H, Ad), 1.70−1.48 (m, 12H, Ad); ^{13}C NMR (101 MHz, $CDCl_3$, 298 K) δ [ppm] 207.69 (C-1), 138.21, 135.34, 134.64, 129.64, 129.36, 128.95, 128.81, 127.78, 75.64 (C-4), 70.58 (C-2), 42.87, 37.97, 36.09, 27.83; IR (neat) ν [cm^{-1}] 2924 m, 2900 s, 2851 m, 1692 s, 1585 w, 1553 vs, 1497 w, 1472 w, 1447 s, 1383 s, 1327 s, 1306 vs, 1217 s, 1180 w, 1150 vs, 1084 s, 1011 m, 984 m, 959 m, 934 m, 914 w, 854 m, 808 m, 771 m, 762 m, 750 m, 735 s, 698 s, 687 s, 665 s, 600 s, 561 m, 536 vs, 455 w, 442 m, 426 w; APPI-HRMS (*m*/*z*): $[M+NH_4]^+$ calcd for $C_{26}H_{29}NO_5S\cdot NH_4^+$, 485.2105; found 485.2106. HPLC (*n*-hexane/*i*-PrOH = 85/15, 210 nm, 1.2 mL/min) Chiralcel AD-H, t_R = 14.18 min (2*S*,3*R*), 16.01 min (2*R*,3*S*).

(2*R*,3*S*)-3-(4-Fluorophenyl)-4-nitro-1-phenyl-2-(phenylsulfonyl)-butan-1-one (**8e**).

Yield: 74 %, white crystals, m.p. 162−164 °C (toluene). $[\alpha]_D^{20}$ = +24.4 (*c* 1.0, $CHCl_3$). ^{1}H NMR (400 MHz, $CDCl_3$, 298 K) δ [ppm] 7.70 (d, 2H, $^{3}J_{HH}$ 7.7 Hz), 7.59 (d, 2H, $^{3}J_{HH}$ 7.7 Hz), 7.58−7.49 (m, 2H, Ph), 7.45−7.39 (m, 2H, Ph), 7.36−7.30 (m, 2H, Ph), 7.10−7.03 (m, 2H, Ph), 6.92−6.84 (m, 2H, Ph), 5.46 (d, 1H, H-2, $^{3}J_{HH}$ 5.5 Hz), 5.25 (dd, 1H, H-4, $^{2}J_{HH}$ 14.0 Hz, $^{3}J_{HH}$ 3.4 Hz), 5.03 (dd, 1H, H-4, $^{2}J_{HH}$ 14.0 Hz, $^{3}J_{HH}$ 10.1 Hz), 4.60−4.50 m (1H, H-3); ^{13}C NMR (101 MHz, $CDCl_3$, 298 K) δ [ppm] 192.06 (C-1), 162.67 (d, $^{1}J_{CF}$ 249.2 Hz), 134.68, 134.56, 129.68, 129.42, 129.11, 128.94, 128.67, 116.45 d ($^{2}J_{CF}$ 21.9

Hz), 76.82 (C-4), 71.28 (C-2), 42.02 (C-3); IR (neat) ν [cm^{-1}] 1684 s, 1597 m, 1562 m, 1547 m, 1476 w, 1449 s, 1427 m, 1379 m, 1315 m, 1308 m, 1294 s, 1275 s, 1263 s, 1206 w, 1179 w, 1140 vs, 1080 s, 999 m, 957 s, 893 m, 851 w, 797 s, 766 s, 752 vs, 723 m, 692 s, 683 vs, 665 m, 633 m, 567 m, 554 vs, 532 vs, 511 s, 455 w, 444 m, 415 w; APPI-HRMS (*m*/*z*): $[M+NH_4]^+$ calcd for $C_{22}H_{18}FNO_5S{\cdot}NH_4^+$, 445.1228; found 445.1231. HPLC (*n*-hexane/*i*-PrOH = 85/15, 210 nm, 1.2 mL/min) Chiralcel AD-H, t_R = 29.17 min (2*R*,3*S*), 35.55 min (2*S*,3*R*).

(2*R*,3*S*)-3-(4-Chlorophenyl)-4-nitro-1-phenyl-2-(phenylsulfonyl)butan-1-one (8f)

Yield: 71 %, white crystals, m.p. 138−139 °C (toluene). $[\alpha]_D^{20}$ = +25.2 (*c* 1.0, $CHCl_3$). ^1H NMR (400 MHz, $CDCl_3$, 298 K) δ [ppm] 7.69−7.01 (m, 14H, aromatic), 5.46 (d, 1H, H-2, $^3J_{HH}$ 5.5 Hz), 5.23 (dd, 1H, H-4, $^2J_{HH}$ 14.0 Hz, $^3J_{HH}$ 3.4 Hz), 5.02 (dd, 1H, H-4, $^2J_{HH}$ 14.0 Hz, $^3J_{HH}$ 9.9 Hz), 4.55−4.50 (m, 1H, H-3); ^{13}C NMR (101 MHz, $CDCl_3$, 298 K) δ [ppm] 191.92 (C-1), 137.78, 136.99, 134.84, 134.67, 134.60, 134.38, 129.61, 129.43, 129.34, 129.09, 128.97, 128.70, 76.68 (C-4), 71.12 (C-2), 42.15 (C-3). IR (neat) ν [cm^{-1}] 3074 w, 3044 w, 2980 w, 2928 w, 1682 s, 1599 m, 1584 m, 1549 vs, 1512 s, 1447 s, 1387 w, 1359 w, 1306 vs, 1273 vs, 1221 s, 1163 w, 1144 vs, 1105 w, 1082 w, 991 m, 945 w, 934 w, 895 w, 864 w, 847 m, 824 s, 797 s, 759 m, 739 s, 727 s, 683 vs, 665 m, 644 m, 571 s, 542 s, 530 vs, 500 m, 484 m, 434 m, 419 m; APPI-HRMS (*m*/*z*): $[M+NH_4]^+$ calcd for $C_{22}H_{18}ClNO_5S{\cdot}NH_4^+$, 461.0932; found 461.0923.

HPLC (*n*-hexane/*i*-PrOH = 85/15, 210 nm, 1.2 mL/min) Chiralcel AD-H, t_R = 26.76 min (2*R*,3*S*), 30.50 min (2*S*,3*S*), 32.42 min (2*S*,3*R*), 52.98 min (2*R*,3*R*).

3-(2-Chlorophenyl)-4-nitro-1-phenyl-2-(phenylsulfonyl)-butan-1-one (a mixture of diastereomers (2*R*,3*S*)-**8g** and (2*S*,3*S*)-**9g**).

Yield: 93 %, white solid, m.p. 47−49 °C. $[\alpha]_D^{20}$ = +16.1 (*c* 1.0, $CHCl_3$). ^1H NMR (400 MHz, $CDCl_3$, 298 K) δ [ppm] 7.80−6.95 (m, 14H, **8g**, 14H, **9g**, aromatic), 6.15−6.00 (m,

1H, **9g**), 5.60−5.43 (m, 2H, **8g**, 2H, **9g**), 5.37 (dd, 1H, 4-C, $^{2}J_{HH}$ 14.4 Hz, $^{3}J_{HH}$ 10.3 Hz, **8g**), 5.00−4.92 (m, 1H, **8g**); ^{13}C NMR (101 MHz, $CDCl_3$, 298 K) δ [ppm] 191.76, 190.80, 137.66, 137.21, 134.92, 134.81, 134.48, 134.14, 133.67, 132.69, 130.64, 129.97, 129.62, 129.24, 128.91, 128.68, 128.51, 127.57, 74.51 (C-2), 68.67 (C-3), 38.56 (C-2); IR (neat) ν [cm^{-1}] 3065 w, 1678 s, 1595 m, 1582 w, 1551 vs, 1477 m, 1447 s, 1379 m, 1323 s, 1310 s, 1283 s, 1180 m, 1148 vs, 1082 s, 1040 m, 999 w, 953 m, 847 w, 746 vs, 721 vs, 683 vs, 610 s, 548 s, 527 vs, 492 m, 451 m; APPI-HRMS (*m*/*z*): $[M+NH_4]^+$ calcd for $C_{22}H_{18}ClNO_5S\cdot NH_4^+$, 461.0932; found 461.0939. HPLC (*n*-hexane/*i*-PrOH = 75/25, 210 nm, 1.2 mL/min) Chiralcel AD-H, t_R = 13.05 min (2*R*,3*R*), 14.22 min (2*S*,3*S*), 14.80 min (2*S*,3*R*), 15.67 min (2*R*,3*S*).

(2*R*,3*S*)-4-Nitro-3-(4-nitrophenyl)-1-phenyl-2-(phenylsulfonyl)butan-1-one (**8h**). Yield: 65 %, pale yellow crystals, m.p. 175−176 °C (ethanol), $[\alpha]_D^{20}$ +24.8° (*c* 1.0, $CHCl_3$). ^{1}H NMR (400 MHz, $CDCl_3$, 298 K) δ [ppm] 8.09 (d, 2H, $^{3}J_{HH}$ 8.0 Hz), 7.69 (d, 2H, $^{3}J_{HH}$ 8.0 Hz), 7.62−7.50 (m, 4H, aromatic), 7.42−7.32 (m, 6H, aromatic), 5,51 (d, 1H, H-2, $^{3}J_{HH}$ 5.8 Hz), 5.26 (dd, 1H, H-4, $^{2}J_{HH}$ 12.8 Hz, $^{3}J_{HH}$ 3.2 Hz), 5.11 (dd, 1H, H-4, $^{2}J_{HH}$ 12.8 Hz, $^{3}J_{HH}$ 9.6 Hz), 4.75−4.65 (m, 1H, H-3); ^{13}C NMR (101 MHz, $CDCl_3$, 298 K) δ [ppm] 191.53 (C-1), 148.03, 143.13, 137.41, 136.64, 134.94, 134.85, 129.49, 129.20, 129.06, 128.69, 124.51, 76.29 (C-4), 70.67 (C-2), 42.34 (C-3); IR (neat) ν [cm^{-1}] 3061 w, 2982 w, 2945 w, 2839 w, 1672 s, 1595 m, 1584 m, 1551 vs, 1489 m, 1466 w, 1446 s, 1385 m, 1321 s, 1308 s, 1296 s, 1271 s, 1248 s, 1161 m, 1138 s, 1082 s, 1061 m, 1034 m, 970 m, 889 w, 866 m, 847 m, 800 m, 787 w, 752 vs, 727 m, 702 s, 683 vs, 656 w, 627 m, 610 m, 588 m, 565 m, 515 vs, 422 m; APPI-HRMS (*m*/*z*): $[M-H]^-$ calcd for $C_{22}H_{18}N_2O_7S\cdot NH_4^+$ 453.0762; found 453.0767. HPLC (*n*-hexane/*i*-PrOH = 50/50, 210 nm, 1.2 mL/min) Chiralcel AD-H, t_R = 13.39 min (2*R*,3*S*), 15.60 min (2*S*,3*R*).

3-(3-Methoxyphenyl)-4-nitro-1-phenyl-2-phenylsulfonyl)butan-1-one (a mixture of diastereomers (2*R*,3*S*)-**8i** and (2*R*,3*S*)-**9i**).

Yield: 52 %, white solid, m.p. 137−139 °C. $[\alpha]_D^{20}$ -16.5° (*c* 1.0, $CHCl_3$). ^{1}H NMR (400 MHz, $CDCl_3$, 298 K) δ [ppm] 7.80−7.72 (m, 4H, **8i**,**9i**), 7.60−7.55 (m, 5H, **8i**), 7.50−7.40 (m, 8H, **8i**,**9i**), 7.35−7.30 (m, 1H, **8i**), 7.25−7.20 (m, 1H), 7.15−7.05 (m, 1H, **8i**), 7.02−6.99 (m, 1H, **8i**), 6.75−6.70 (m, 1H, **8i**), 6.70−6.65 m (m, 1H, **9i**), 6.65−6.55 (m, 4H, **8i**,**9i**), 5.62 (d, 1H, H-2, $^3J_{HH}$ 11.0 Hz, **9i**), 5.55 (dd, 1H, H-4, $^2J_{HH}$ 13.5 Hz, $^3J_{HH}$ 4.1 Hz, **9i**), 5.44 (d, 1H, H-2, $^3J_{HH}$ 4.8 Hz, **8i**), 5.32 (dd, 1H, H-4, $^2J_{HH}$ 14.0 Hz, $^3J_{HH}$ 3.4 Hz, **8i**), 5.10 (dd, 1H, H-4, $^2J_{HH}$ 13.5 Hz, $^3J_{HH}$ 10.3 Hz, **9i**), 5.07 (dd, 1H, H-4, $^2J_{HH}$ 14.0 Hz, $^3J_{HH}$ 10.3 Hz, **8i**), 4.53−4.49 (m, 1H, H-3, **8i**), 4.40 dt (1H, H-3, $^3J_{HH}$ 3.9 Hz, $^3J_{HH}$ 11.0 Hz, **9i**), 3.63 (s, 3H, CH_3O, **8i**), 3.59 (s, 3H, CH_3O, **9i**); ^{13}C NMR (101 MHz, $CDCl_3$, 298 K) δ [ppm] 190.91 (C-1, **8i,9i**), 160.14 (**8i)**, 159.85 (**9i**), 137.53 (**8i**), 137.21 (**8i**), 136.70 (**9i**), 136.47 (**9i**), 136.43 (**9i**), 134.89 (**9i**), 134.39 (**8i**), 133.85 (**9i**), 130.53 (**8i**), 130.15 (**8i,9i**), 129.96 (**9i**), 129.41 (**8i**), 129.17 (**8i**), 129.24 (**9i**), 128.85 (**8i**), 128.68 (**8i**), 128.58 (**9i**), 128.29 (**9i**), 78.08 (**9i**), 76.49 (**8i**), 71.00 (C-4, **9i)**, 71.29 (C-4, **8i)**, 55.21 (C-2, **9i)**, 55.25 (C-2, **8i)**, 43.34 (C-3, **9i**), 42.66 (C-3, **8i**); APPI-HRMS (*m*/*z*): $[M+NH_4]^+$ calcd for $C_{23}H_{21}NO_6S{\cdot}NH_4^+$, 457.1428; found 457.1436. HPLC (*n*-hexane/*i*-PrOH = 80/20, 210 nm, 1.2 mL/min) Chiralcel AD-H, t_R = 17.63 min (2*S*,3*S*), 19.93 min (2*R*,3*S*), 21.03 min (2*R*,3*R*), 25.28 min (2*S*,3*R*).

The study of the evolution of dr during the reaction of sulfone 5a with ω-nitrostyrene (6a) in the presence of catalyst 7a

To study the evolution of the reaction, 0.25 mmol of **5a**, 0.25 mmol of **6a** and 0.08 mmol of mesitylene as internal standard were dissolved in 0.75 mL of chloroform-*d* in an NMR tube. The 0.1 M solution of the complex **7a** in chloroform-*d* (50 μL, 0.005 mmol, 2 mol % of **7a**) was added and monitored by ^{1}H NMR at 25 °C. Conversion was determined by

reducing the integral intensity of signal of the methylene group of sulfone **5a** at 4.75 ppm. The diastereomers **8a**/**9a** ratio was determined by the ratio of the integral intensities of signals of methine groups of **8a** and **9a** at 4.62–4.57 and 4.53–4.47 ppm, respectively (see **Figure S1**, **Table S1**).

A

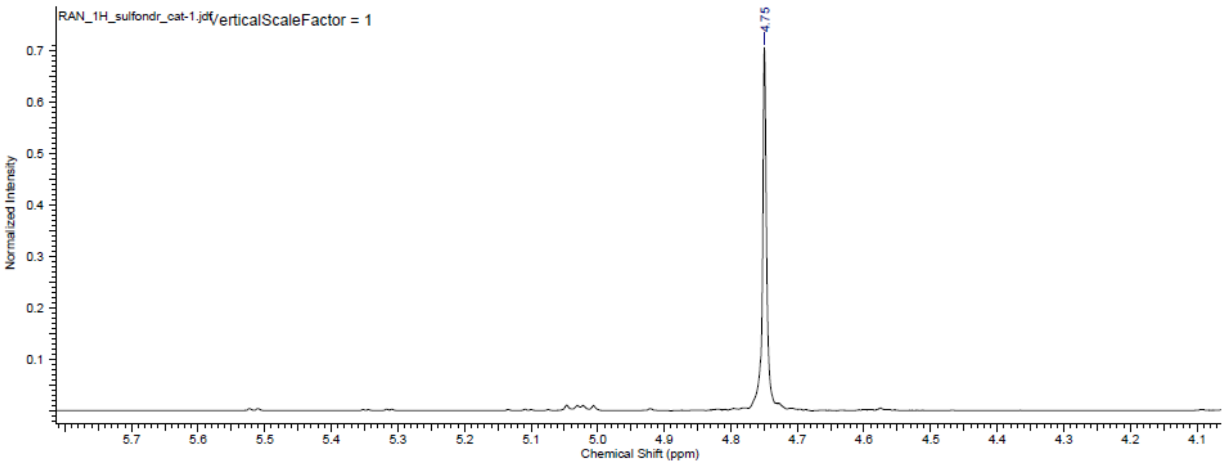

B

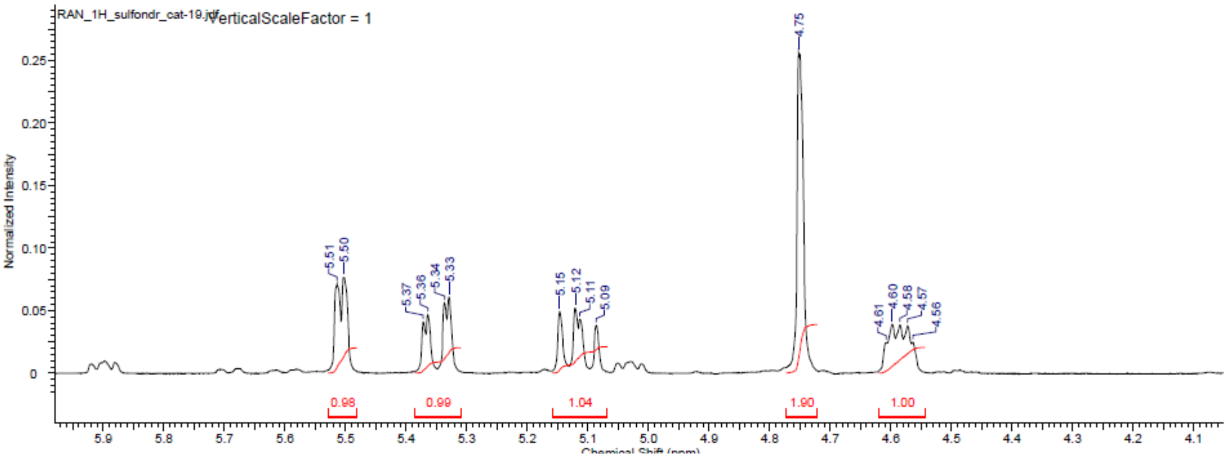

C

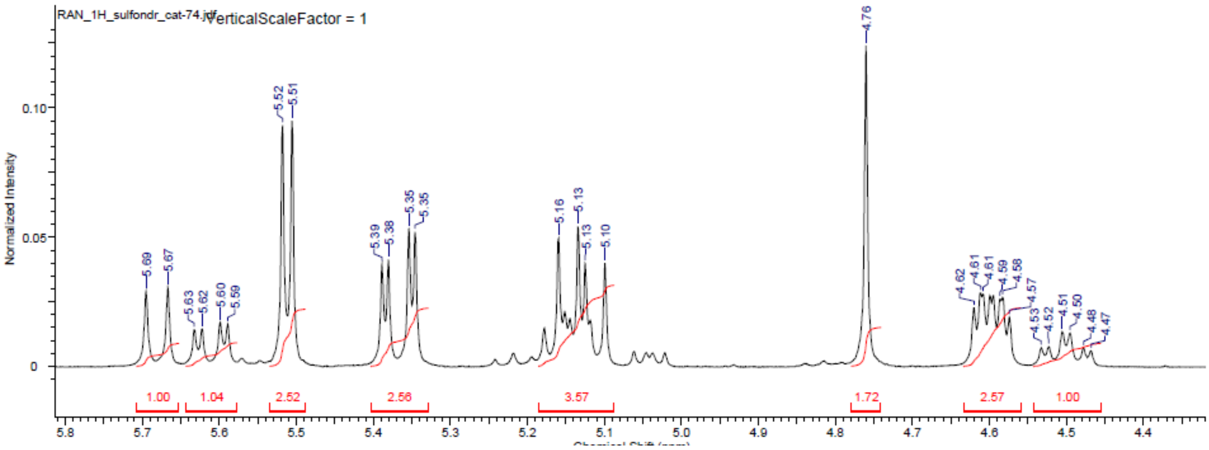

Figure S1. Fragments of the ^{1}H NMR spectra of the reaction mixture after 10 min (**A**), 6 h (**B**) and 81 h (**C**).

Table S1. Evolution of the conversion **5a** and diastereomeric composition of the products of reaction of **5a** with **6a** in the presence of catalyst **7a** (2 mol %) in chloroform-*d*.

Entry	Time, h	Conversion of **5a**, %	Content of **9a** in reaction products, %	Entry	Time, h	Conversion of **5a**, %	Content of **9a** in reaction products, %
1	0.167	0	0	22	16	76.3	9.2
2	0.5	4.5	0	23	17	77.3	9.5
3	0.833	7.7	0	24	18	77.7	10.2
4	1.167	13.7	1.7	25	19	78.2	10.4
5	1.5	16.3	2.1	26	20	78.8	11.5
6	2	20.6	3.6	27	21	79	11.4
7	2.333	23.3	3.6	28	22	79.3	12.4
8	2.666	27	4	29	22.5	80.5	12.4
9	3	31.3	4.04	30	24	81.5	13.7
10	4	37.7	4.5	31	26	81.5	13.4
11	5	45.2	5.04	32	28	81.9	15.6
12	6	50.8	5.7	33	32	82.9	18.3
13	7	56	6.3	34	36	83.3	20
14	8	60.2	6.3	35	41	83.2	21.2
15	9	63.8	6.9	36	45	83.6	22.4
16	10	66.3	7.2	37	51	83.8	23.7
17	11	68.9	7	38	57	83.4	24.6
18	12	71.1	7.4	39	63	83.8	25.3
19	13	73	7.9	40	69	83.9	26
20	14	74.1	8.8	41	75	84	27.5
21	15	74.1	9	42	81	84.1	27.9

The study of the epimerization and *retro*-Michael reaction of compound (2*R*,3*S*)-8a

In the NMR vial was placed (2*R*,3*S*)-**8a** and chloroform-*d* (0.75 mL). The progress of epimerization was determined by the ratio of the integral intensities of signals of methine groups of **8a** and **9a** at 4.58–4.54 and 4.49–4.42 ppm (see **Figure S2**, **Table S2**). The progress of *retro*-Michael reaction was determined by increase in the integrated signal intensity of the methylene group of sulfone **5a** at 4.74 ppm.

Table S2. Progress of the epimerization and *retro*-Michael reaction of compound (2*R*,3*S*)-**8a**.

Entry	Time, h	Content of **5a**, %	Content of **9a**, %	Entry	Time, h	Content of **5a**, %	Content of **9a**, %
1	4	3.8	0	10	48	15	13.7
2	8	6.5	0	11	54	15.2	14.3
3	12	8.1	4.6	12	60	15.3	16.3
4	16	9.8	6.6	13	66	15.5	16.3
5	20	11.1	6.7	14	73	15.5	17.1
6	24	12	8.2	15	78	15.6	18
7	30	13.3	10.4	16	84	15.2	19.4
8	36	14.1	11.4	17	90	15.3	19.6
9	42	14.5	13.4	18	96	15.3	20.1

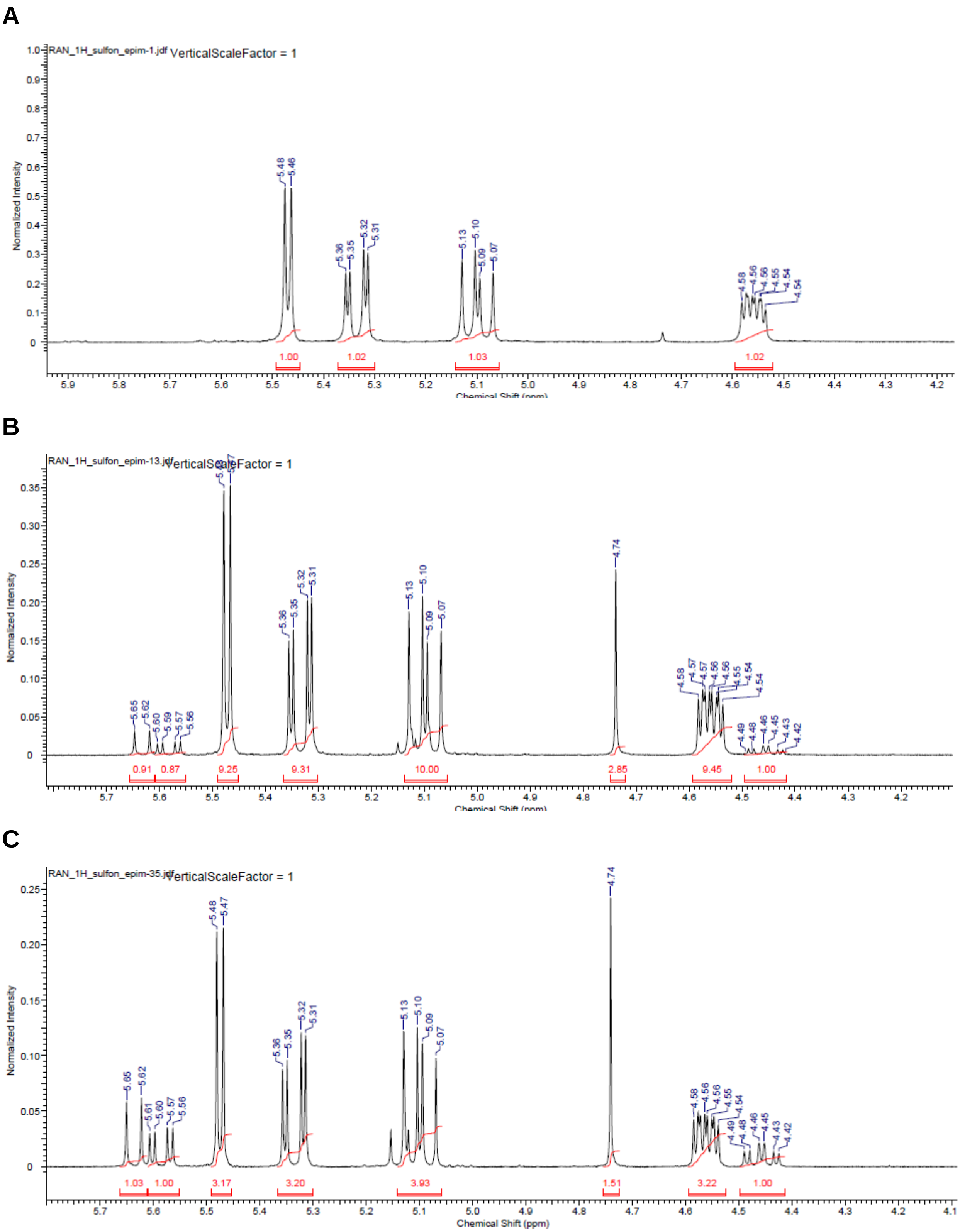

Figure S2. Fragments of the ^{1}H NMR spectra (4.1–5.8 ppm) of the solution of (2*R*,3*S*)-**8a** in chloroform-*d* after 10 min (**A**), 24 h (**B**) and 96 h (**C**).

Copies of NMR, FTIR and mass spectra for compounds 8 and 9

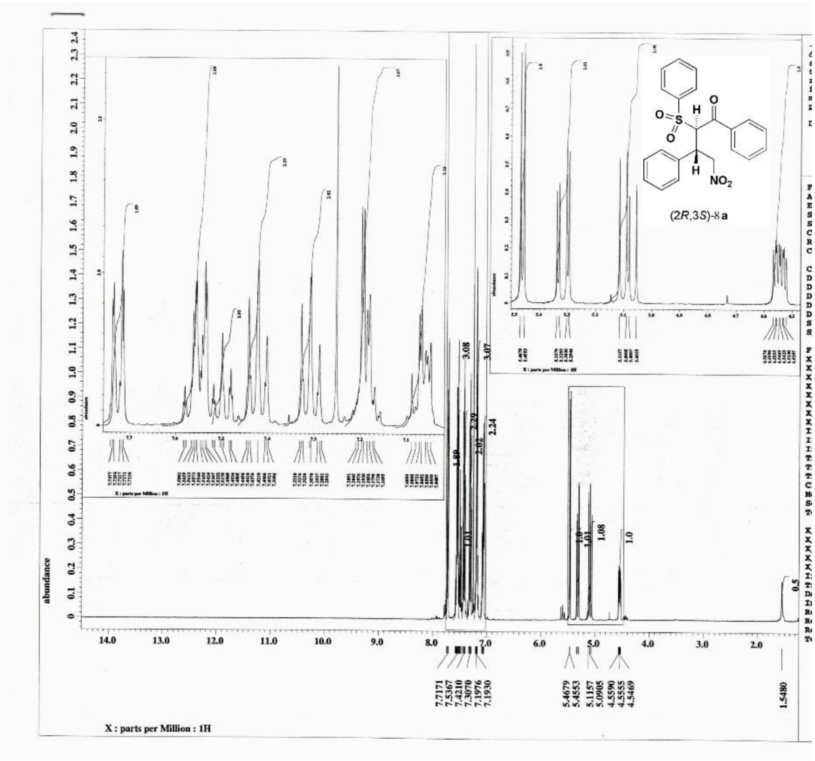

Figure S3.^{1}H NMR spectra of compound **8a** in $CDCl_3$.

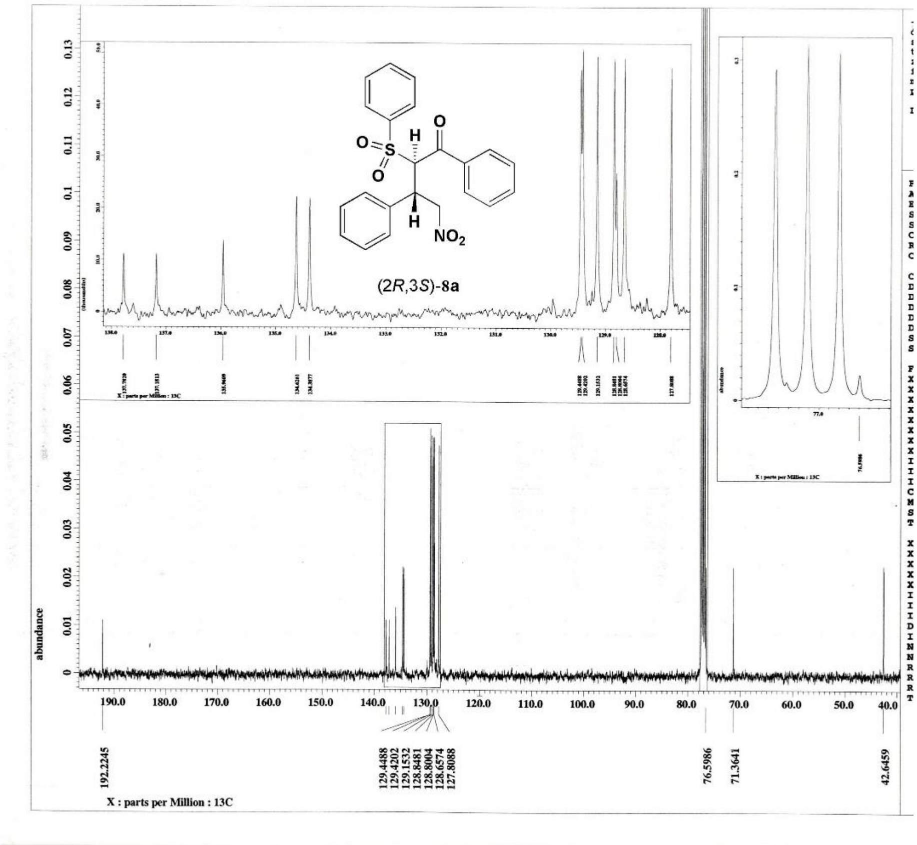

Figure S4.^{13}C NMR spectra of compound **8a** in $CDCl_3$.

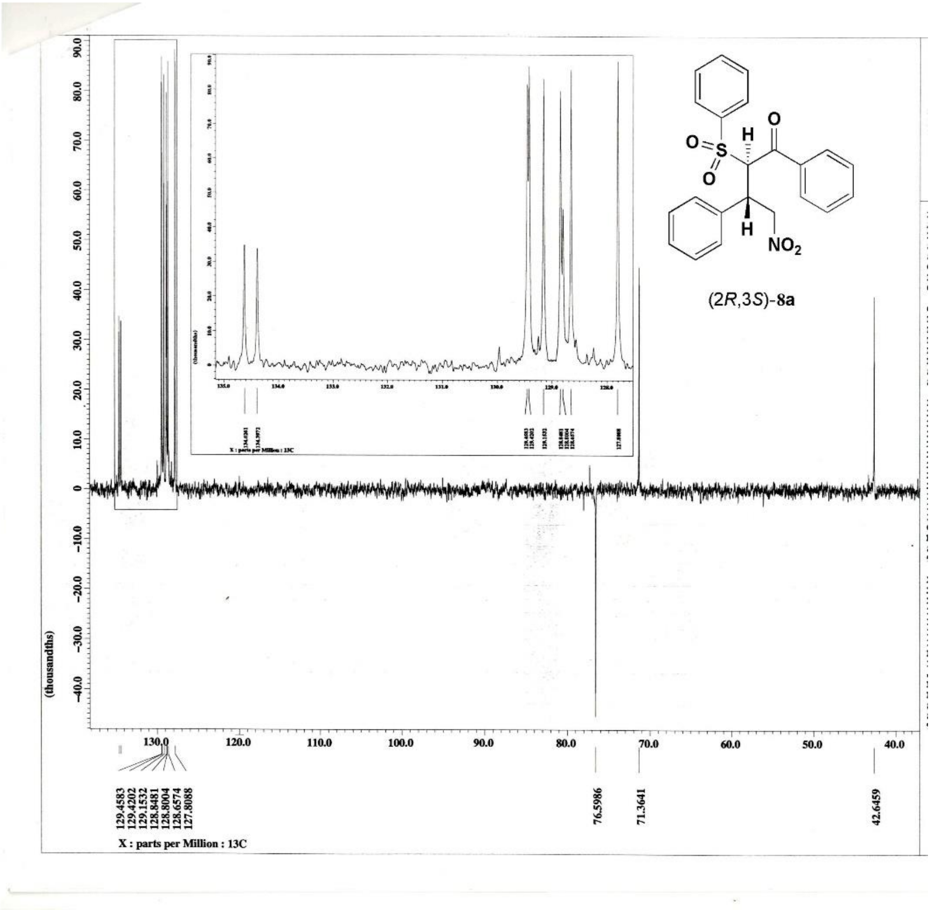

Figure S5. DEPT spectra of compound **8a**.

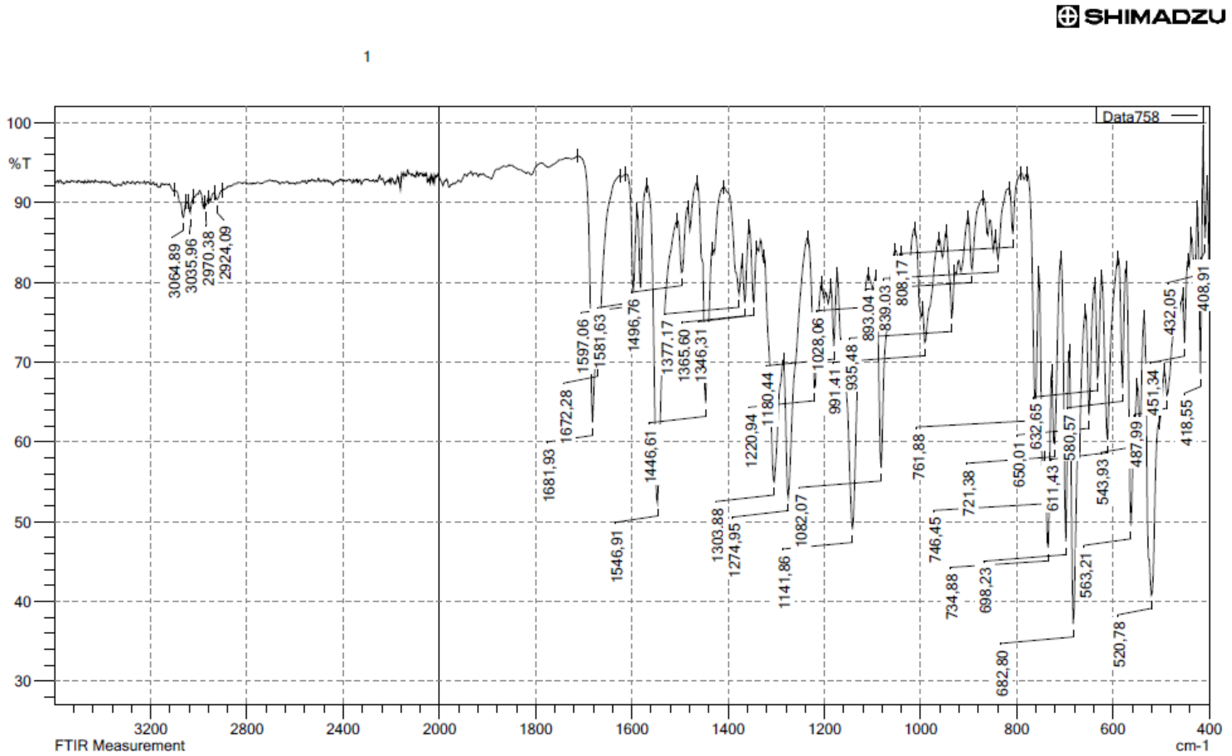

Figure S6. FTIR spectra of compound **8a**.

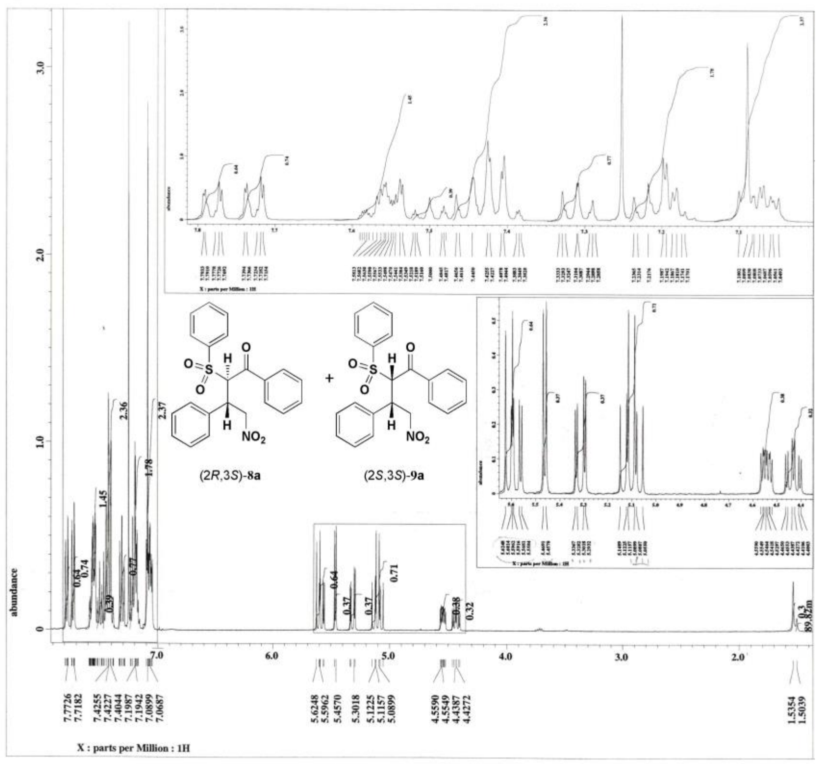

Figure S7. ^{1}H NMR spectra of the mixture of diastereomers **8a** and **9a** in $CDCl_3$.

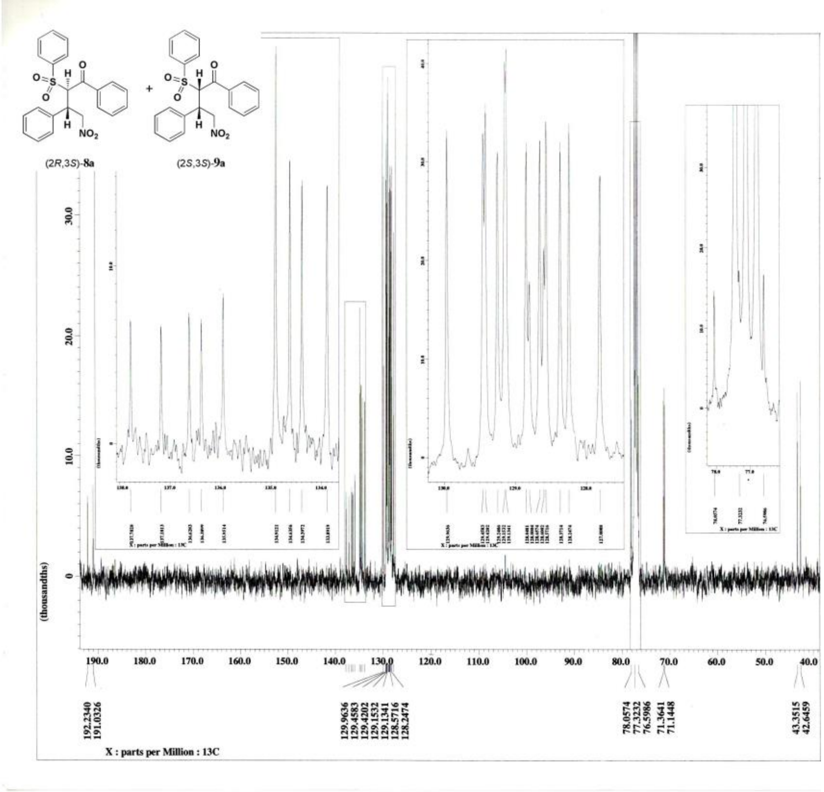

Figure S8. ^{13}C NMR spectra of the mixture of diastereomers **8a** and **9a** in $CDCl_3$.

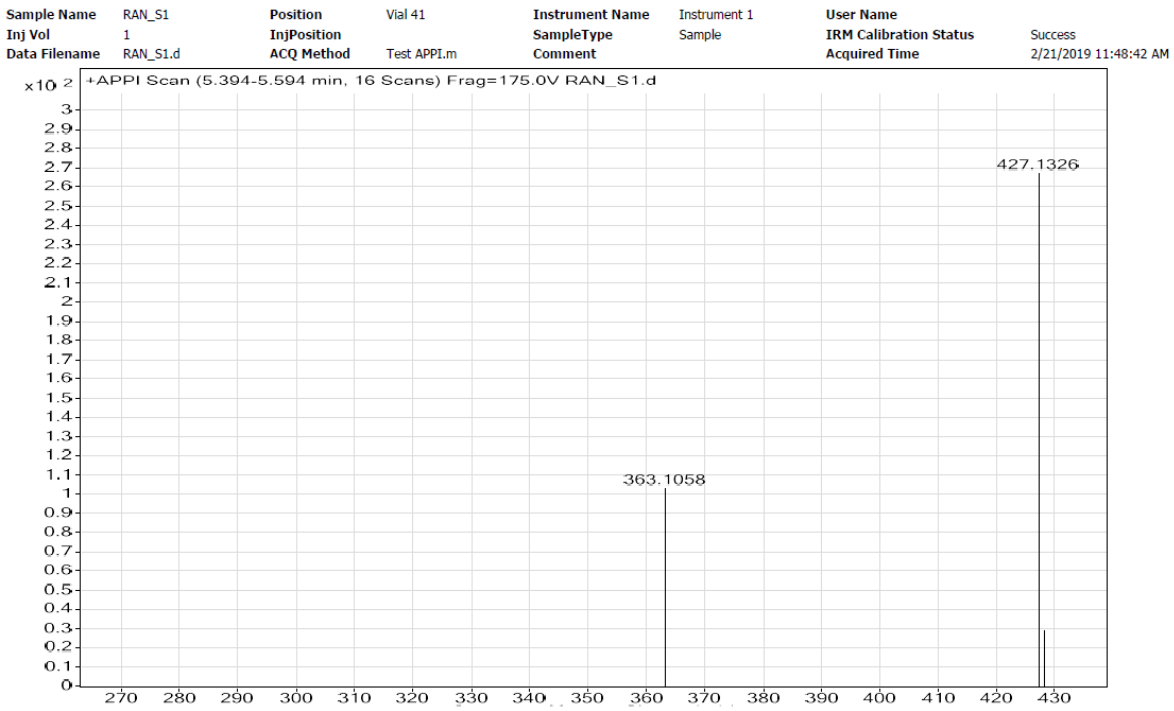

Figure S9. HRMS of compound **8a**.

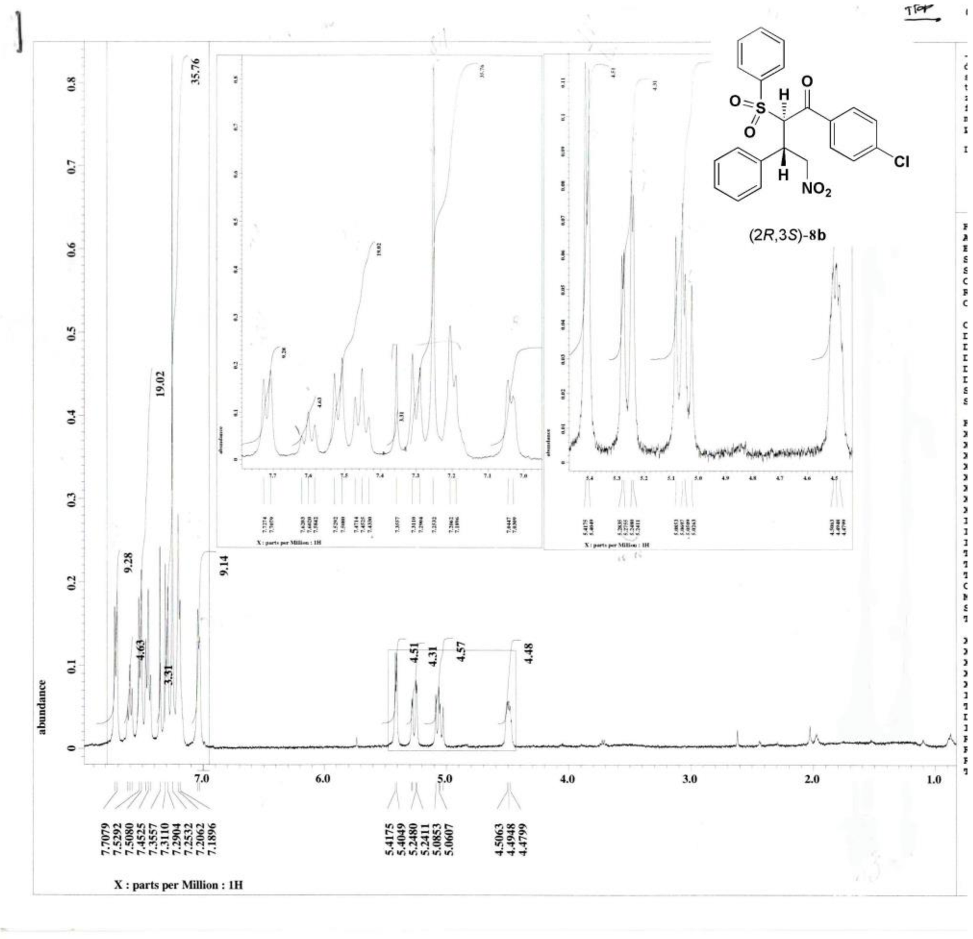

Figure S10. ^{1}H NMR spectra of compound **8b** in $CDCl_3$.

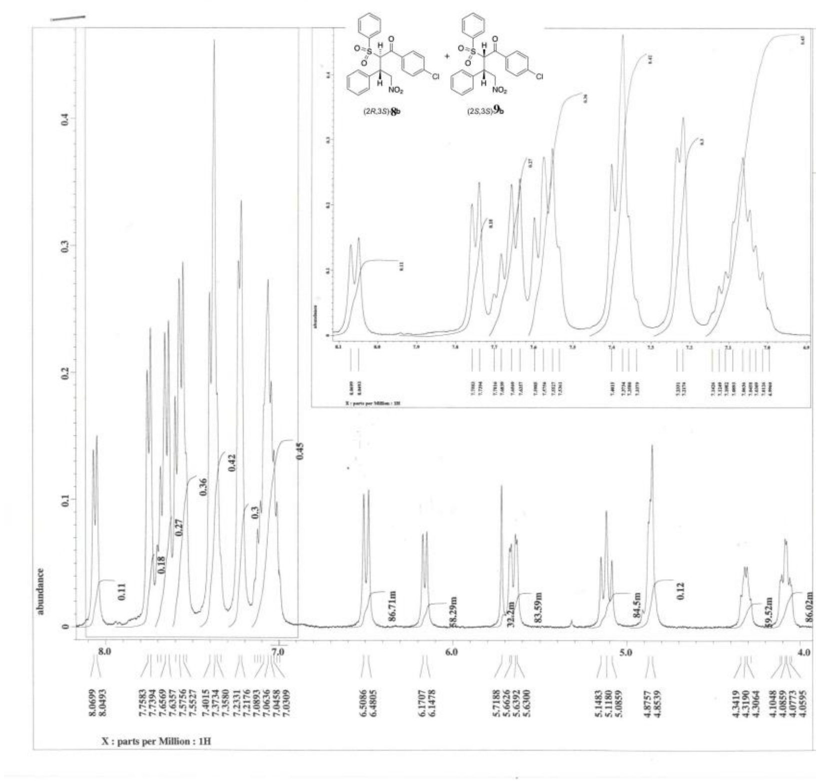

Figure S11. ^{1}H NMR spectra of the mixture of diastereomers **8b** and **9b** in DMSO-d_6.

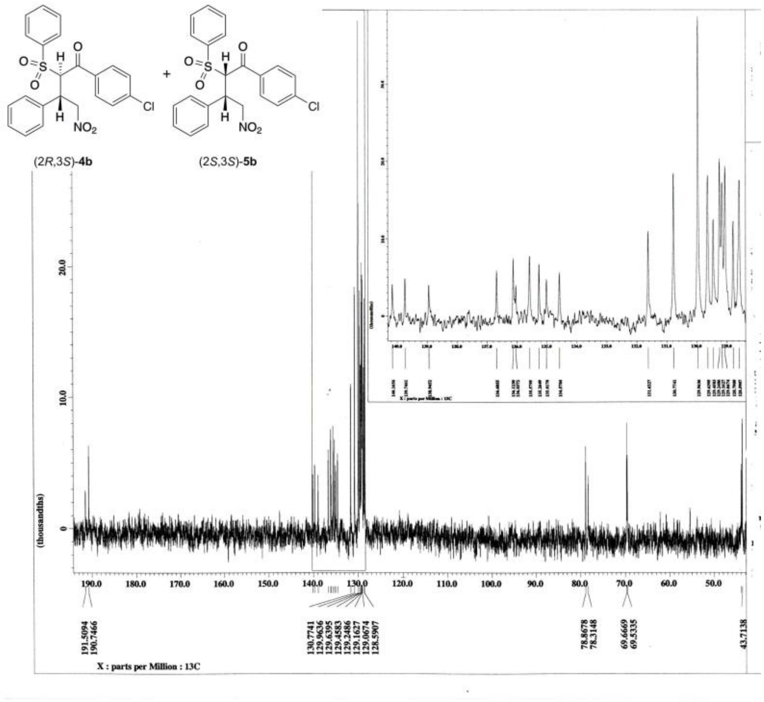

Figure S12. ^{13}C NMR spectra of the mixture of diastereomers **8b** and **9b** in DMSO-d_6.

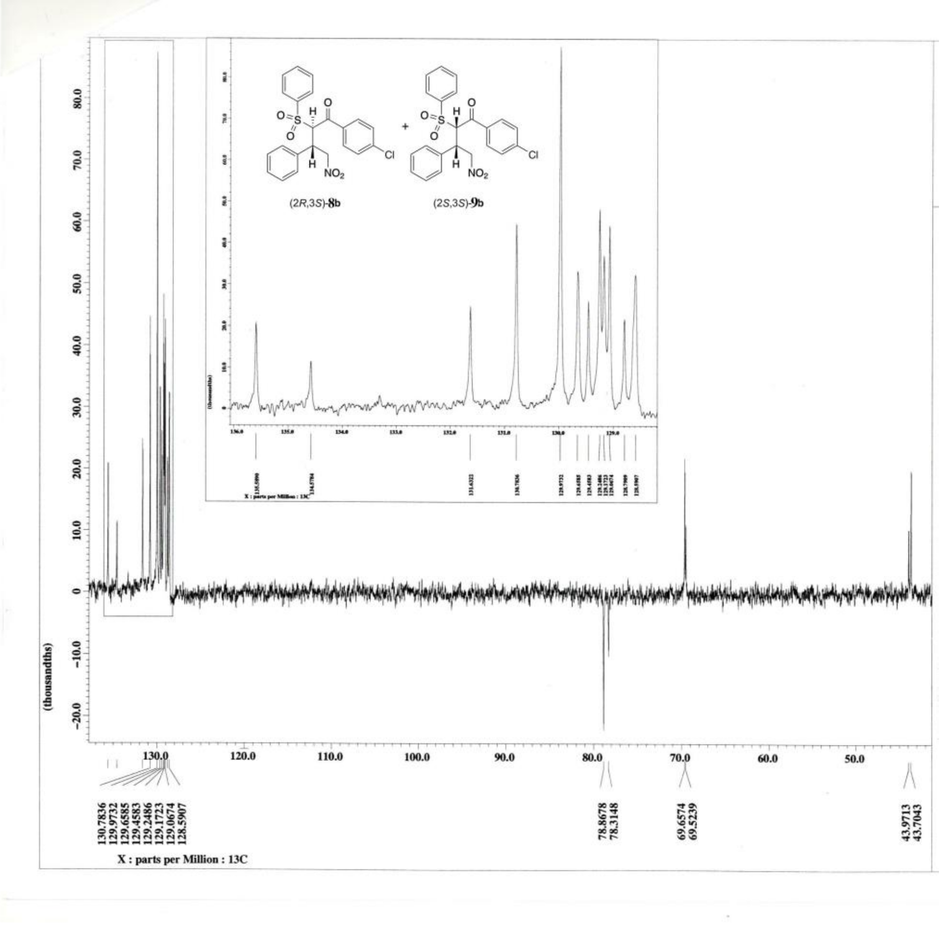

Figure S13. DEPT spectra of the mixture of diastereomers **8b** and **9b** in DMSO-d_6.

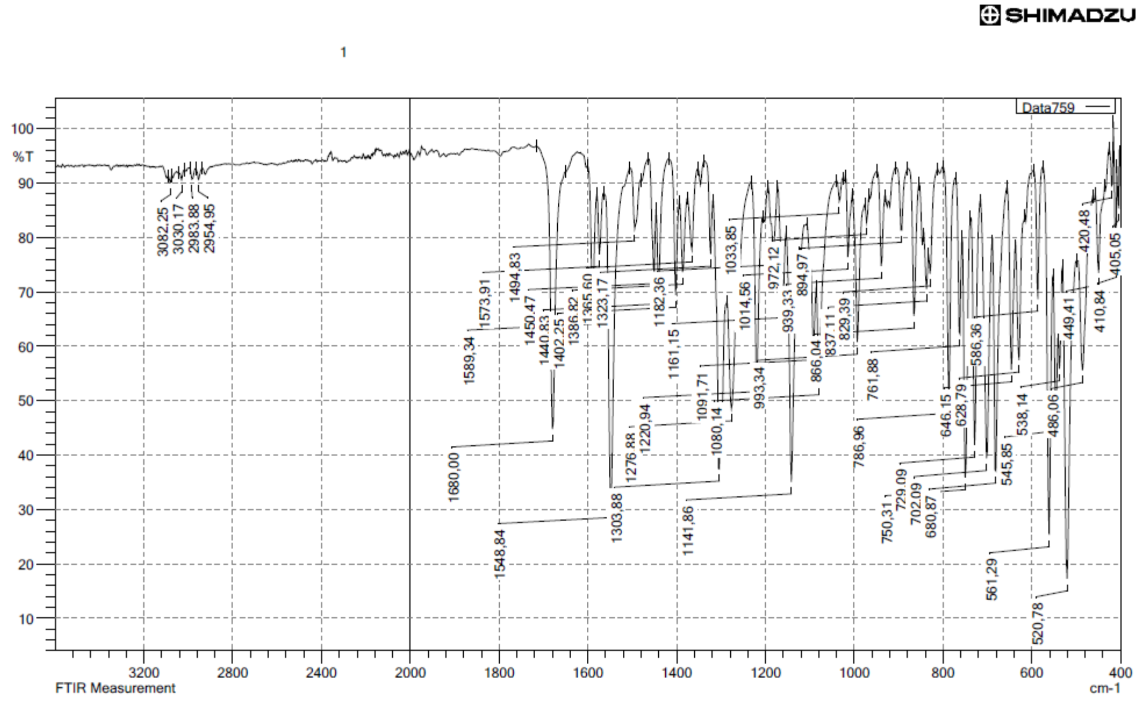

Figure S14. FTIR spectra of compound **8b**.

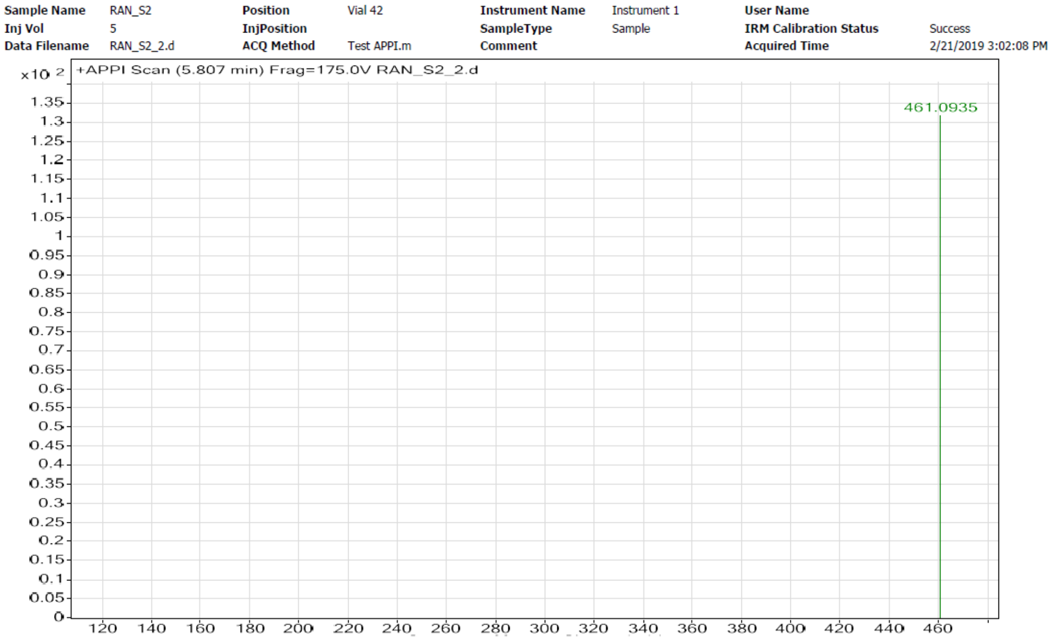

Figure S15. HRMS of compound **8b**.

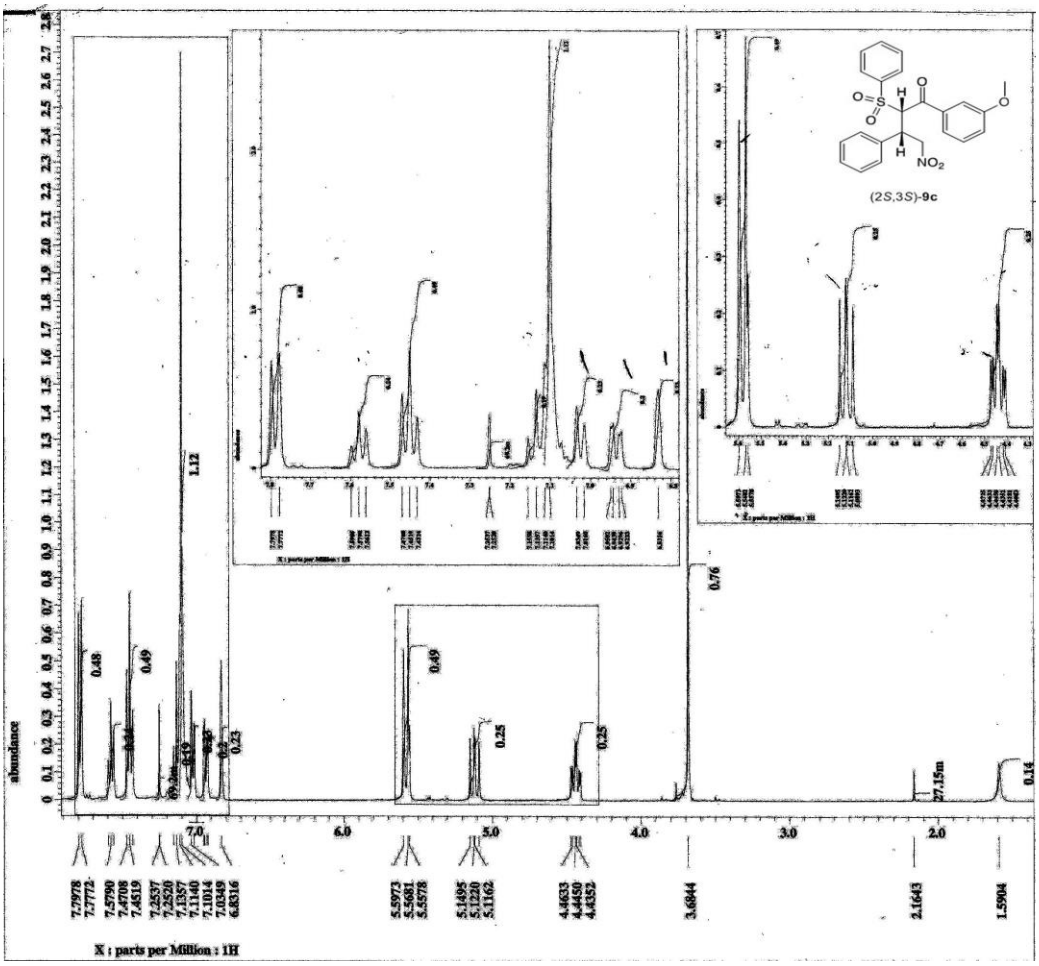

Figure S16. ^{1}H NMR spectra of compound **9c** in $CDCl_3$.

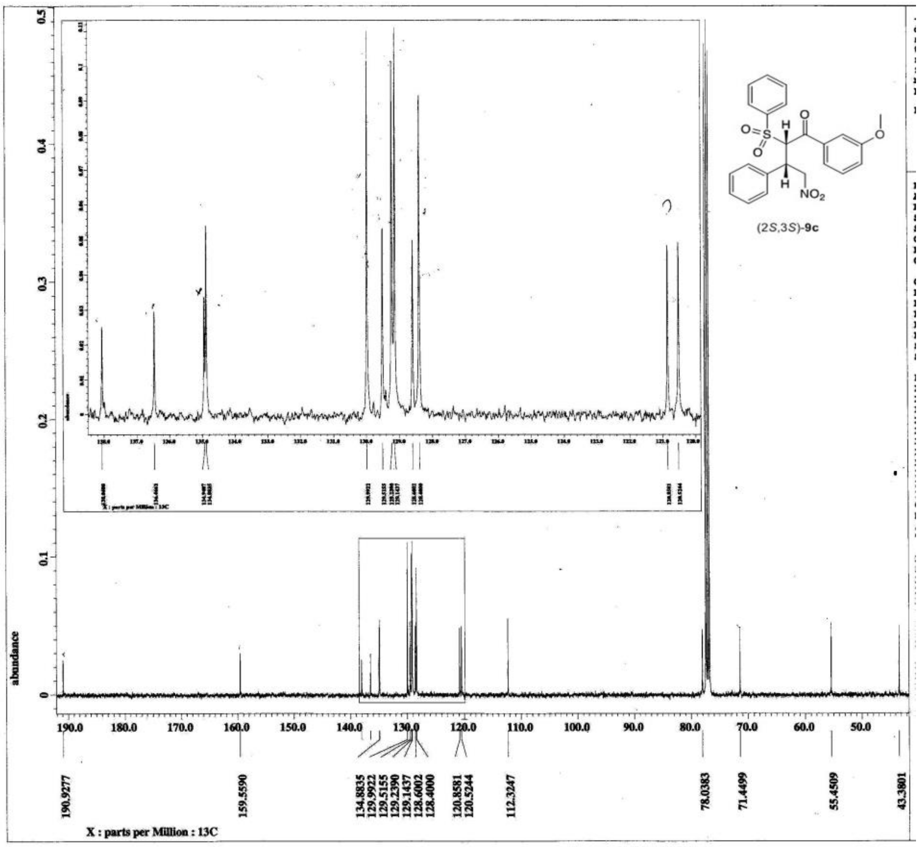

Figure S17. ^{13}C NMR spectra of compound **9c** in $CDCl_3$.

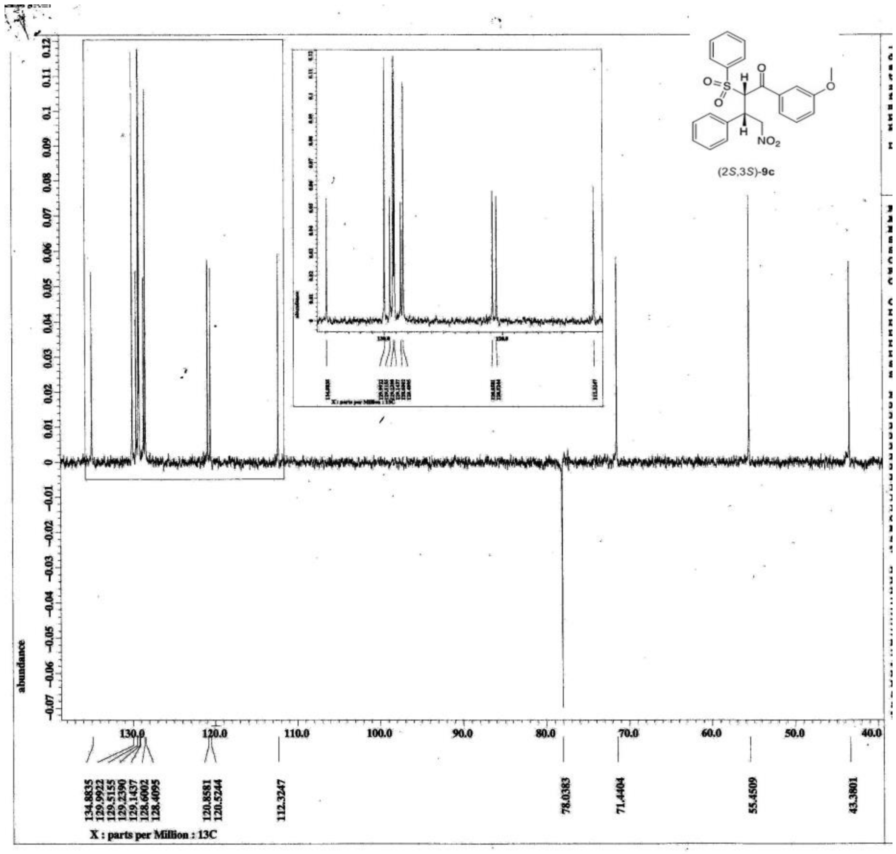

Figure S18. DEPT spectra of compound **9c**.

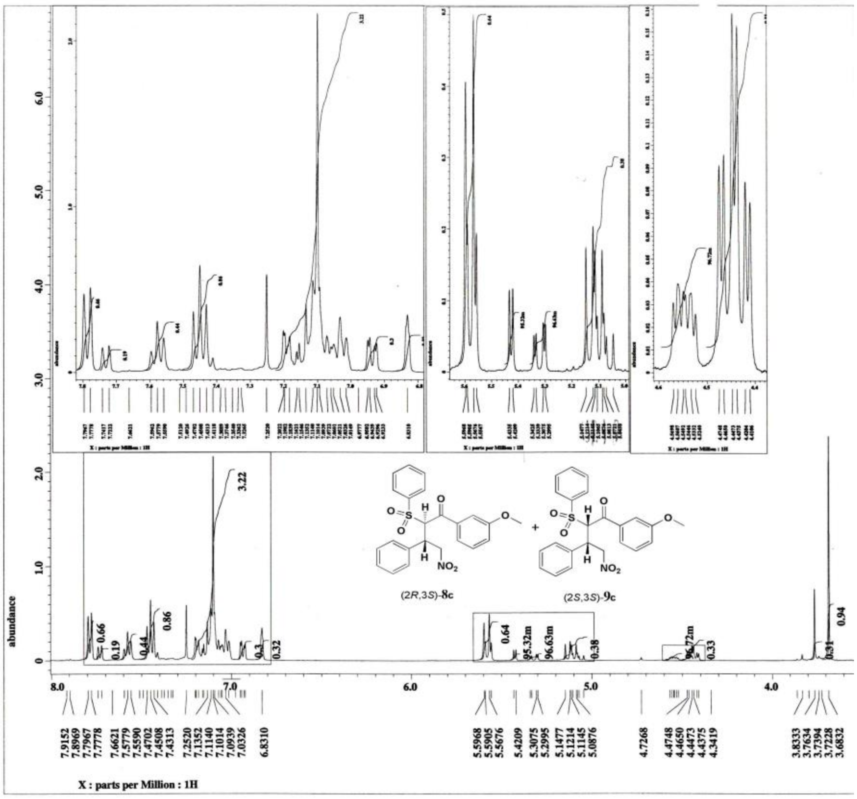

Figure S19. ^{1}H NMR spectra of the mixture of diastereomers **8c** and **9c** in $CDCl_3$.

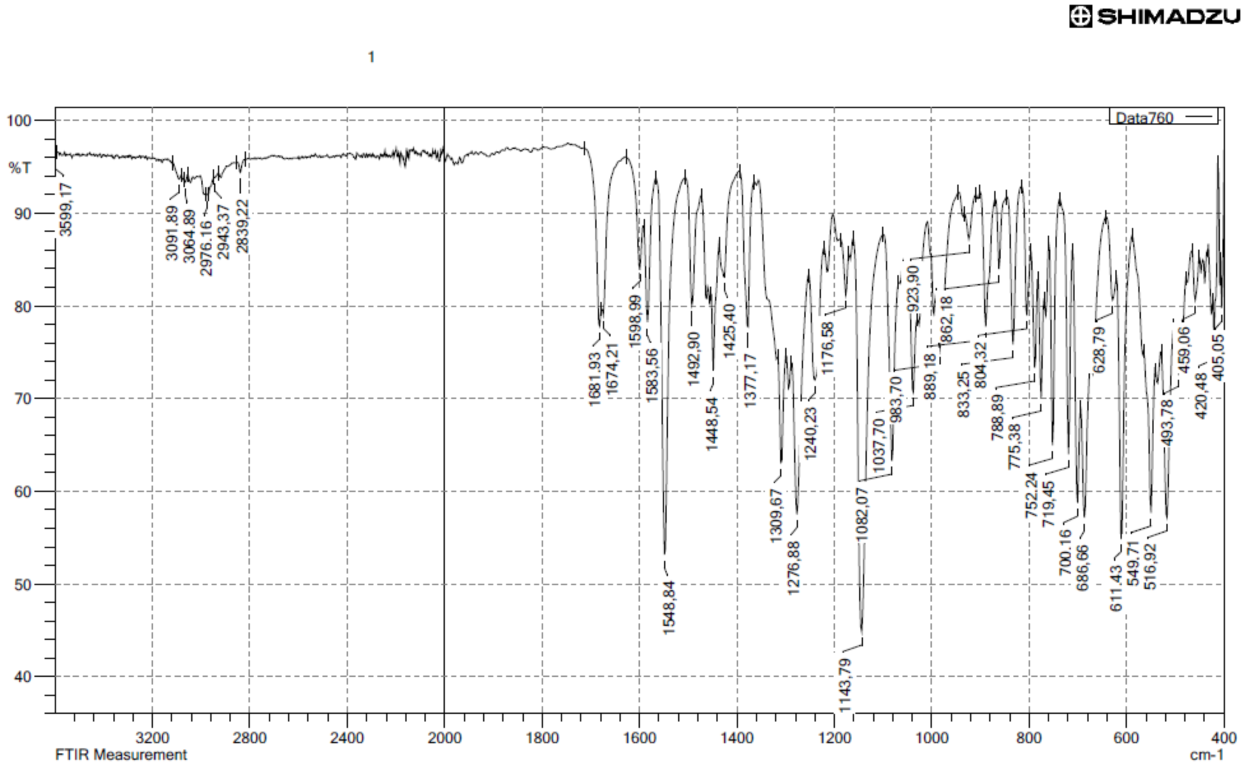

Figure S20. FTIR spectra of compound **9c**.

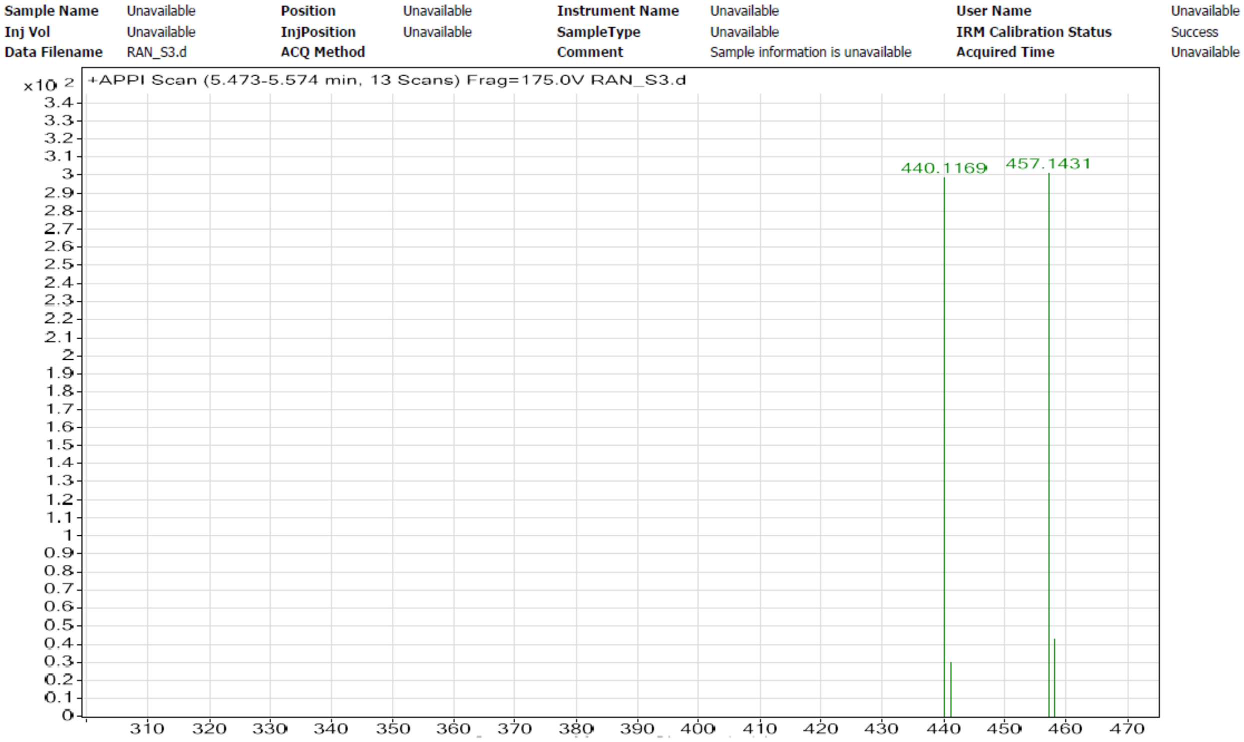

Figure S21. HRMS of compound **9c**.

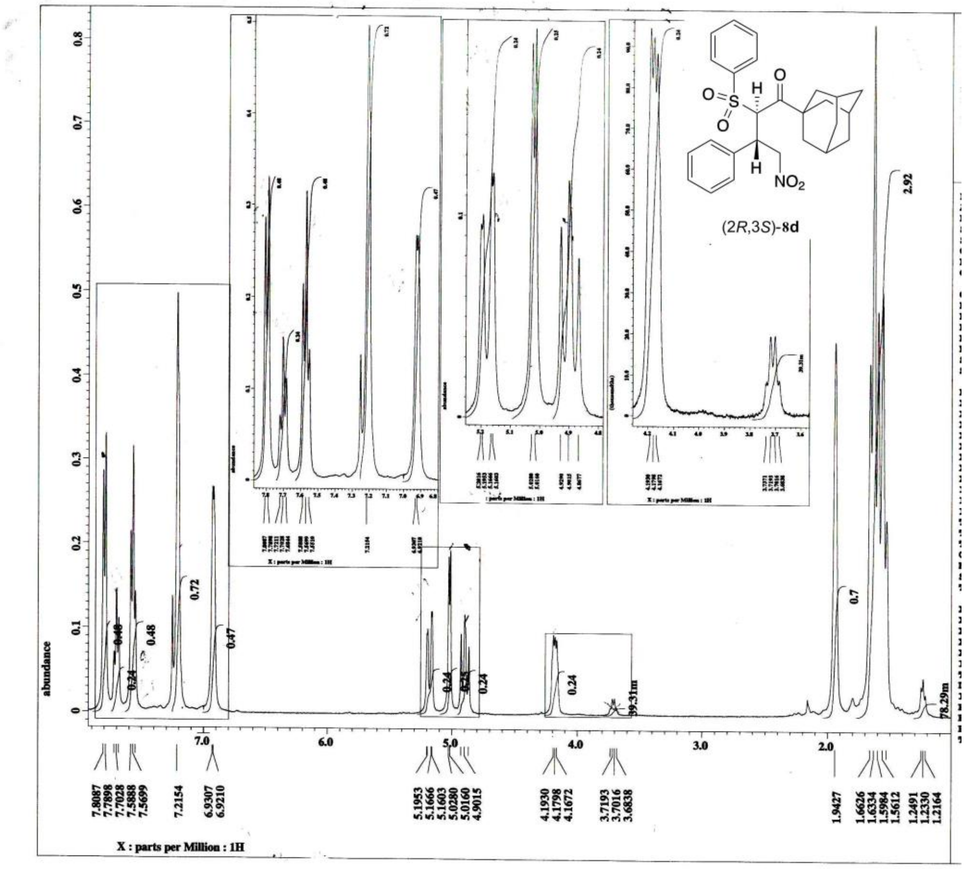

Figure S22. ^{1}H NMR spectra of compound **8d** in $CDCl_3$.

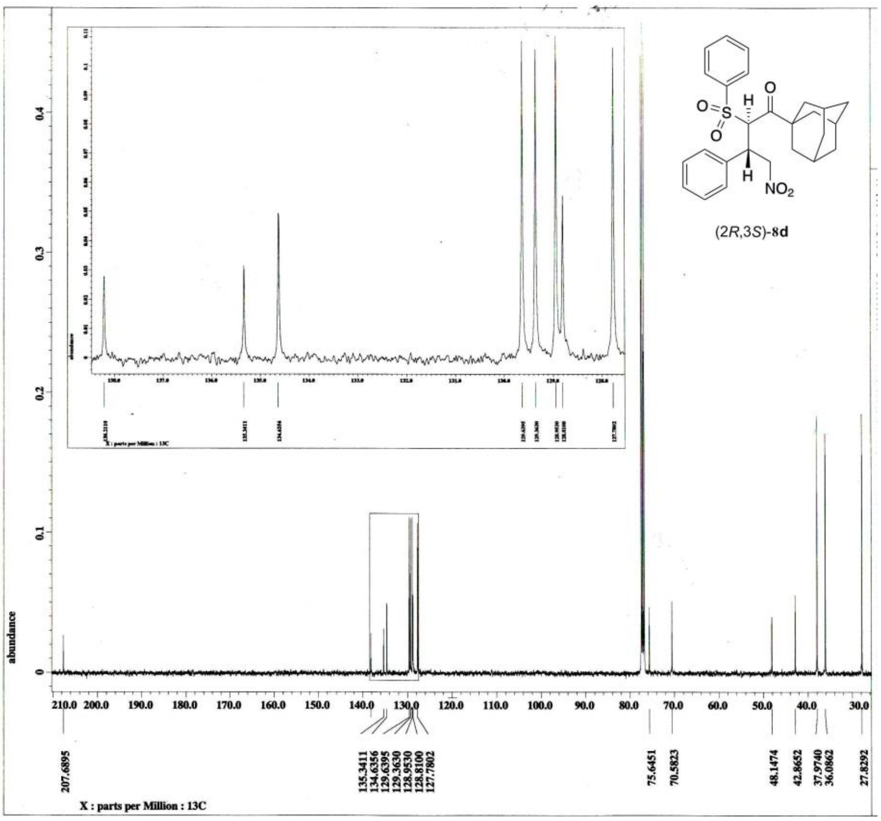

Figure S23. ^{13}C NMR spectra of compound **8d** in $CDCl_3$.

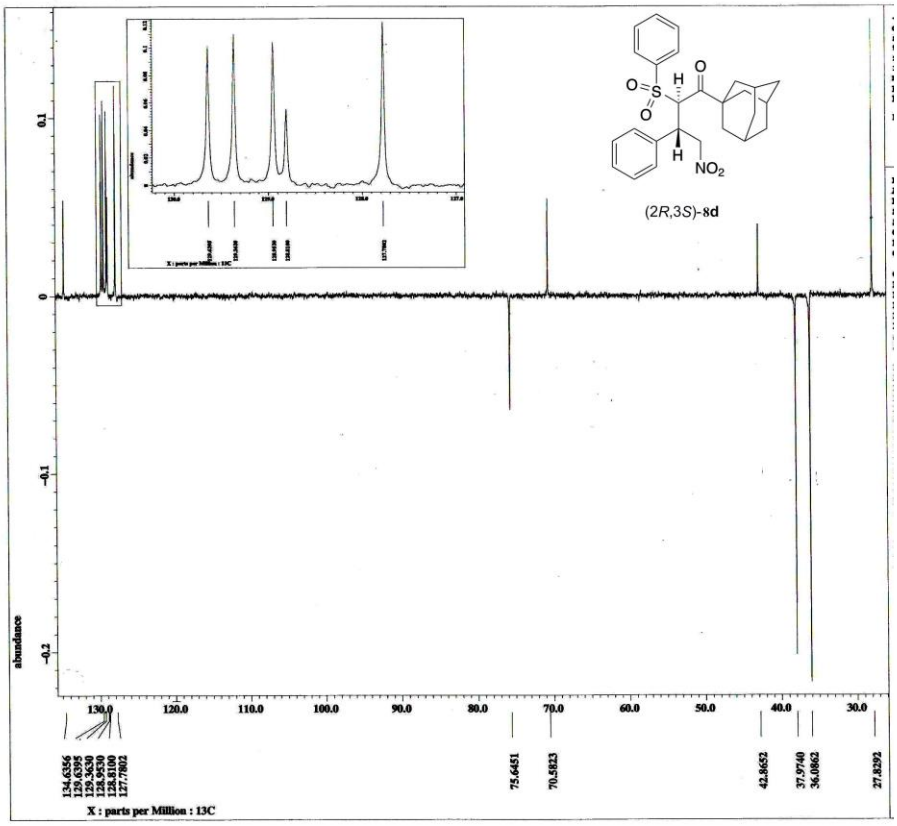

Figure S24. DEPT spectra of compound **8d** in $CDCl_3$.

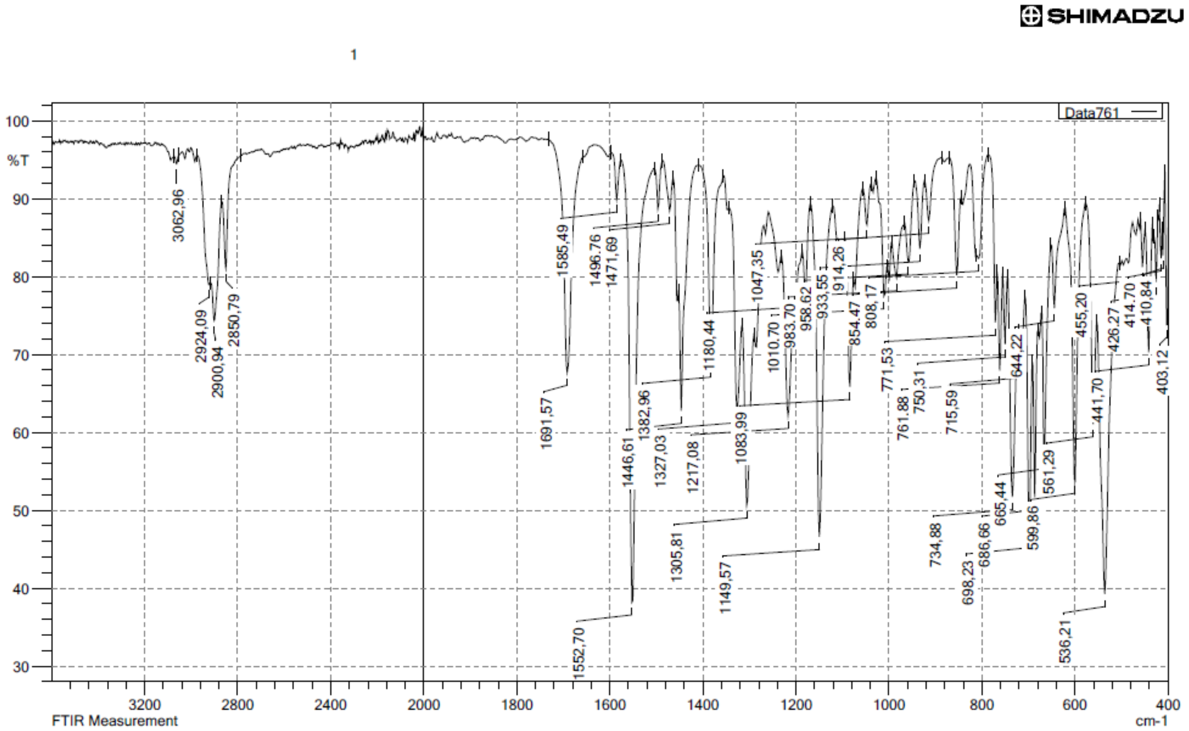

Figure S25. FTIR spectra of compound **8d**.

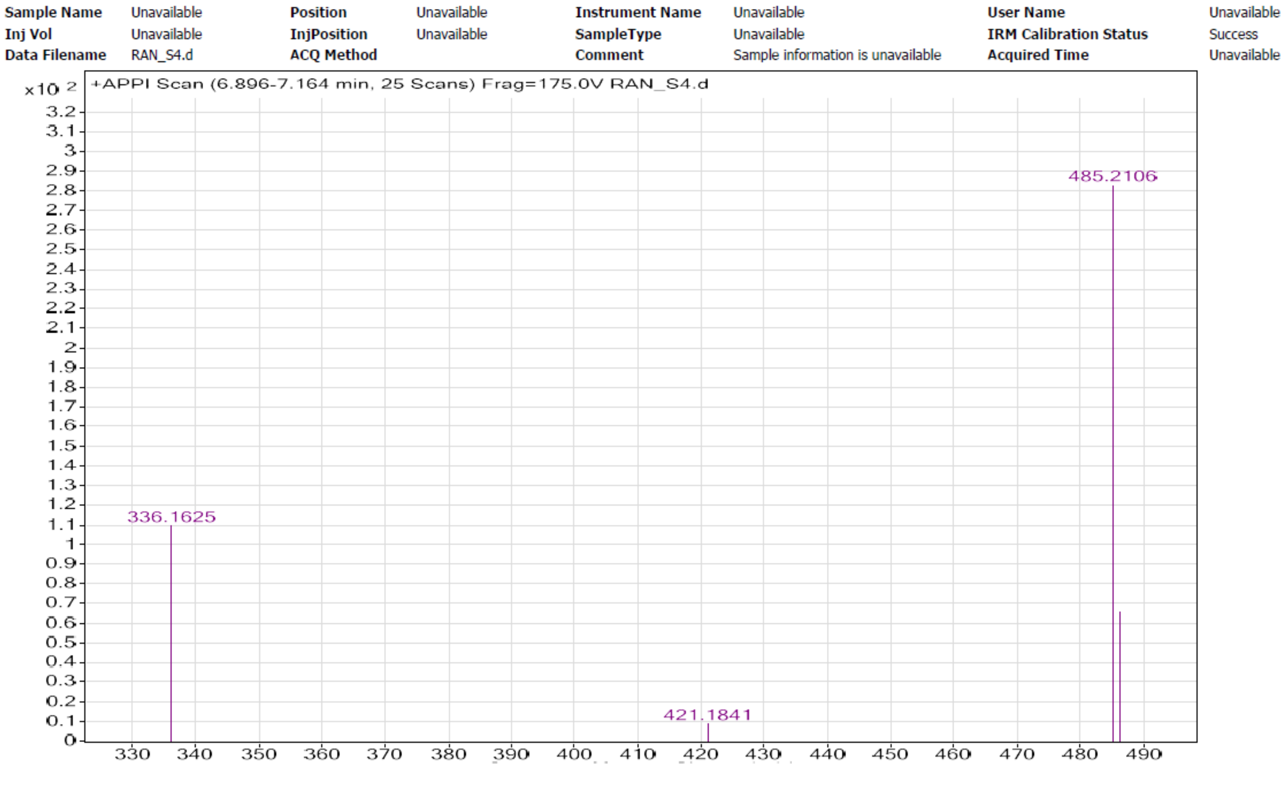

Figure S26. HRMS of compound **8d**.

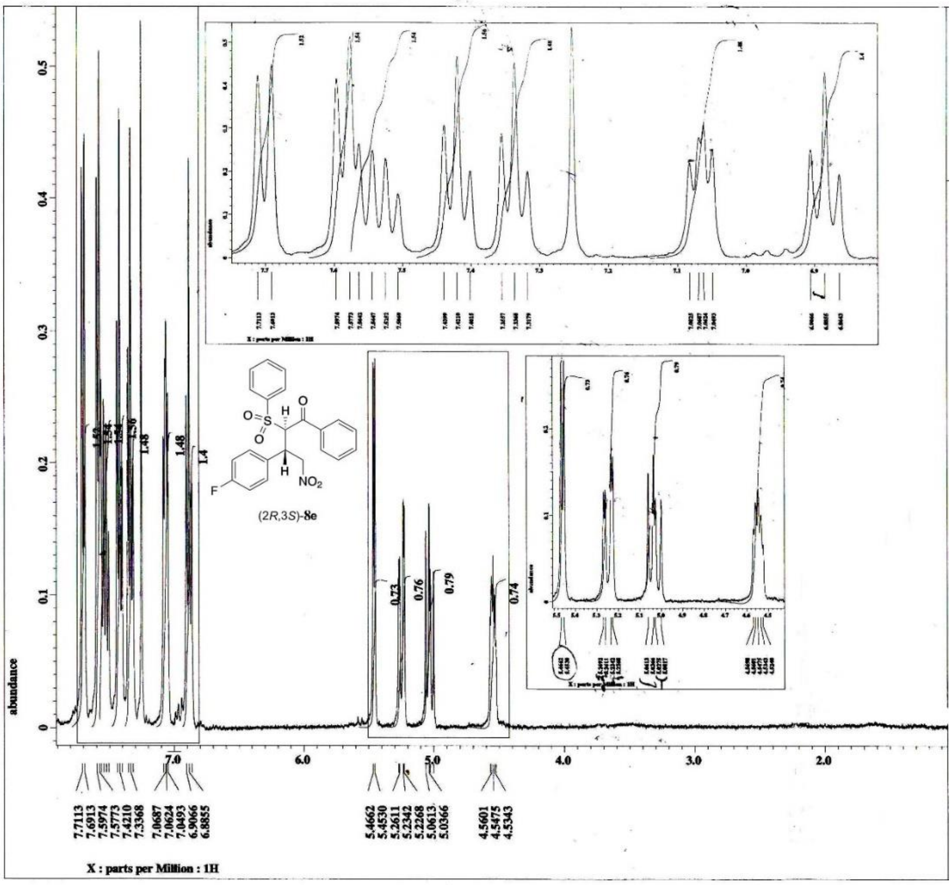

Figure S27. ^{1}H NMR spectra of compound **8e** in $CDCl_3$.

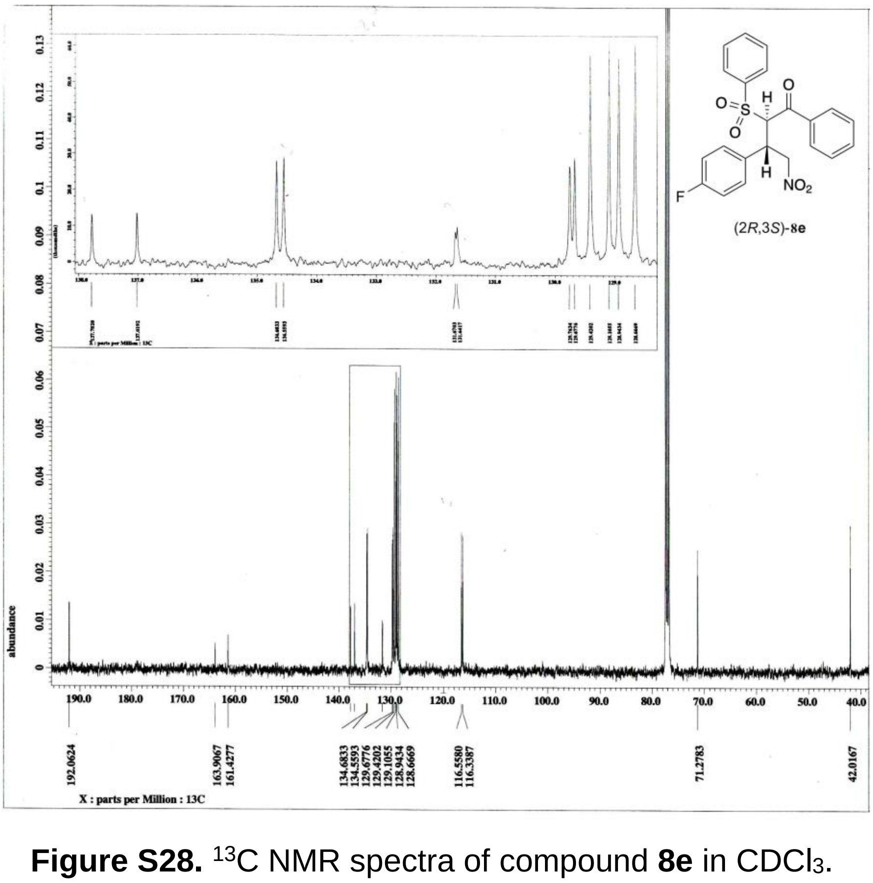

Figure S28. ^{13}C NMR spectra of compound **8e** in $CDCl_3$.

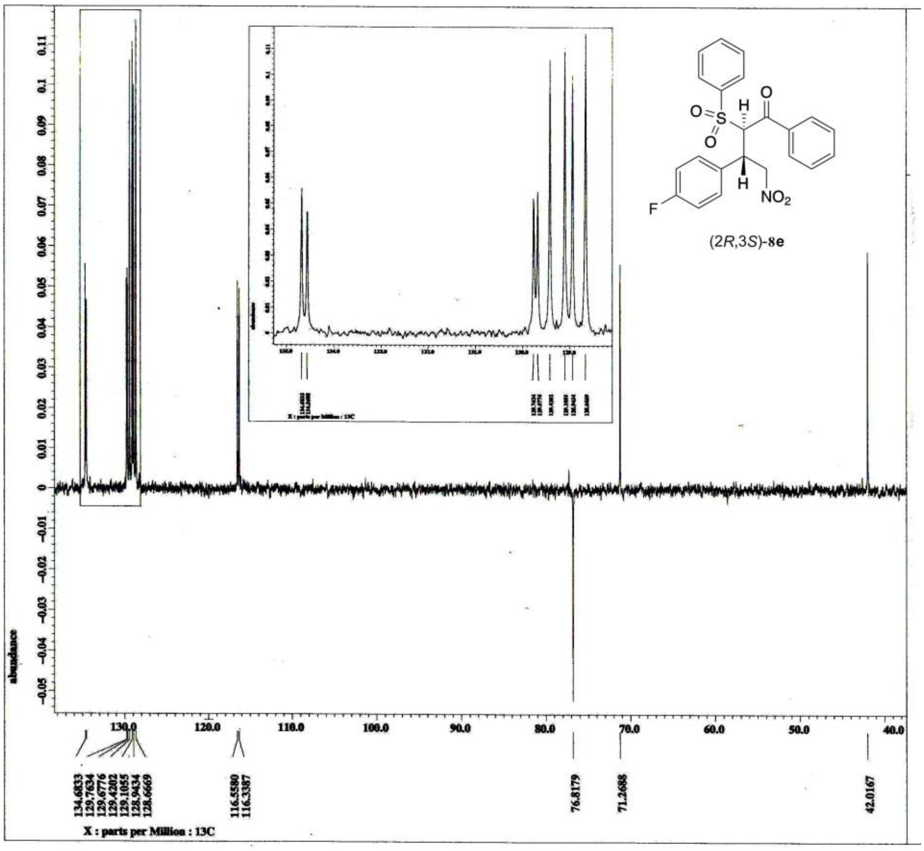

Figure S29. DEPT spectra of compound **8e** in $CDCl_3$.

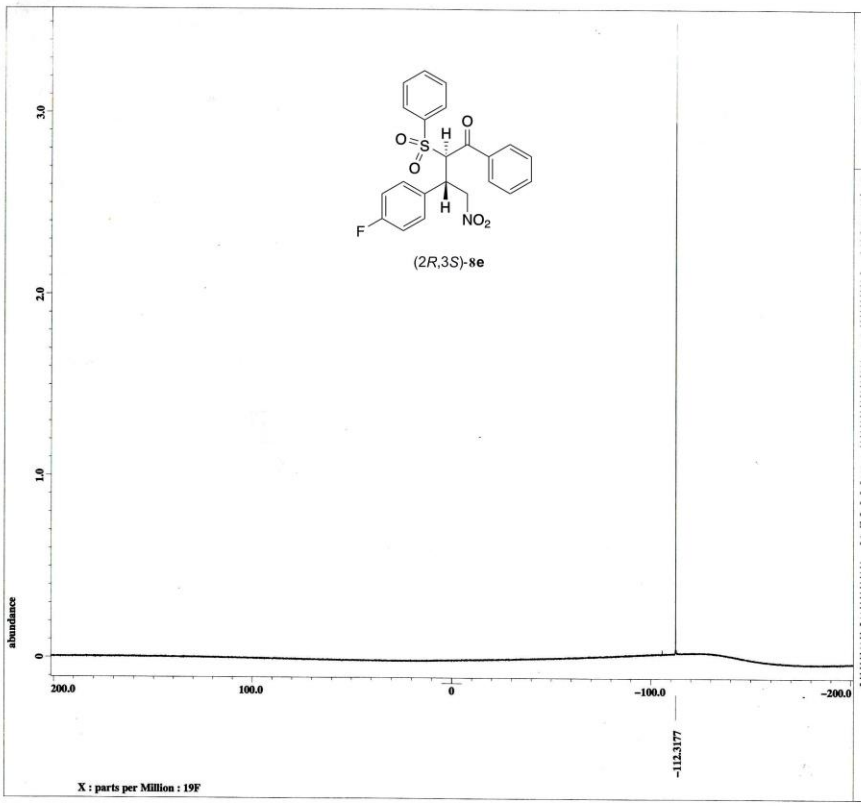

Figure S30. ^{19}F NMR spectra of compound **8e** in $CDCl_3$.

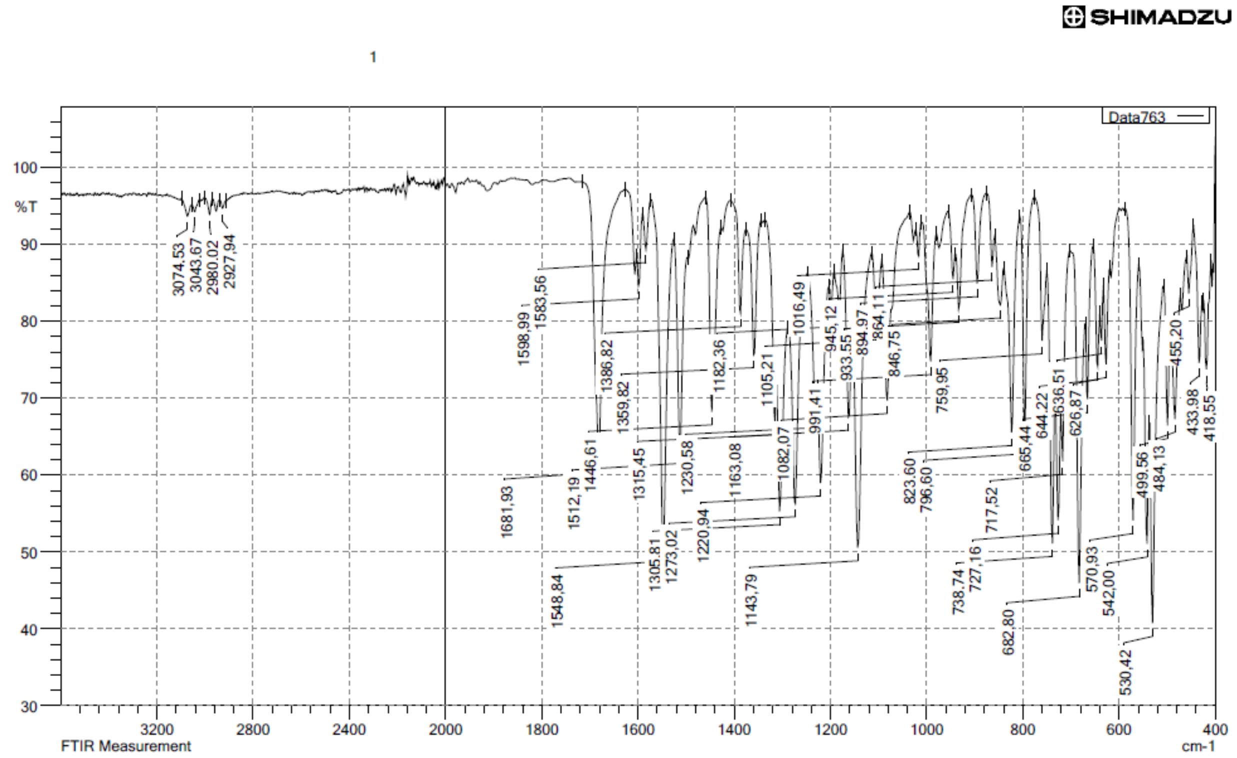

Figure S31. FTIR spectra of compound **8e**.

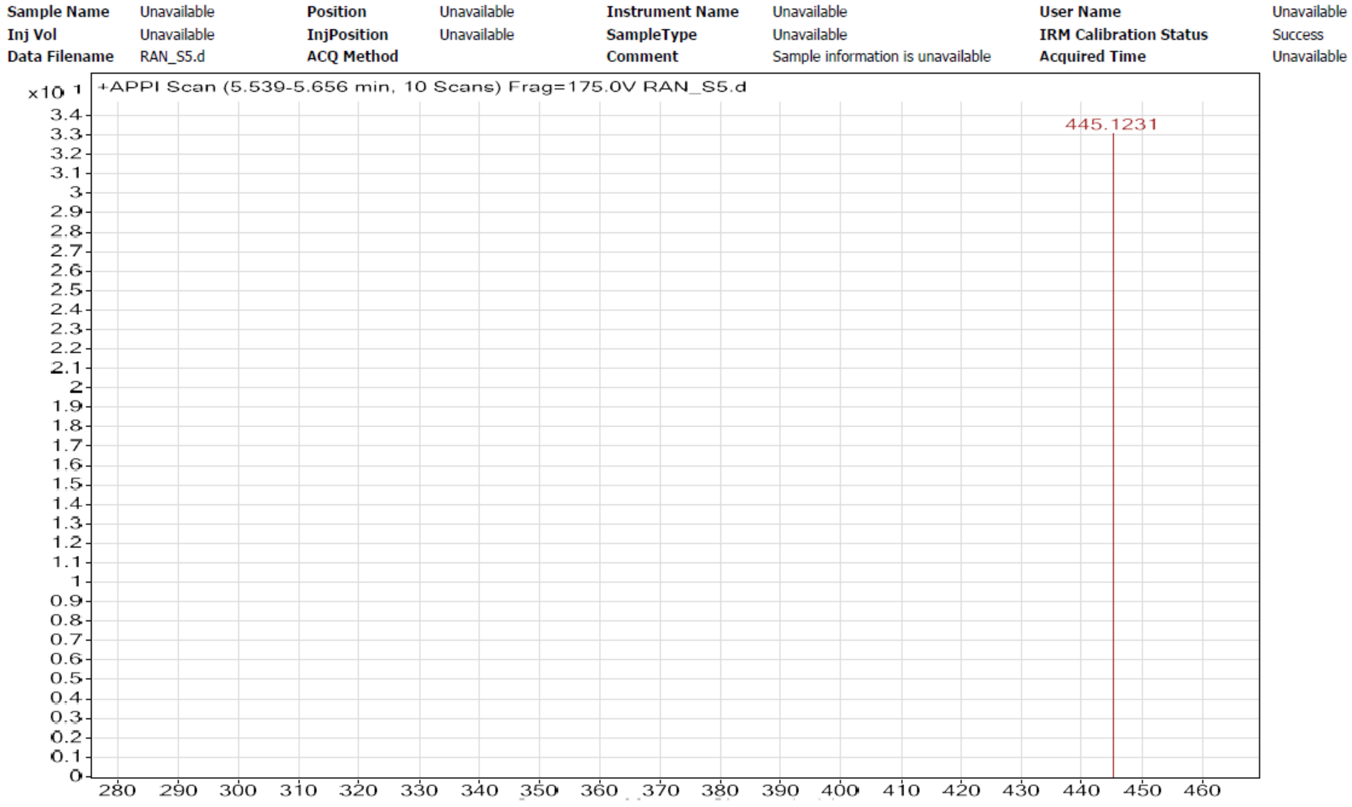

Figure S32. HRMS of compound **8e**.

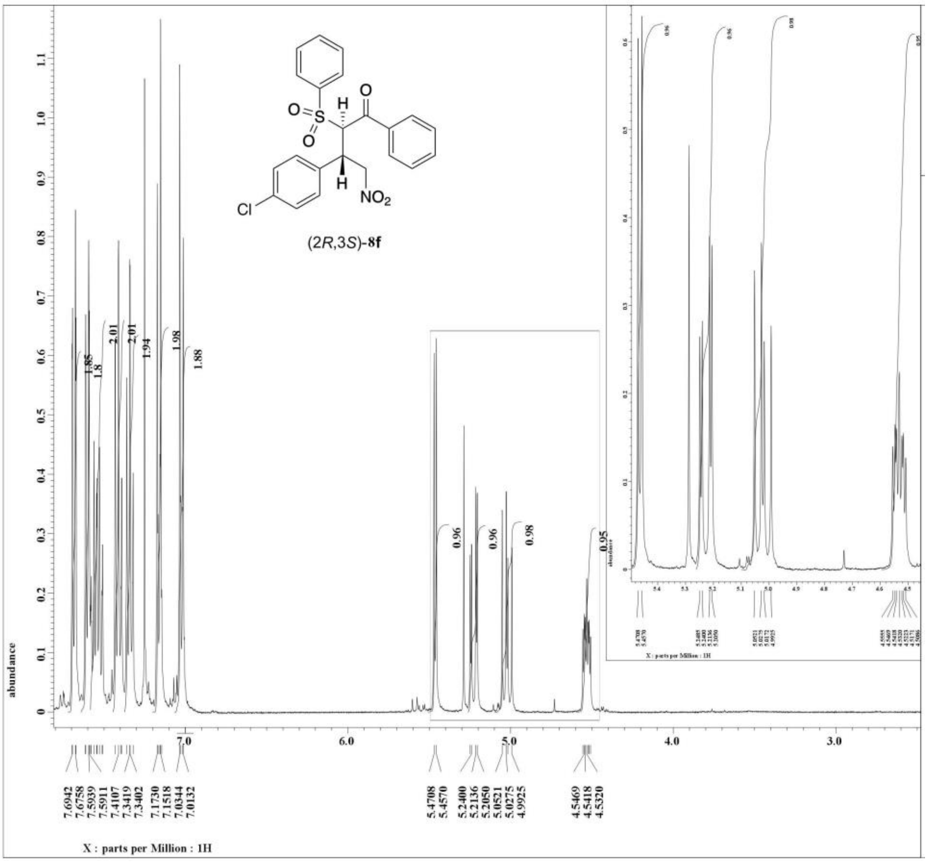

Figure S33. ^{1}H NMR spectra of compound **8f** in $CDCl_3$.

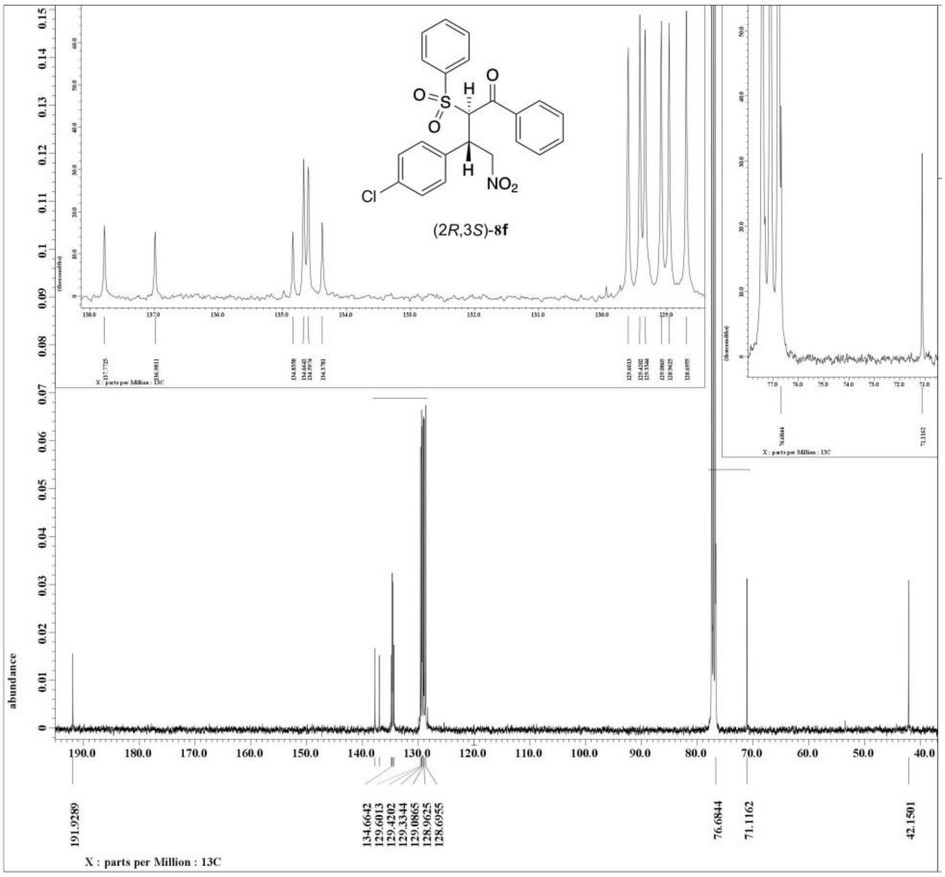

Figure S34. ^{13}C NMR spectra of compound **8f** in $CDCl_3$.

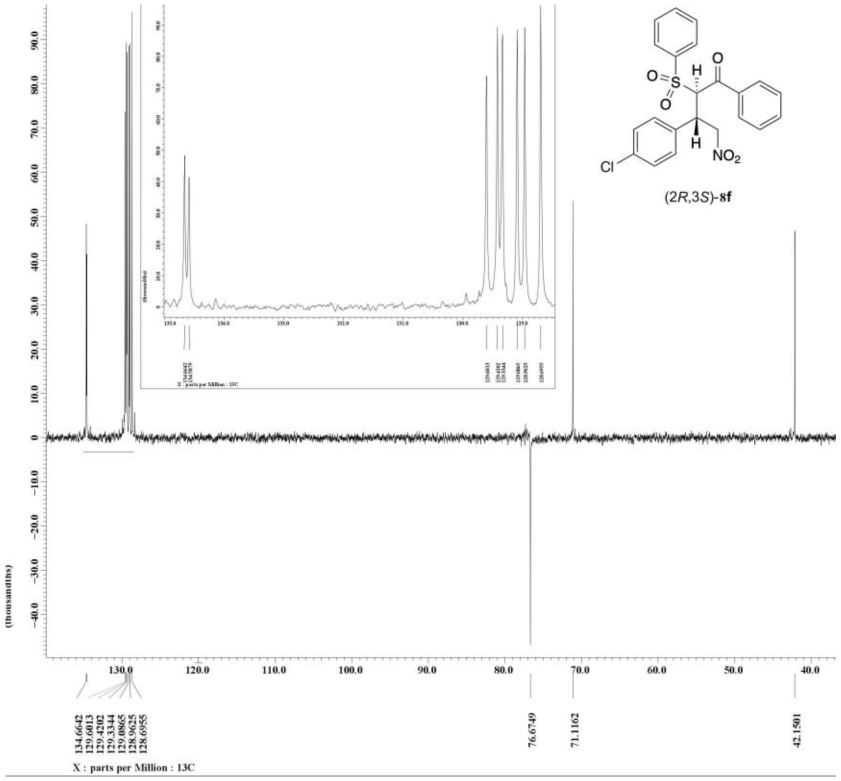

Figure S35. DEPT spectra of compound **8f** in $CDCl_3$.

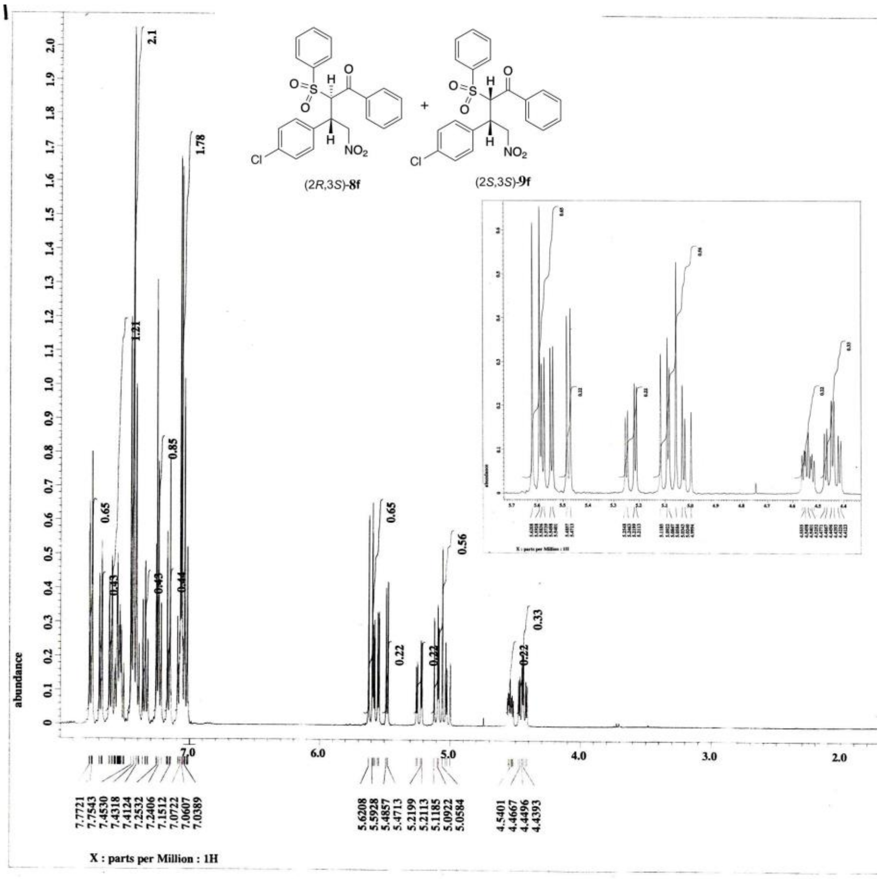

Figure S36. ^{1}H NMR spectra of the mixture of diastereomers **8f** and **9f**in $CDCl_3$.

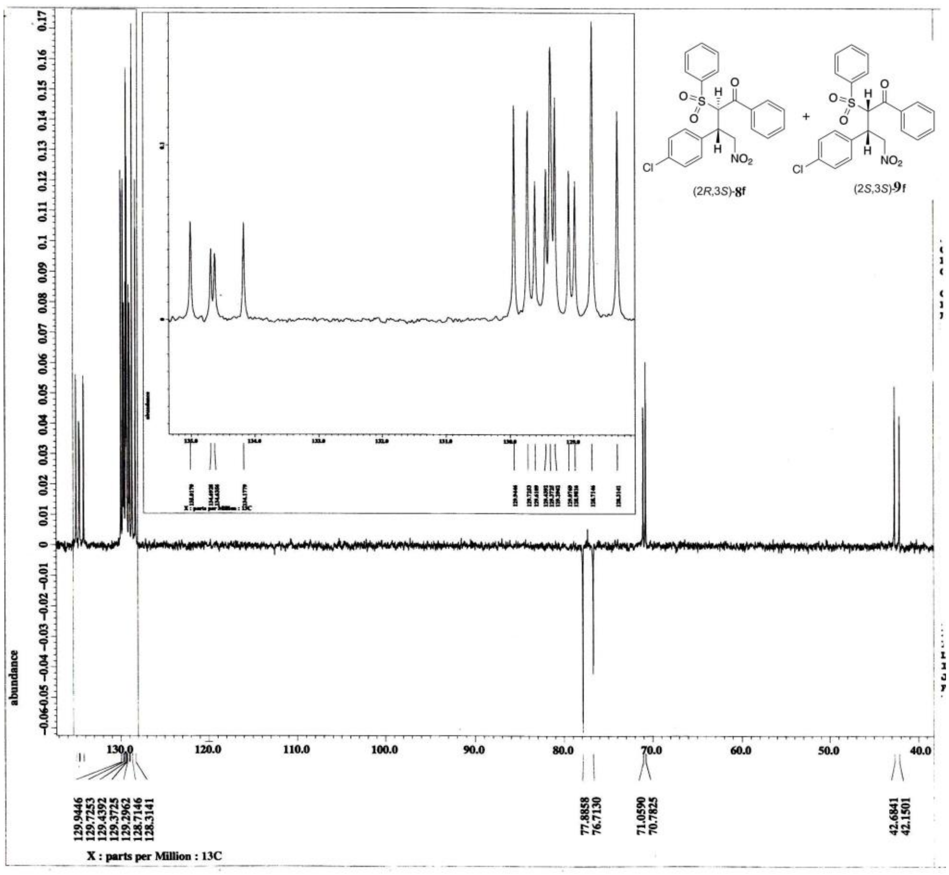

Figure S37. DEPT spectra of the mixture of diastereomers **8f** and **9f** in $CDCl_3$.

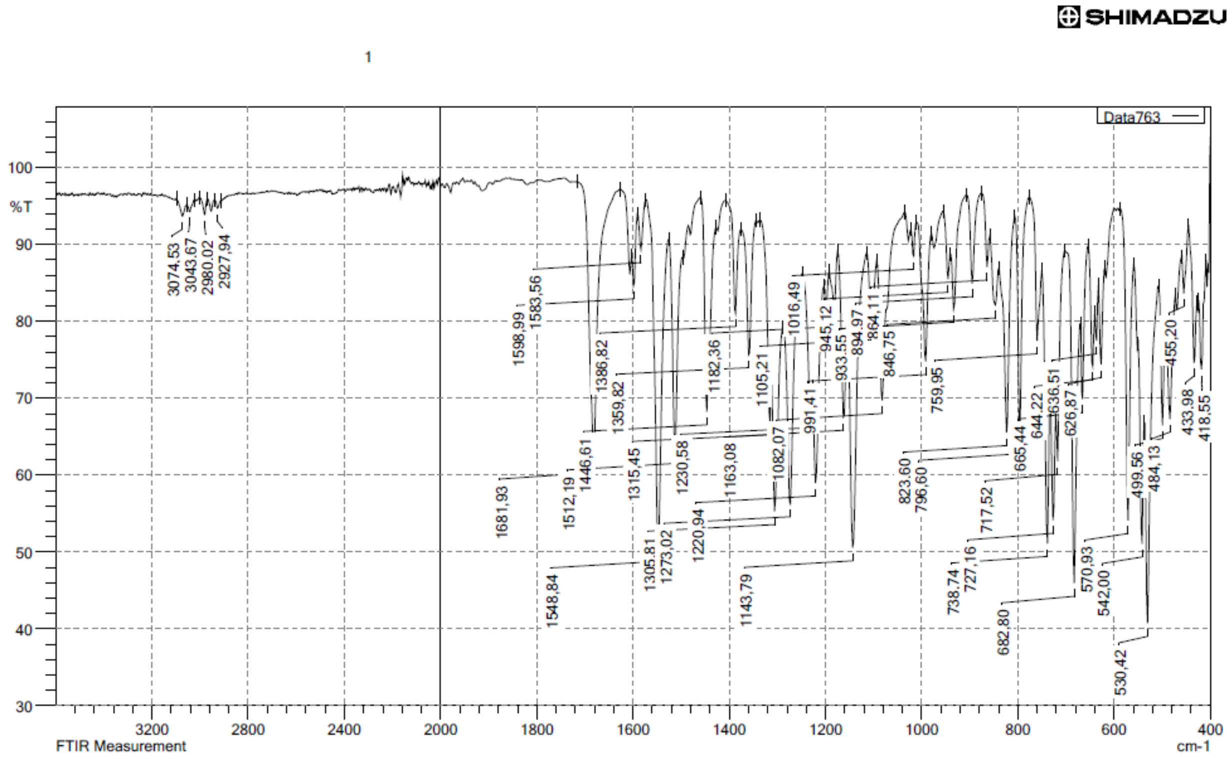

Figure S38. FTIR spectra of compound **8f**.

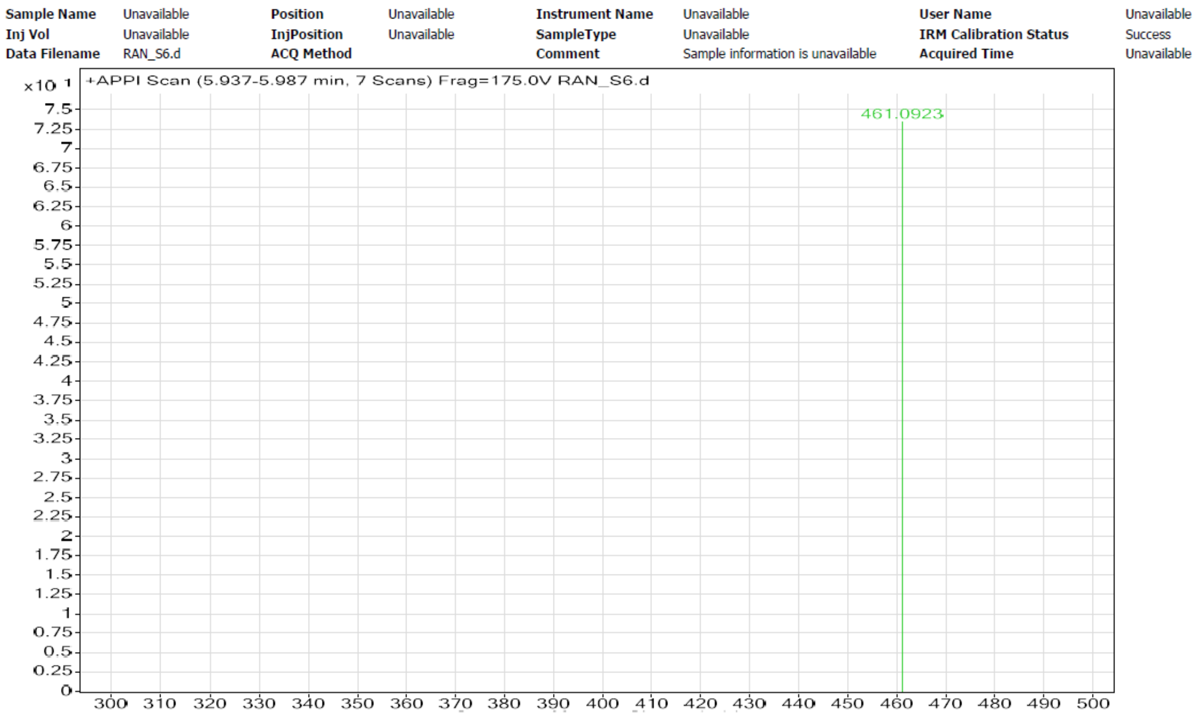

Figure S39. HRMS of compound **8f**.

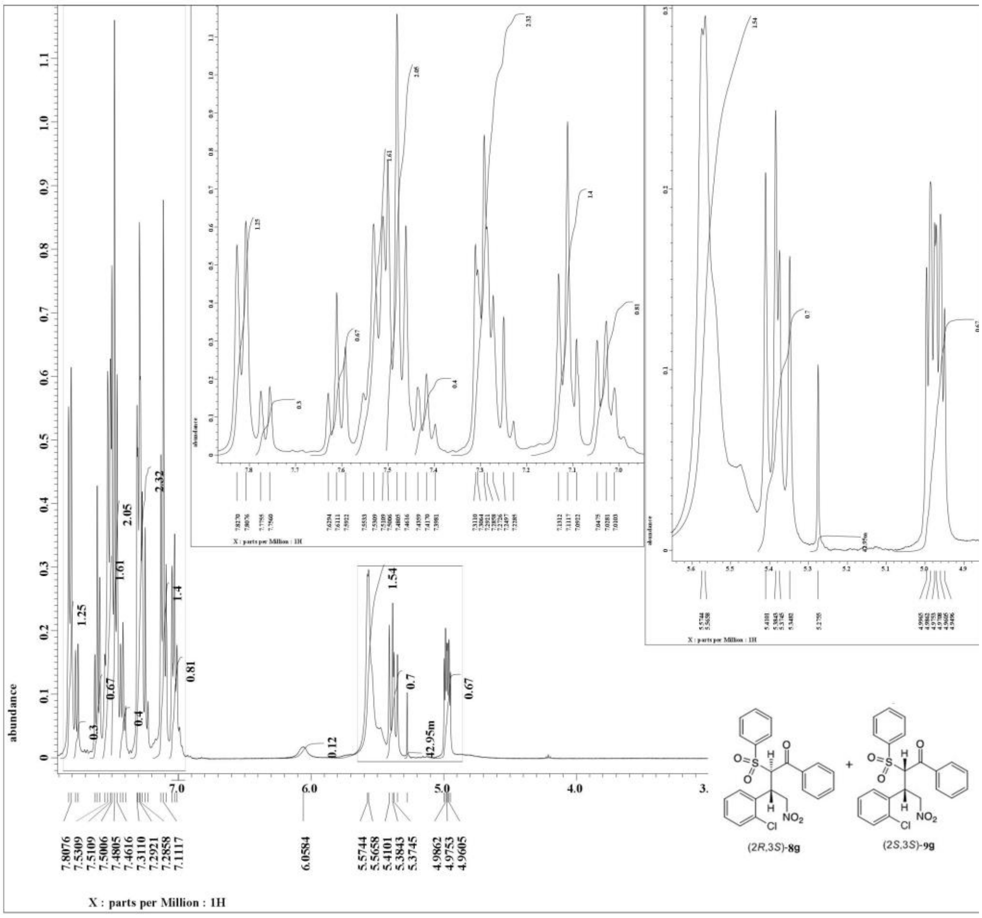

Figure S40. ^{1}H NMR spectra of the mixture of diastereomers **8g** and **9g** in $CDCl_3$.

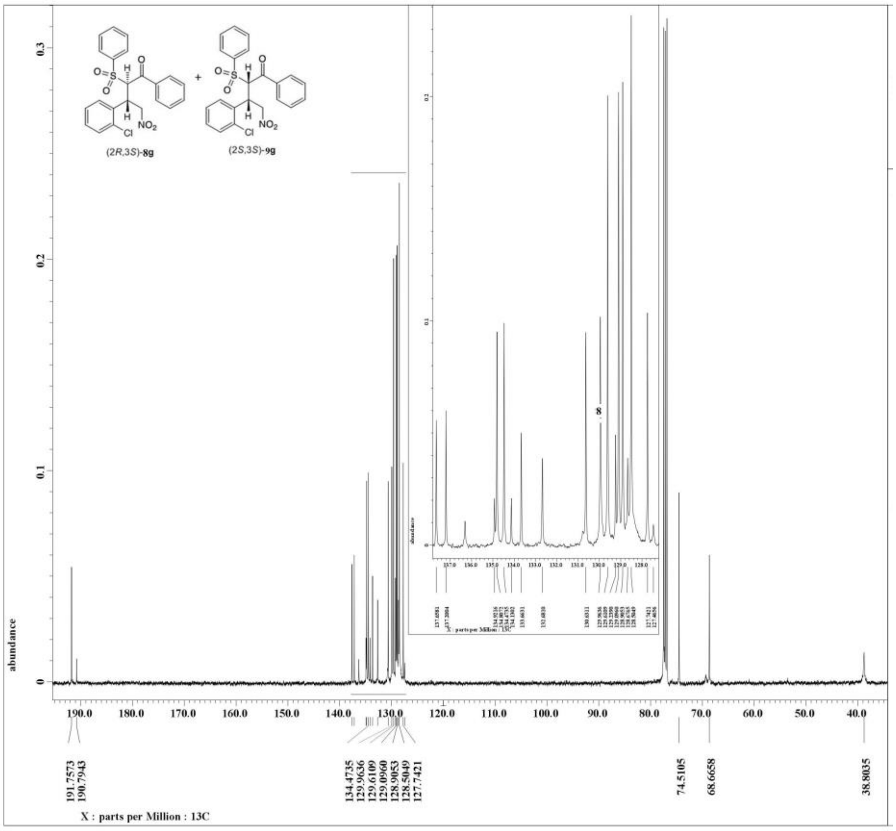

Figure S41. ^{13}C NMR spectra of the mixture of diastereomers **8g** and **9g** in $CDCl_3$.

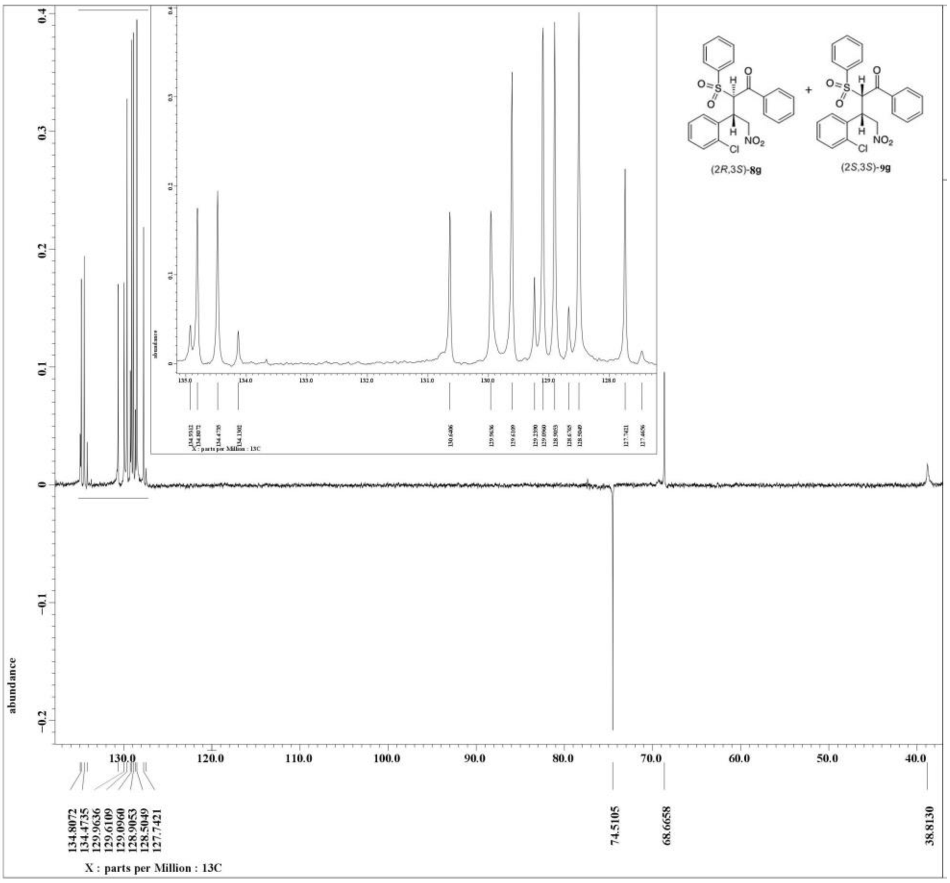

Figure S42. DEPT of the mixture of diastereomers **8g** and **9g** in $CDCl_3$.

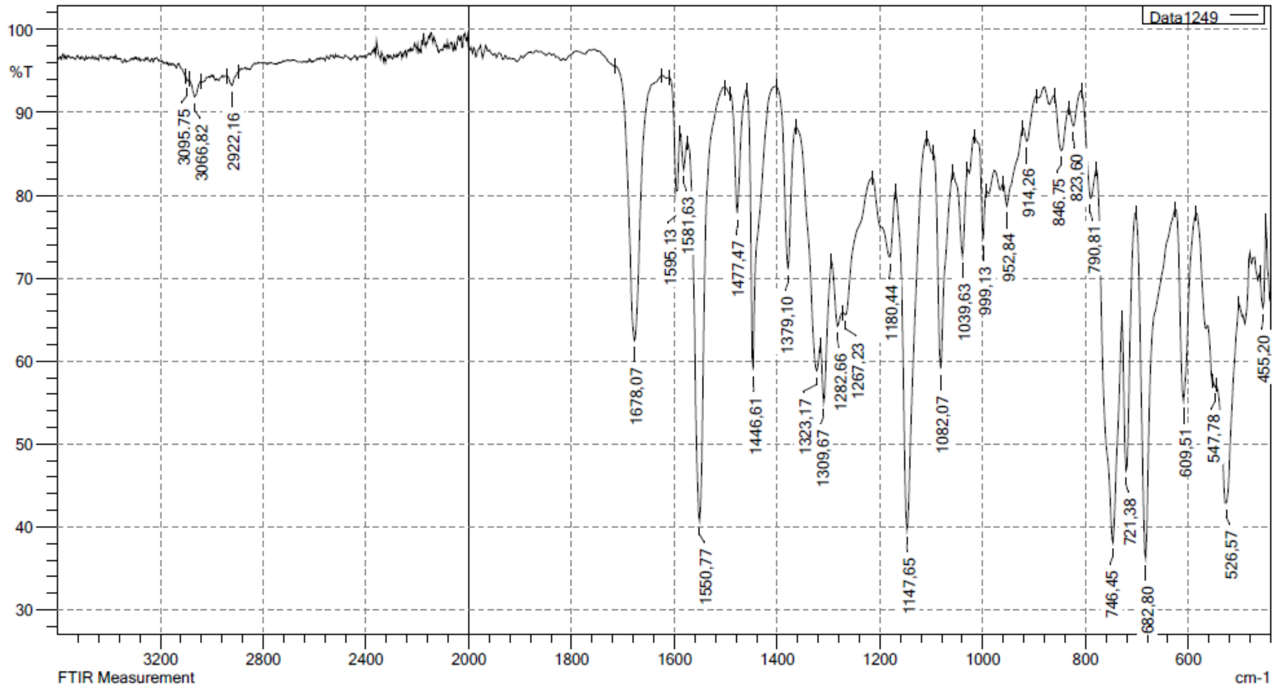

Figure S43. FTIR spectra of compound **8g**.

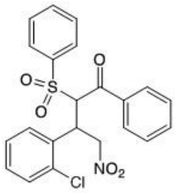

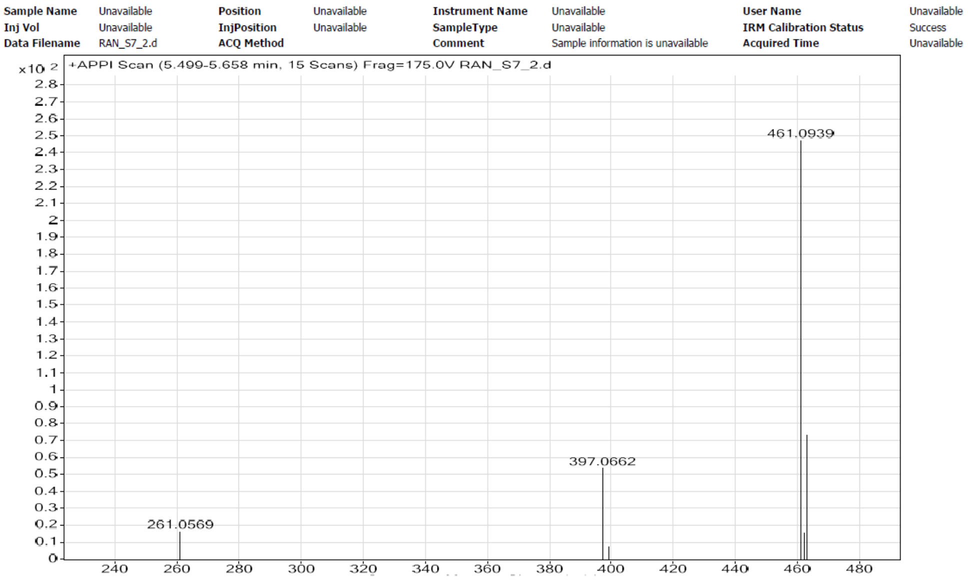

Figure S44. HRMS of compound **8g**.

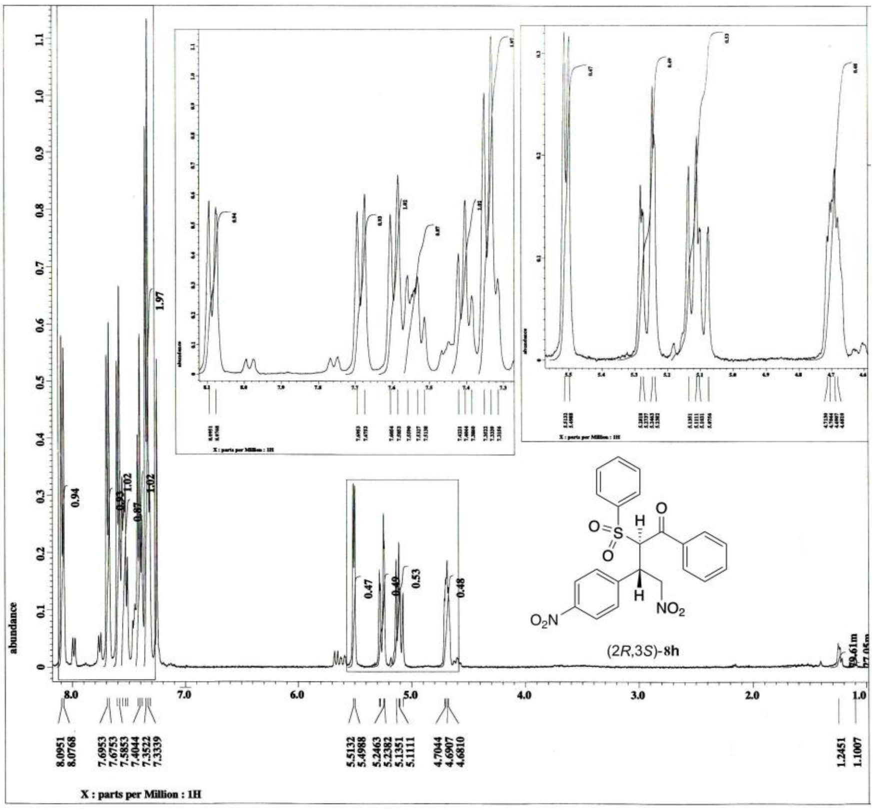

Figure S45. ^{1}H NMR spectra of compound **8h** in $CDCl_3$.

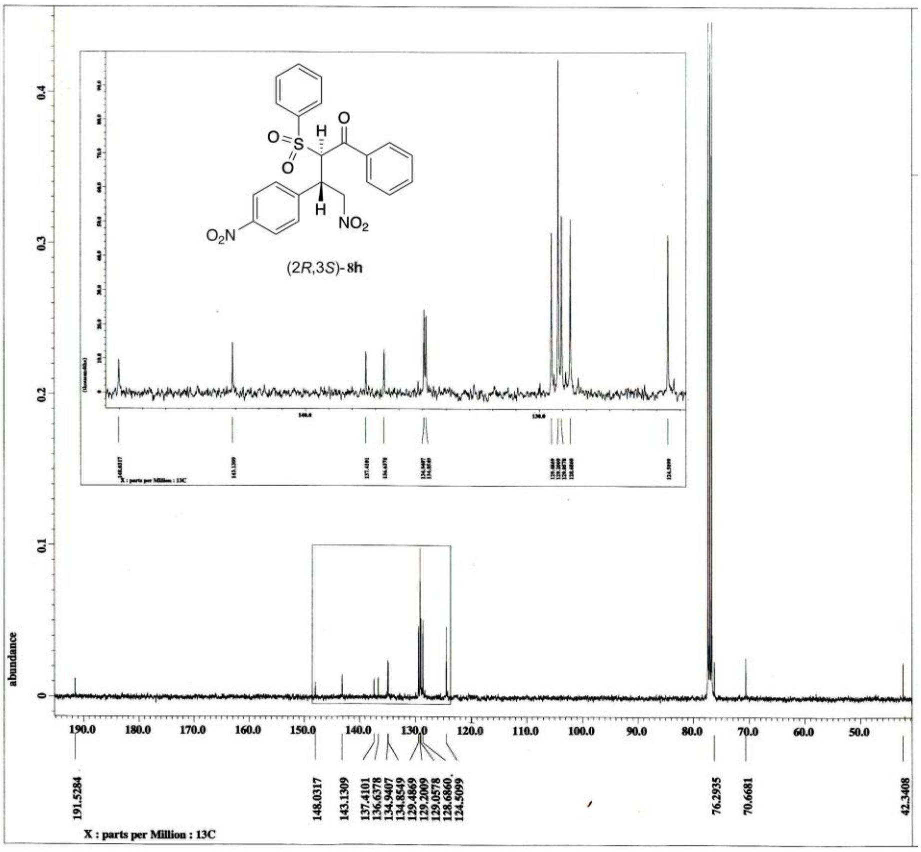

Figure S46. ^{13}C NMR spectra of compound **8h** in $CDCl_3$.

SHIMADZU

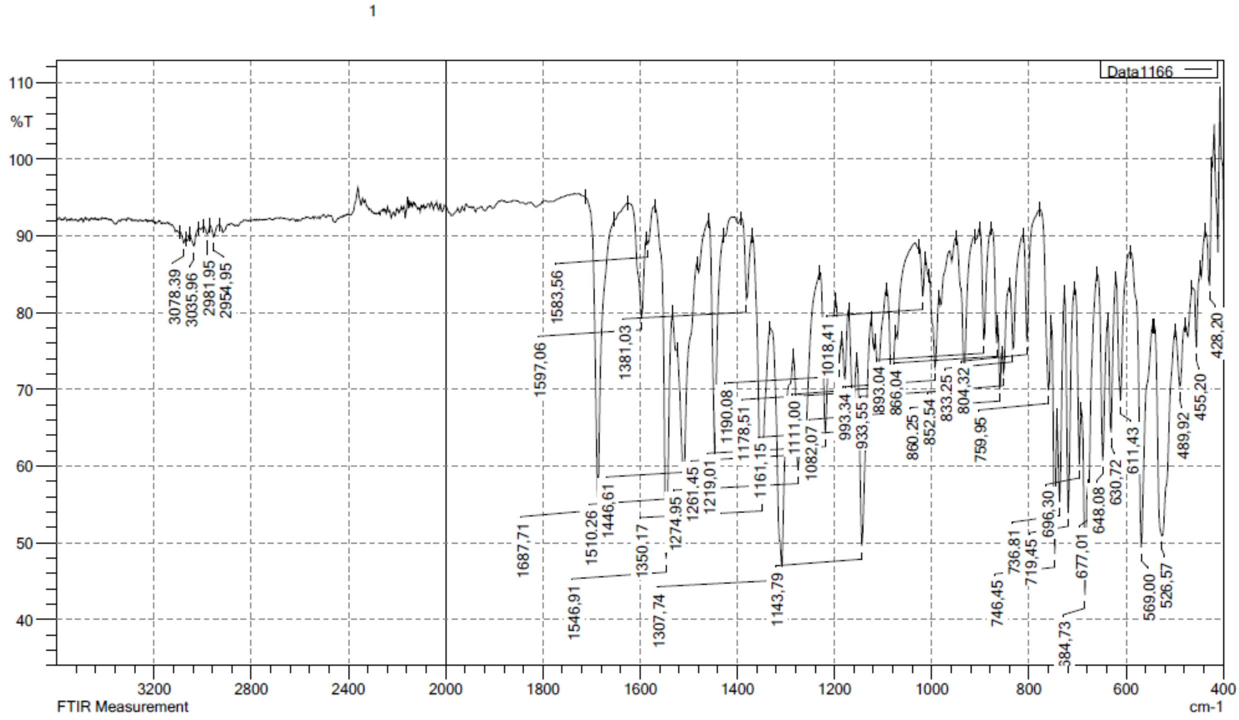

Figure S47. FTIR spectra of compound **8h**.

**Sample Name**	Unavailable	**Position**	Unavailable	**Instrument Name**	Unavailable	**User Name**	Unavailable
**Inj Vol**	Unavailable	**InjPosition**	Unavailable	**SampleType**	Unavailable	**IRM Calibration Status**	Success
**Data Filename**	RAN_S8_NEG.d	**ACQ Method**		**Comment**	Sample information is unavailable	**Acquired Time**	Unavailable

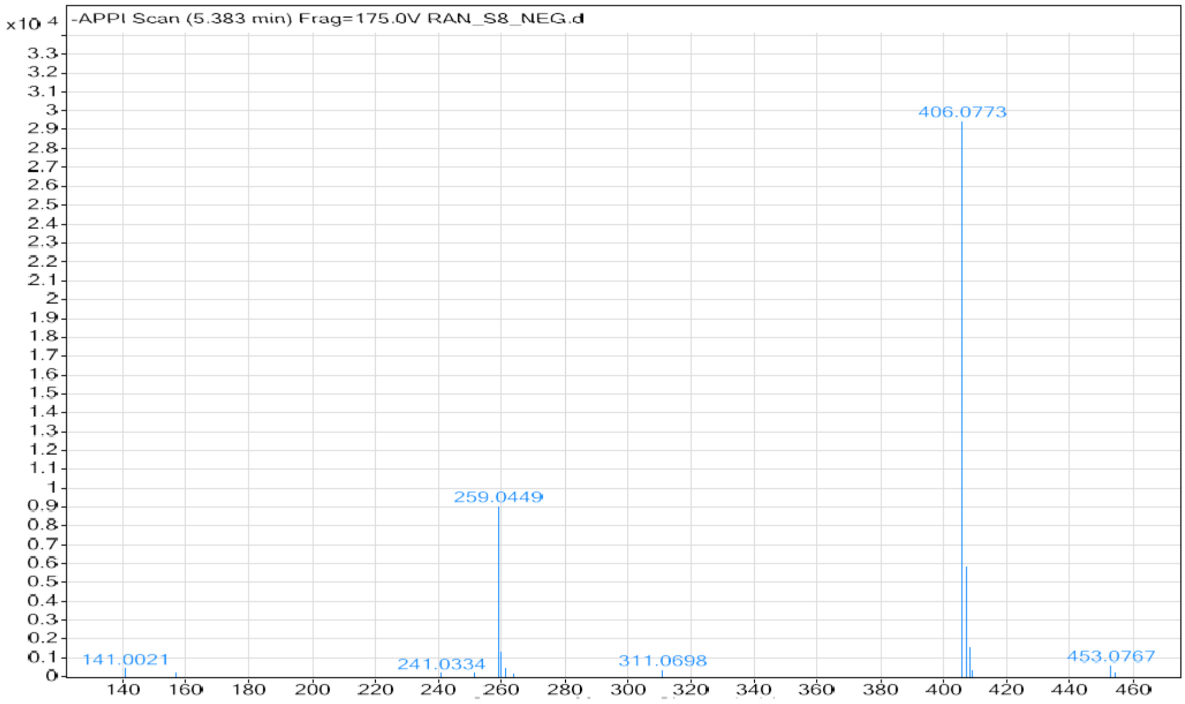

Figure S48. HRMS of compound **8h**.

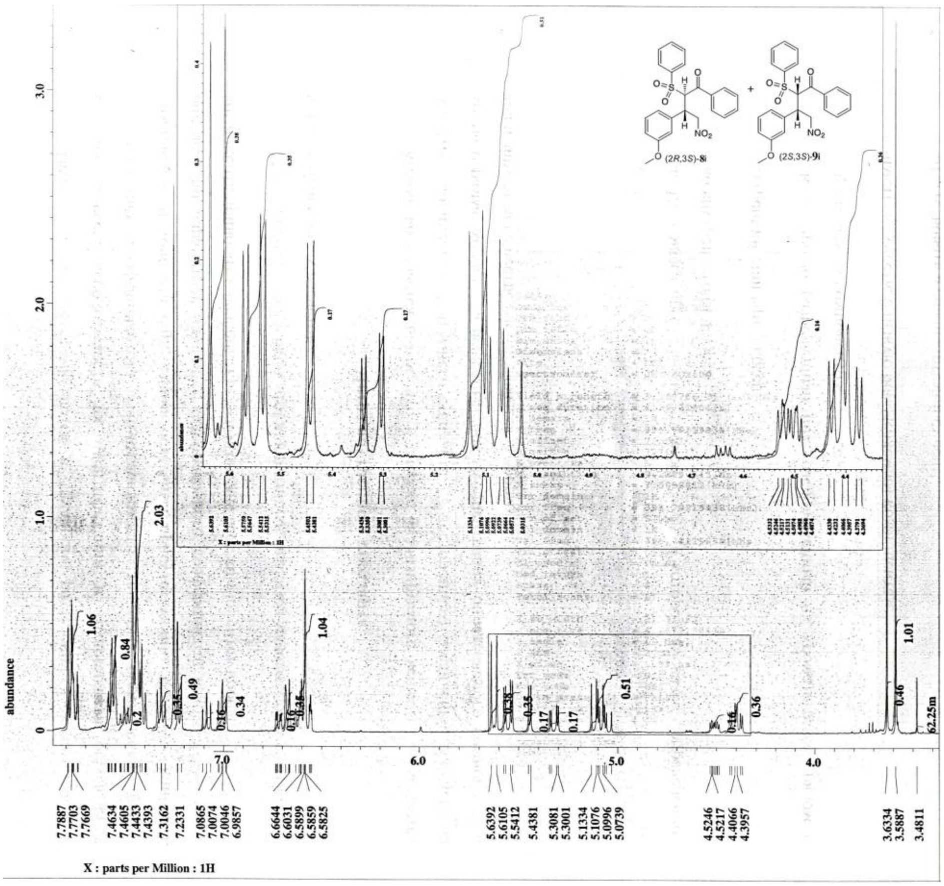

Figure S49. ^{1}H NMR spectra of the mixture of diastereomers **8i** and **9i**.

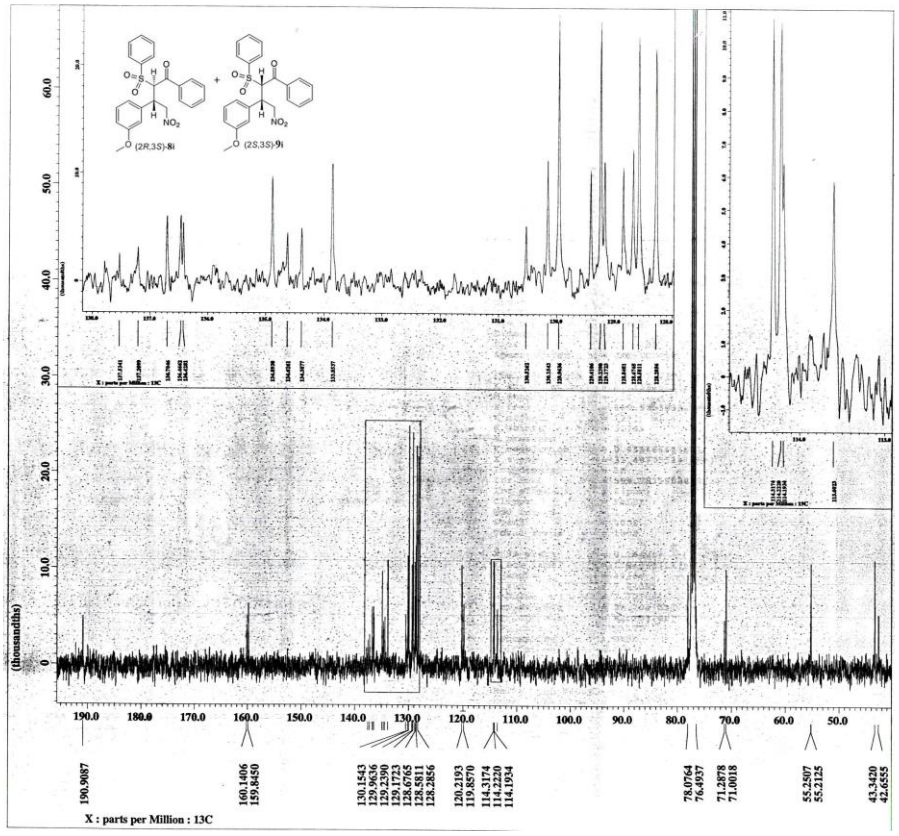

Figure S50. ^{13}C NMR spectra of the mixture of diastereomers **8i** and **9i**.

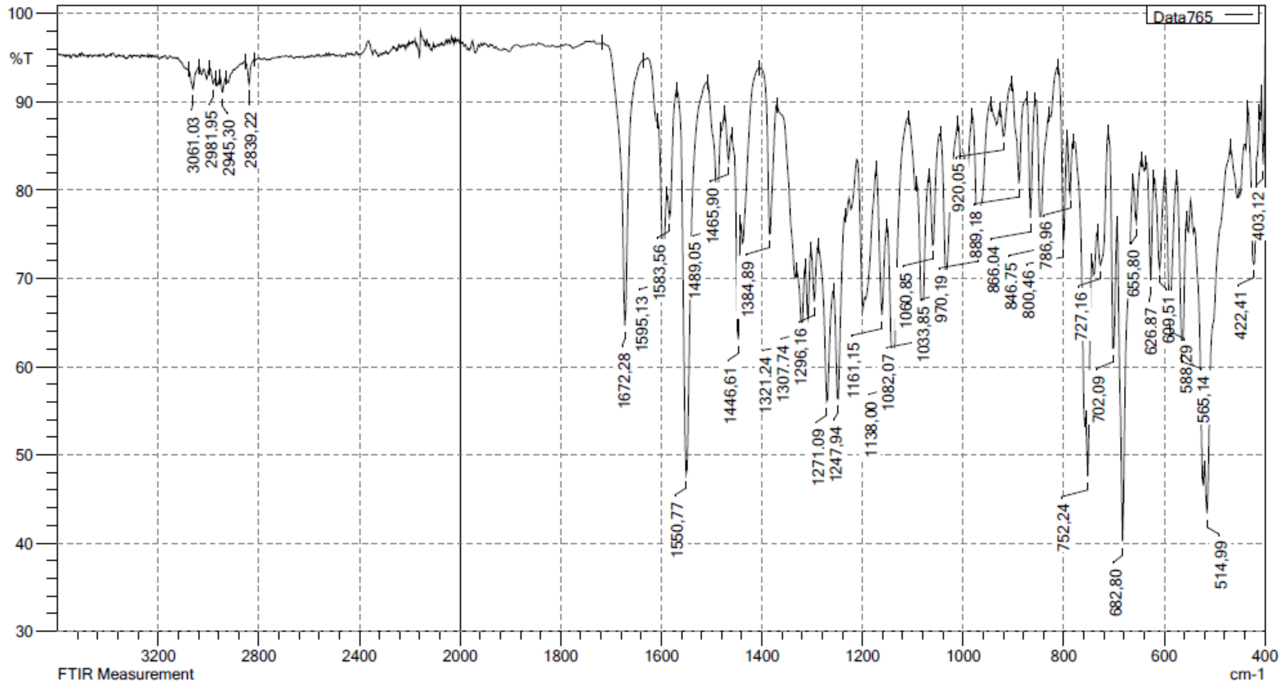

Figure S51. FTIR spectra of the mixture of diastereomers **8i** and **9i**.

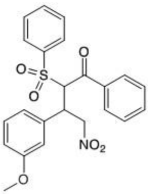

**Sample Name**	Unavailable	**Position**	Unavailable	**Instrument Name**	Unavailable	**User Name**	Unavailable
**Inj Vol**	Unavailable	**InjPosition**	Unavailable	**SampleType**	Unavailable	**IRM Calibration Status**	Success
**Data Filename**	RAN_S9_2.d	**ACQ Method**		**Comment**	Sample information is unavailable	**Acquired Time**	Unavailable

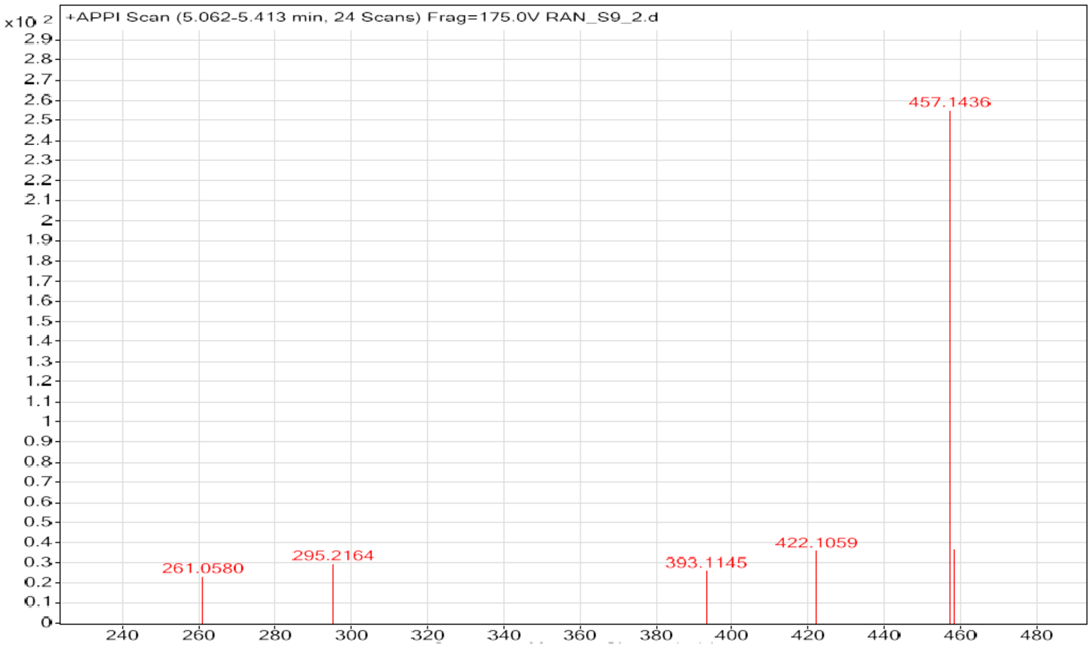

Figure S52. HRMS of compound **8i**.

Empower 2 software

chrom1

SAMPLE INFORMATION

Sample Name:	SO2CHPhCHPh(rac)AD_15%	Acquired By:	System
Sample Type:	Unknown	Sample Set Name:	
Vial:	1	Acq. Method Set:	Hex_Pr_Meth_210
Injection #:	11	Processing Method	hlk
Injection Volume:	10,00 ul	Channel Name:	2487Channel 1
Run Time:	120,0 Minutes	Proc. Chnl. Descr.:	210nm
Date Acquired:	01.06.2017 12:44:24 MSD		
Date Processed:	01.06.2017 13:46:19 MSD		

Auto-Scaled Chromatogram

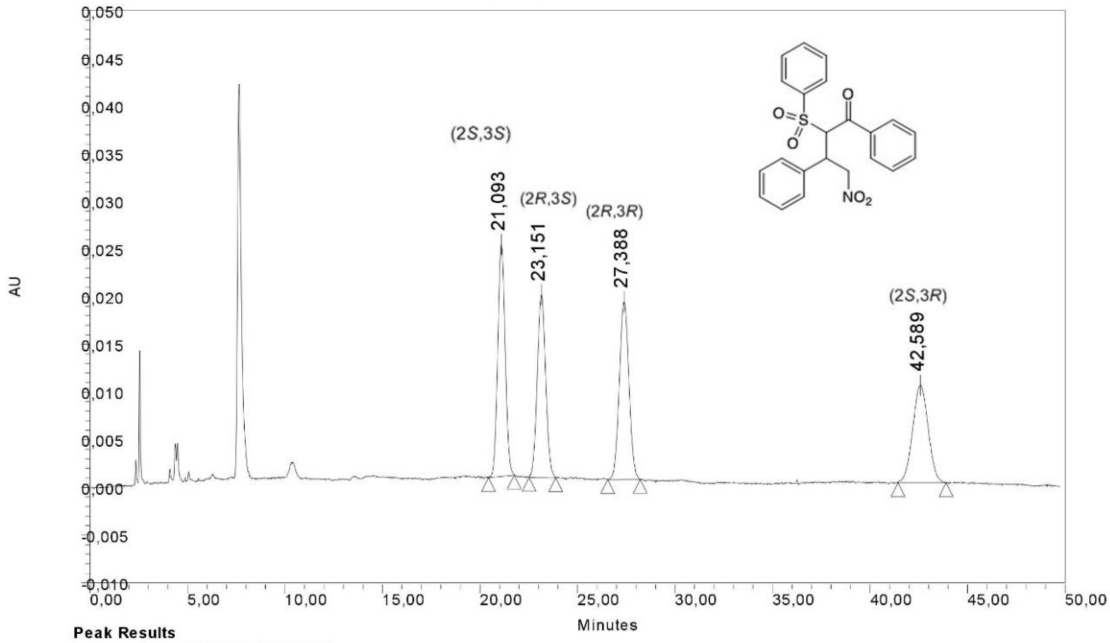

Peak Results

	RT	Height	Area	% Area
1	21,093	24346	643195	26,55
2	23,151	19179	574918	23,73
3	27,388	18647	639206	26,38
4	42,589	10221	565666	23,35

Reported by User: System
Report Method: chrom1
Report Method ID 10097
Page: 1 of 1

Project Name: Default
Date Printed:
01.06.2017
13:50:53 Europe/Moscow

Figure S53. HPLC for racemic **8a**/**9a** (a mixture of diastereomers).

Empower 2 software

chrom1

SAMPLE INFORMATION			
Sample Name:		Acquired By:	System
Sample Type:	Unknown	Sample Set Name:	
Vial:	1	Acq. Method Set:	Hex_Pr_Meth_210
Injection #:	19	Processing Method	I
Injection Volume:	10,00 ul	Channel Name:	2487Channel 1
Run Time:	200,0 Minutes	Proc. Chnl. Descr.:	210nm
Date Acquired:	05.10.2017 14:55:58 MSD		
Date Processed:	05.10.2017 15:47:46 MSD		

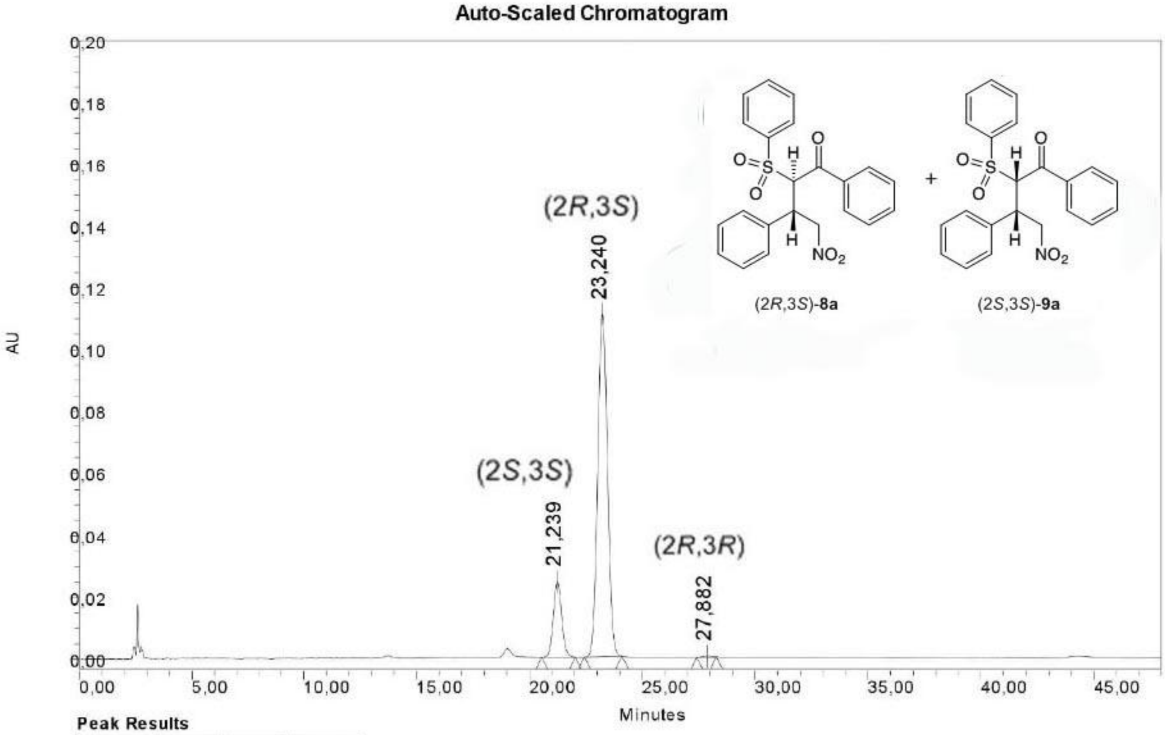

Peak Results

	RT	Height	Area	% Area
1	21,239	24466	645750	16,32
2	23,240	110600	3298281	83,38
3	27,882	404	11775	0,30

Reported by User: System
Report Method: chrom1
Report Method ID 10490
Page: 1 of 1

Project Name: Default
Date Printed:
05.10.2017
15:53:51 Europe/Moscow

Figure S54. HPLC for (2*R*,3*S*)-**8a** and (2*S*,3*S*)-**9a**.

chrom1

SAMPLE INFORMATION

Sample Name:	sylfon-Cl-p_(rac)AD_30%1.2ml	Acquired By:	System
Sample Type:	Unknown	Sample Set Name:	
Vial:	1	Acq. Method Set:	Hex_Pr_Meth_210
Injection #:	31	Processing Method	gyjuik
Injection Volume:	10,00 ul	Channel Name:	2487Channel 1
Run Time:	250,0 Minutes	Proc. Chnl. Descr.:	210nm
Date Acquired:	29.11.2017 13:54:48 MSK		
Date Processed:	29.11.2017 14:20:29 MSK		

Auto-Scaled Chromatogram

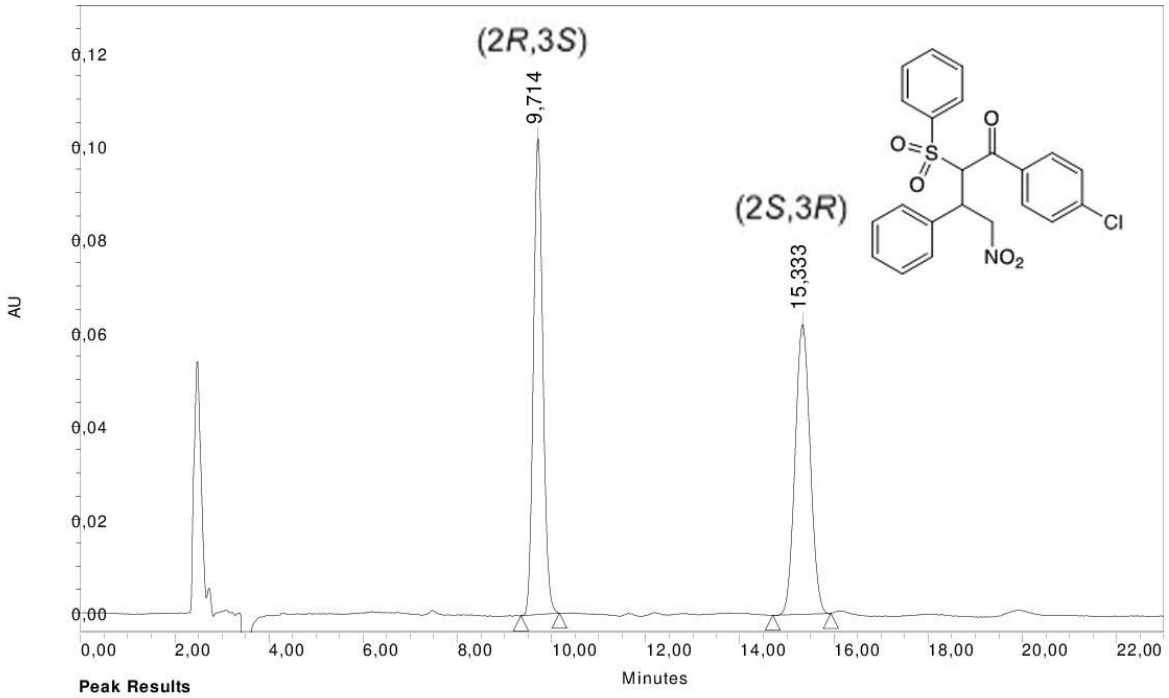

Peak Results

	RT	Height	Area	% Area
1	9,714	101985	1417882	50,63
2	15,333	62047	1382669	49,37

Reported by User: System
Report Method: chrom1
Report Method ID 11057
Page: 1 of 1

Project Name: Default
Date Printed:
29.11.2017
14:22:19 Europe/Moscow

Figure S55. HPLC for racemic **8b**.

Empower 2 software

chrom1

SAMPLE INFORMATION

Sample Name:	sylfon-Cl-p_(ind)AD_30%1.2ml	Acquired By:	System
Sample Type:	Unknown	Sample Set Name:	
Vial:	1	Acq. Method Set:	Hex_Pr_Meth_210
Injection #:	32	Processing Method	hyyh
Injection Volume:	10,00 ul	Channel Name:	2487Channel 1
Run Time:	250,0 Minutes	Proc. Chnl. Descr.:	210nm
Date Acquired:	29.11.2017 14:19:11 MSK		
Date Processed:	29.11.2017 14:55:07 MSK		

Auto-Scaled Chromatogram

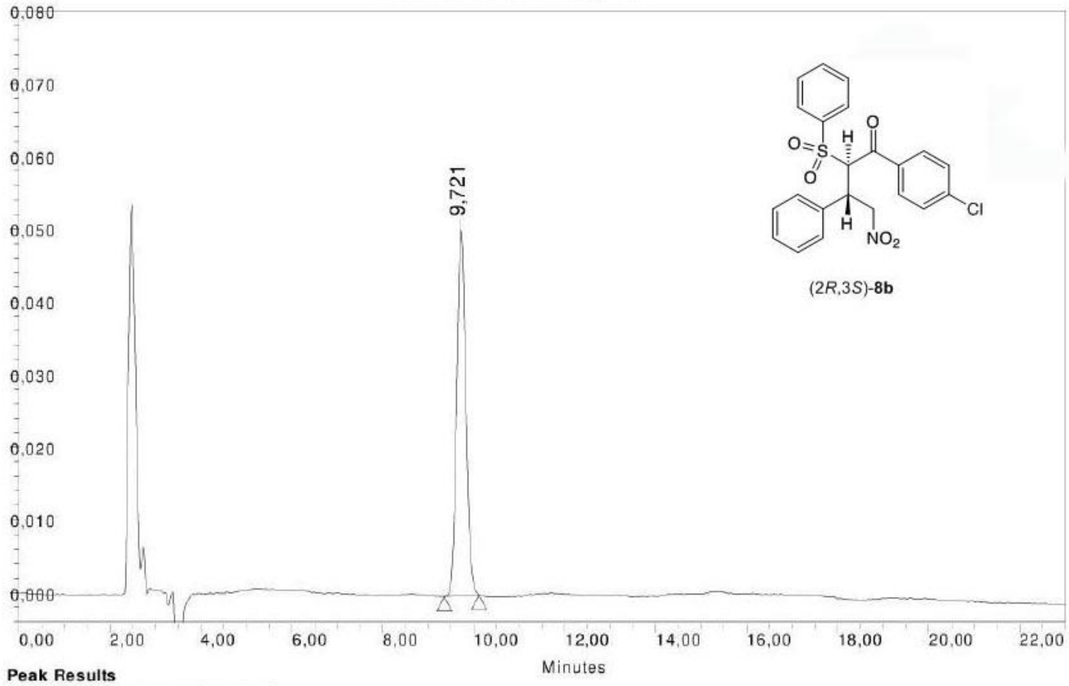

Peak Results

	RT	Height	Area	% Area
1	9,721	50282	699396	100,00

Reported by User: System
Report Method: chrom1
Report Method ID 11067
Page: 1 of 1

Project Name: Default
Date Printed:
29.11.2017
14:56:59 Europe/Moscow

Figure S56. HPLC for (2*R*,3*S*)-**8b**.

Empower 2 software

chrom1

SAMPLE INFORMATION

Sample Name:	sylf-OMe-o_(rac)AD_25%1.2ml	Acquired By:	System
Sample Type:	Unknown	Sample Set Name:	
Vial:	1	Acq. Method Set:	Hex_Pr_Meth_210
Injection #:	12	Processing Method	jhkil
Injection Volume:	10,00 ul	Channel Name:	2487Channel 1
Run Time:	250,0 Minutes	Proc. Chnl. Descr.:	210nm
Date Acquired:	16.11.2017 16:11:25 MSK		
Date Processed:	16.11.2017 16:54:10 MSK		

Auto-Scaled Chromatogram

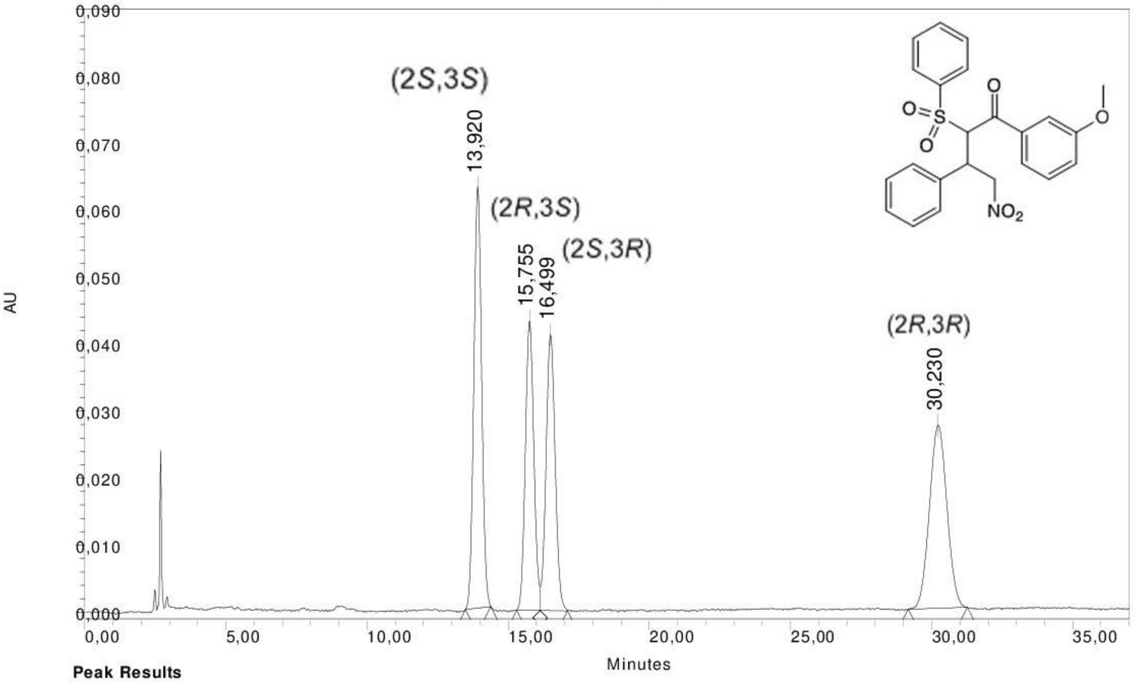

Peak Results

	RT	Height	Area	% Area
1	13,920	62871	1212658	28,73
2	15,755	43195	908507	21,52
3	16,499	41232	923718	21,88
4	30,230	27294	1175963	27,86

Reported by User: System
Report Method: chrom1
Report Method ID 10911
Page: 1 of 1

Project Name: Default
Date Printed:
16.11.2017
16:55:33 Europe/Moscow

Figure S57. HPLC for racemic **8c**/**9c** (a mixture of diastereomers).

Empower 2 software

chrom1

SAMPLE INFORMATION

Sample Name:	sylf-OMe-o_(ind)AD_25%1.2ml	Acquired By:	System
Sample Type:	Unknown	Sample Set Name:	
Vial:	1	Acq. Method Set:	Hex_Pr_Meth_210
Injection #:	14	Processing Method	jghj
Injection Volume:	10,00 ul	Channel Name:	2487Channel 1
Run Time:	250,0 Minutes	Proc. Chnl. Descr.:	210nm
Date Acquired:	16.11.2017 17:36:30 MSK		
Date Processed:	16.11.2017 18:19:25 MSK		

Auto-Scaled Chromatogram

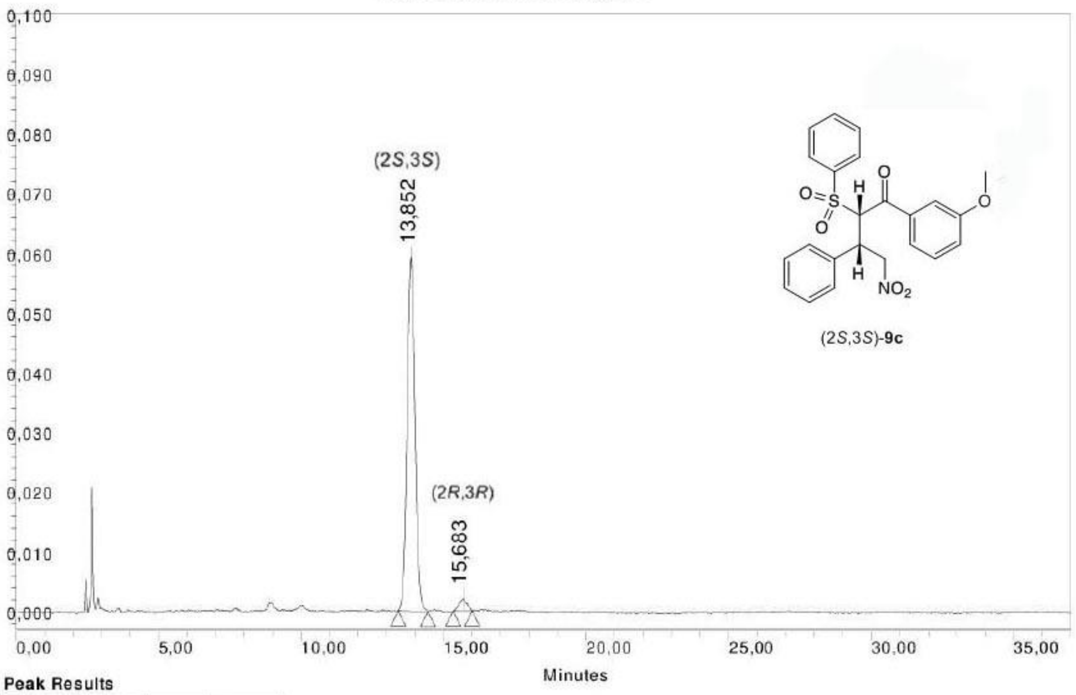

Peak Results

	RT	Height	Area	% Area
1	13,852	59131	1143589	96,66
2	15,683	2054	39463	3,34

Reported by User: System
Report Method: chrom1
Report Method ID 10927
Page: 1 of 1

Project Name: Default
Date Printed:
16.11.2017
18:19:51 Europe/Moscow

Figure S58. HPLC for (2*S*,3*S*)-**9c**.

chrom1

S A M P L E   I N F O R M A T I O N

Sample Name:	SO2_sylfon-Ad_(rac)AD_15%	Acquired By:	System
Sample Type:	Unknown	Sample Set Name:	
Vial:	1	Acq. Method Set:	Hex_Pr_Meth_210
Injection #:	11	Processing Method	ggg
Injection Volume:	10,00 ul	Channel Name:	2487Channel 1
Run Time:	200,0 Minutes	Proc. Chnl. Descr.:	210nm
Date Acquired:	19.10.2017 18:36:30 MSD		
Date Processed:	19.10.2017 19:05:00 MSD		

Auto-Scaled Chromatogram

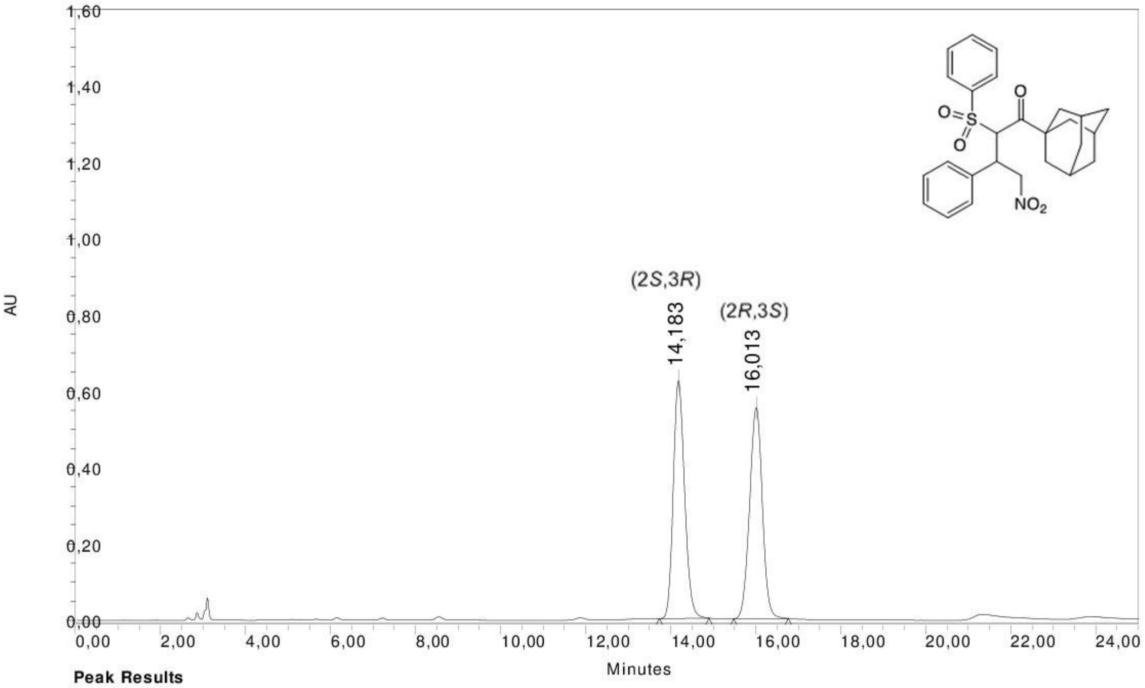

Peak Results

	RT	Height	Area	% Area
1	14,183	622109	11528575	50,03
2	16,013	553066	11516145	49,97

Reported by User: System
Report Method: chrom1
Report Method ID 10703
Page: 1 of 1

Project Name: Default
Date Printed:
19.10.2017
19:05:54 Europe/Moscow

Figure S59. HPLC for racemic **8d**.

Empower 2 software

chrom1

SAMPLE INFORMATION

Sample Name:	SO2_sylfon-Ad_(ind)AD_15%	Acquired By:	System
Sample Type:	Unknown	Sample Set Name:	
Vial:	1	Acq. Method Set:	Hex_Pr_Meth_210
Injection #:	3	Processing Method	nbv
Injection Volume:	10,00 ul	Channel Name:	2487Channel 1
Run Time:	200,0 Minutes	Proc. Chnl. Descr.:	210nm
Date Acquired:	19.10.2017 13:43:20 MSD		
Date Processed:	19.10.2017 14:42:31 MSD		

Auto-Scaled Chromatogram

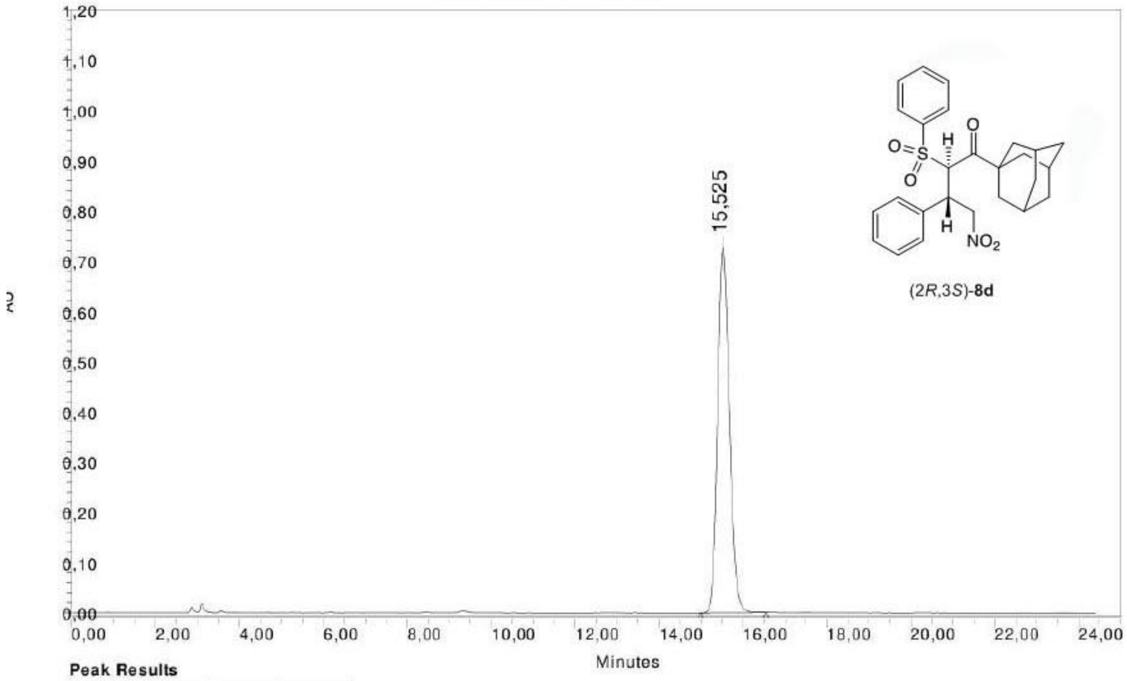

Peak Results

	RT	Height	Area	% Area
1	15,525	727160	14224229	100,00

Reported by User: System
Report Method: chrom1
Report Method ID 10684
Page: 1 of 1

Project Name: Default
Date Printed:
19.10.2017
14:44:25 Europe/Moscow

Figure S60. HPLC for (2*R*,3*S*)-**8d**.

Empower2 software

chrom2

S A M P L E   I N F O R M A T I O N

Sample Name:	SO2_alkene-p-F_(rac)AD_15%	Acquired By:	System
Sample Type:	Unknown	Sample Set Name:	
Vial:	1	Acq. Method Set:	Hex_Pr_Meth_210
Injection #:	9	Processing Method	lkjh
Injection Volume:	10,00 ul	Channel Name:	2487Channel 2
Run Time:	200,0 Minutes	Proc. Chnl. Descr.:	230nm
Date Acquired:	19.10.2017 17:08:44 MSD		
Date Processed:	19.10.2017 17:53:06 MSD		

Auto-Scaled Chromatogram

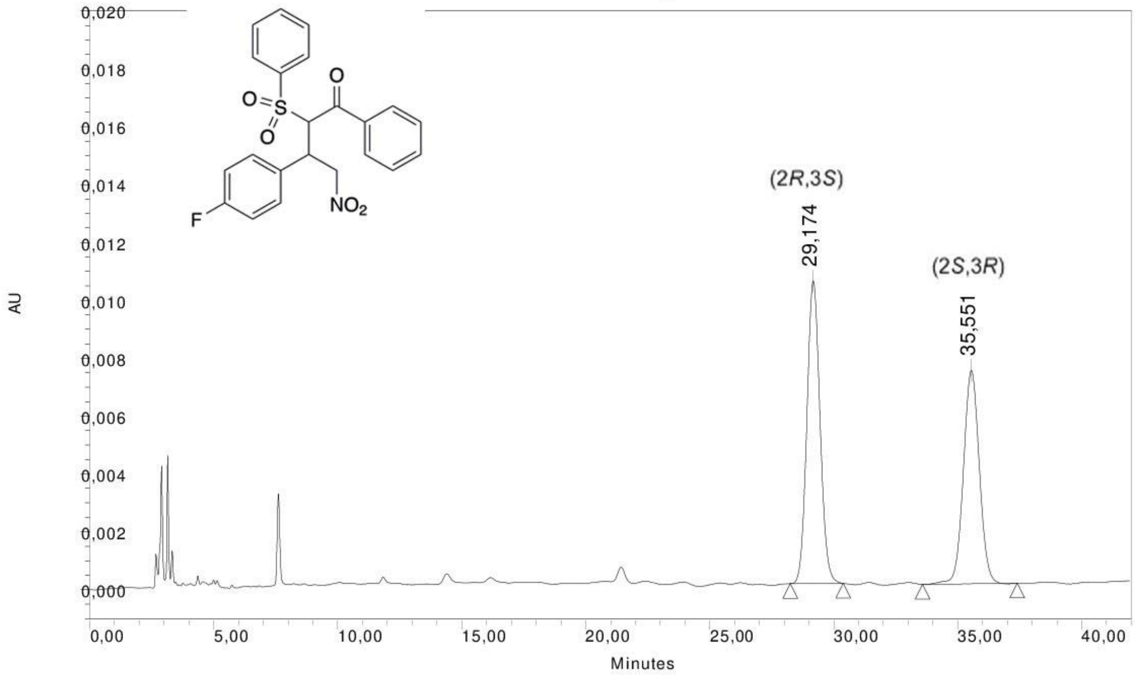

Peak Results

	RT	Height	Area	% Area
1	29,174	10457	384416	53,82
2	35,551	7374	329834	46,18

Reported by User: System
Report Method: chrom2
Report Method ID 10724
Page: 1 of 1

Project Name: Default
Date Printed:
19.10.2017
17:54:53 Europe/Moscow

Figure S61. HPLC for racemic **8e**.

Empower2 software

chrom2

SAMPLE INFORMATION

Sample Name:	SO2_alkene-p-F_(ind)AD_15%	Acquired By:	System
Sample Type:	Unknown	Sample Set Name:	
Vial:	1	Acq. Method Set:	Hex_Pr_Meth_210
Injection #:	8	Processing Method	hhrd
Injection Volume:	10,00 ul	Channel Name:	2487Channel 1
Run Time:	200,0 Minutes	Proc. Chnl. Descr.:	210nm
Date Acquired:	19.10.2017 16:26:12 MSD		
Date Processed:	19.10.2017 17:56:43 MSD		

Auto-Scaled Chromatogram

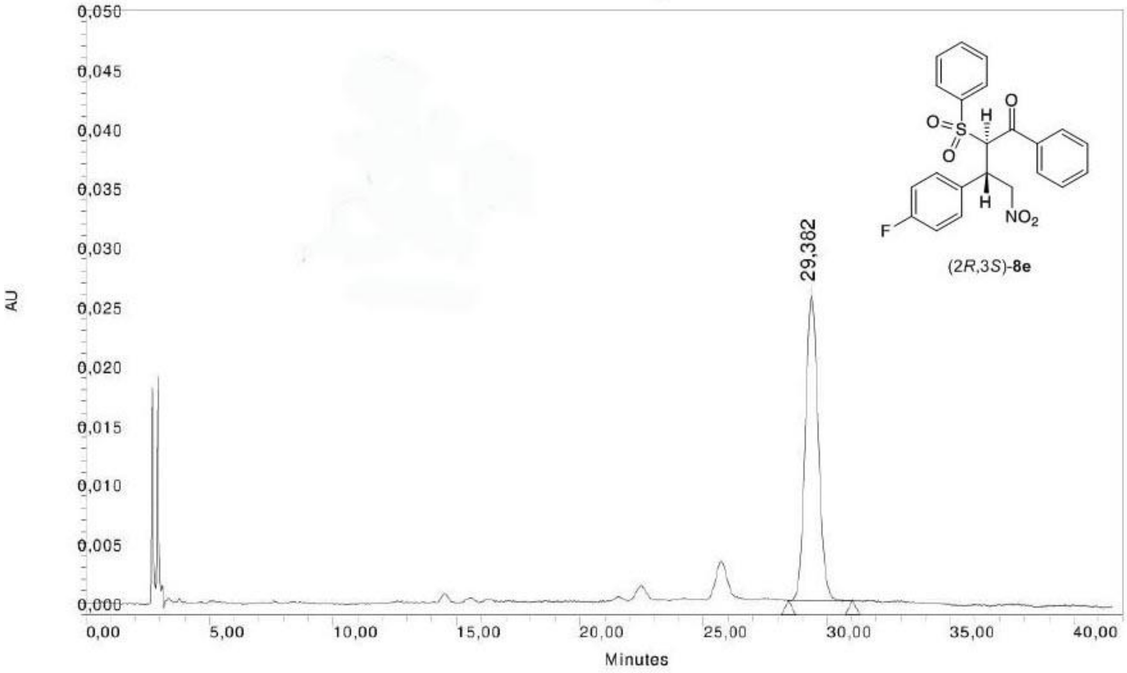

Peak Results

	RT	Height	Area	% Area
1	29,382	25662	940056	100,00

Reported by User: System
Report Method: chrom2
Report Method ID 10729
Page: 1 of 1

Project Name: Default
Date Printed:
19.10.2017
17:58:23 Europe/Moscow

Figure S62. HPLC for (2*R*,3*S*)-**8e**.

Empower 2 software

chrom1

SAMPLE INFORMATION

Sample Name:		Acquired By:	System
Sample Type:	Unknown	Sample Set Name:	
Vial:	1	Acq. Method Set:	Hex_Pr_Meth_210
Injection #:	2	Processing Method	mnhhggfd
Injection Volume:	10,00 ul	Channel Name:	2487Channel 2
Run Time:	120,0 Minutes	Proc. Chnl. Descr.:	230nm
Date Acquired:	29.08.2017 13:51:02 MSD		
Date Processed:	29.08.2017 15:18:39 MSD		

Auto-Scaled Chromatogram

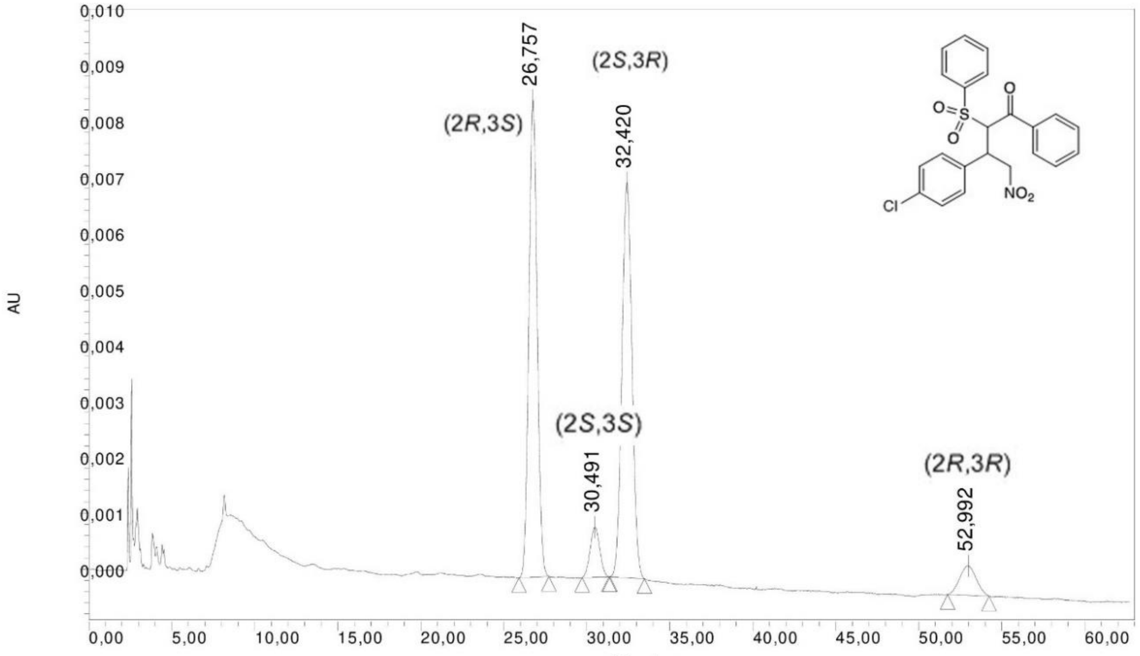

Peak Results

	RT	Height	Area	% Area
1	26,757	8517	296836	44,48
2	30,491	893	36113	5,41
3	32,420	7058	297564	44,59
4	52,992	527	36831	5,52

Reported by User: System
Report Method: chrom1
Report Method ID 10325
Page: 1 of 1

Project Name: Default
Date Printed:
29.08.2017
15:33:34 Europe/Moscow

Figure S63. HPLC for racemic **8f/9f** (a mixture of diastereomers).

chrom1

SAMPLE INFORMATION

Sample Name:		Acquired By:	System
Sample Type:	Unknown	Sample Set Name:	
Vial:	1	Acq. Method Set:	Hex_Pr_Meth_210
Injection #:	28	Processing Method	ioo
Injection Volume:	10,00 ul	Channel Name:	2487Channel 2
Run Time:	200,0 Minutes	Proc. Chnl. Descr.:	230nm
Date Acquired:	12.10.2017 11:52:45 MSD		
Date Processed:	12.10.2017 13:11:33 MSD		

Auto-Scaled Chromatogram

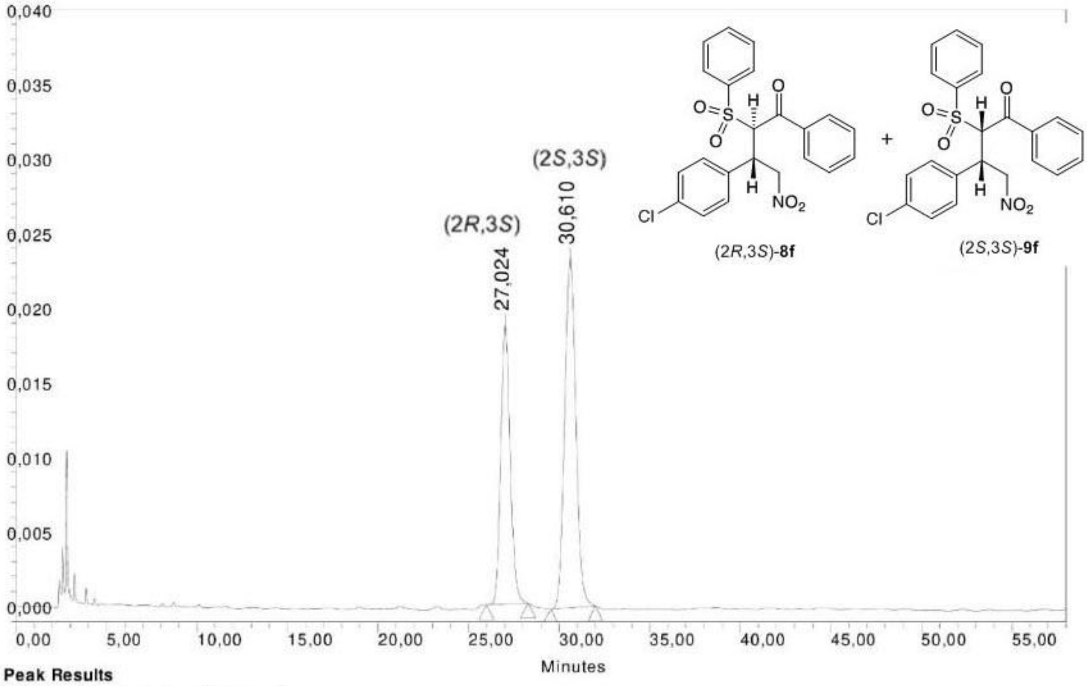

Peak Results

	RT	Height	Area	% Area
1	27,024	18678	684695	40,53
2	30,610	23375	1004744	59,47

Reported by User: System
Report Method: chrom1
Report Method ID 10577
Page: 1 of 1

Project Name: Default
Date Printed:
12.10.2017
13:14:42 Europe/Moscow

Figure S64. HPLC for (2*R*,3*S*)-**8f** and (2*S*,3*S*)-**9f**.

chrom2

SAMPLE INFORMATION

Sample Name:	alkene-Cl-o_(rac)AD_25%1.2ml	Acquired By:	System
Sample Type:	Unknown	Sample Set Name:	
Vial:	1	Acq. Method Set:	Hex_Pr_Meth_210
Injection #:	10	Processing Method	u
Injection Volume:	10,00 ul	Channel Name:	2487Channel 1
Run Time:	250,0 Minutes	Proc. Chnl. Descr.:	210nm
Date Acquired:	16.11.2017 15:23:55 MSK		
Date Processed:	16.11.2017 15:50:47 MSK		

Auto-Scaled Chromatogram

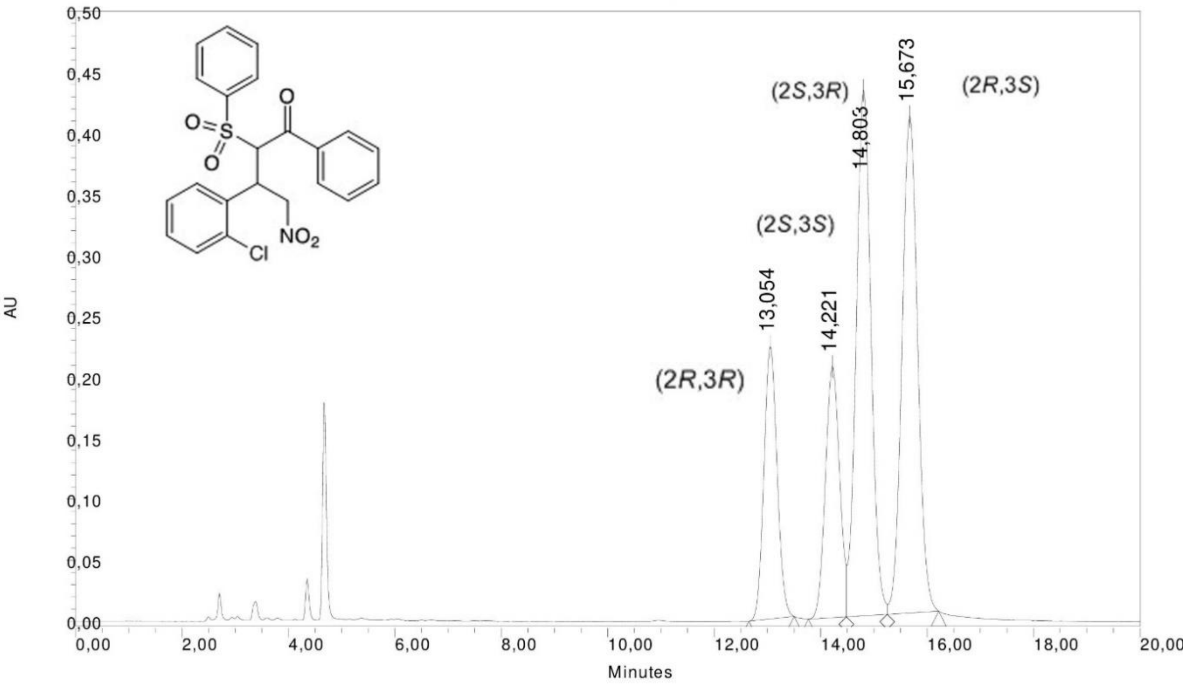

Peak Results

	RT	Height	Area	% Area
1	13,054	223444	3848618	15,79
2	14,221	206022	3821082	15,68
3	14,803	430906	8367081	34,34
4	15,673	407343	8331704	34,19

Reported by User: System
Report Method: chrom2
Report Method ID 10891
Page: 1 of 1

Project Name: Default
Date Printed:
16.11.2017
15:52:23 Europe/Moscow

Figure S65. HPLC for racemic **8g**/**9g** (a mixture of diastereomers).

Empower2 software

chrom2

SAMPLE INFORMATION

Sample Name:	alkene-Cl-o_(ind)AD_25%1.2ml	Acquired By:	System
Sample Type:	Unknown	Sample Set Name:	
Vial:	1	Acq. Method Set:	Hex_Pr_Meth_210
Injection #:	11	Processing Method	ioiop
Injection Volume:	10,00 ul	Channel Name:	2487Channel 1
Run Time:	250,0 Minutes	Proc. Chnl. Descr.:	210nm
Date Acquired:	16.11.2017 15:49:03 MSK		
Date Processed:	16.11.2017 16:13:24 MSK		

Auto-Scaled Chromatogram

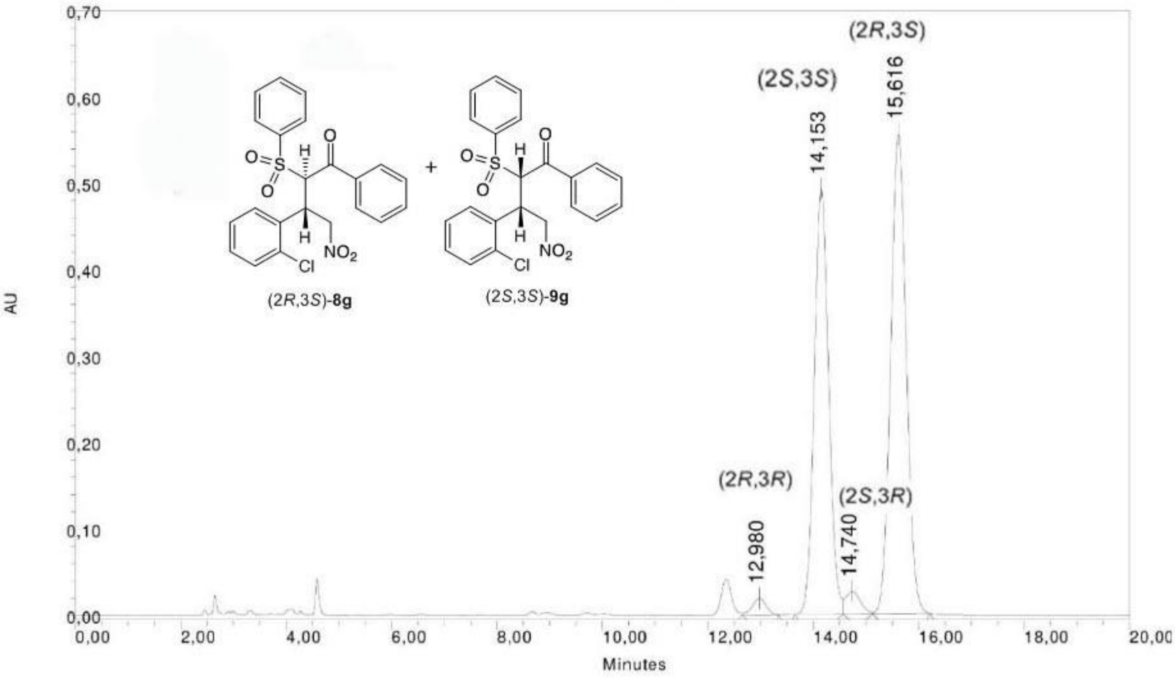

Peak Results

	RT	Height	Area	% Area
1	12,980	19245	352015	1,62
2	14,153	492312	9402789	43,41
3	14,740	25963	503002	2,32
4	15,616	555123	11404767	52,65

Reported by User: System
Report Method: chrom2
Report Method ID 10901
Page: 1 of 1

Project Name: Default
Date Printed:
16.11.2017
16:15:00 Europe/Moscow

Figure S66. HPLC for (2*R*,3*S*)-**8g** and (2*S*,3*S*)-**9g**.

Empower 2 software

chrom2

SAMPLE INFORMATION

Sample Name:		Acquired By:	System
Sample Type:	Unknown	Sample Set Name:	
Vial:	1	Acq. Method Set:	Hex_Pr_Meth
Injection #:	17	Processing Method	vhjghj
Injection Volume:	10,00 ul	Channel Name:	2487Channel 1
Run Time:	250,0 Minutes	Proc. Chnl. Descr.:	
Date Acquired:	17.11.2017 12:49:55 MSK		
Date Processed:	17.11.2017 16:34:39 MSK		

Auto-Scaled Chromatogram

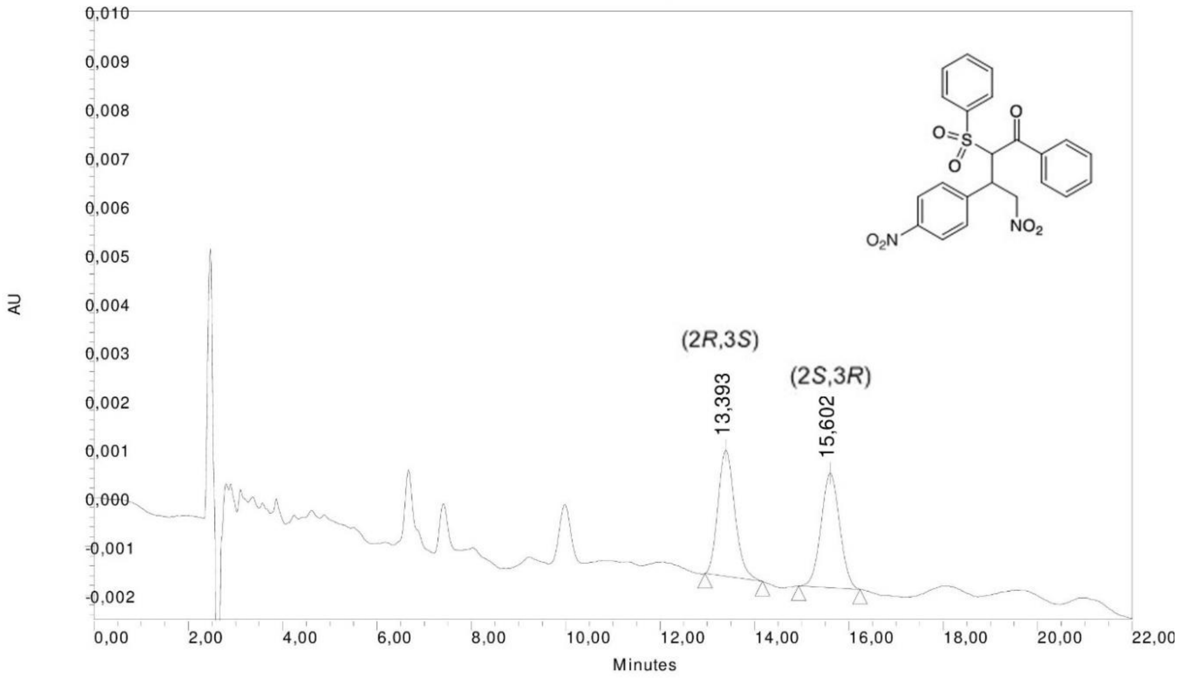

Peak Results

	RT	Height	Area	% Area
1	13,393	2589	64543	49,94
2	15,602	2356	64685	50,06

Reported by User: System
Report Method: chrom2
Report Method ID 10974
Page: 1 of 1

Project Name: Default
Date Printed:
17.11.2017
16:35:11 Europe/Moscow

Figure S67. HPLC for racemic **8h**.

Empower2 software

chrom2

SAMPLE INFORMATION

Sample Name:		Acquired By:	System
Sample Type:	Unknown	Sample Set Name:	
Vial:	1	Acq. Method Set:	Hex_Pr_Meth
Injection #:	18	Processing Method	nbnbv
Injection Volume:	10,00 ul	Channel Name:	2487Channel 1
Run Time:	250,0 Minutes	Proc. Chnl. Descr.:	
Date Acquired:	17.11.2017 13:47:45 MSK		
Date Processed:	17.11.2017 15:05:36 MSK		

Auto-Scaled Chromatogram

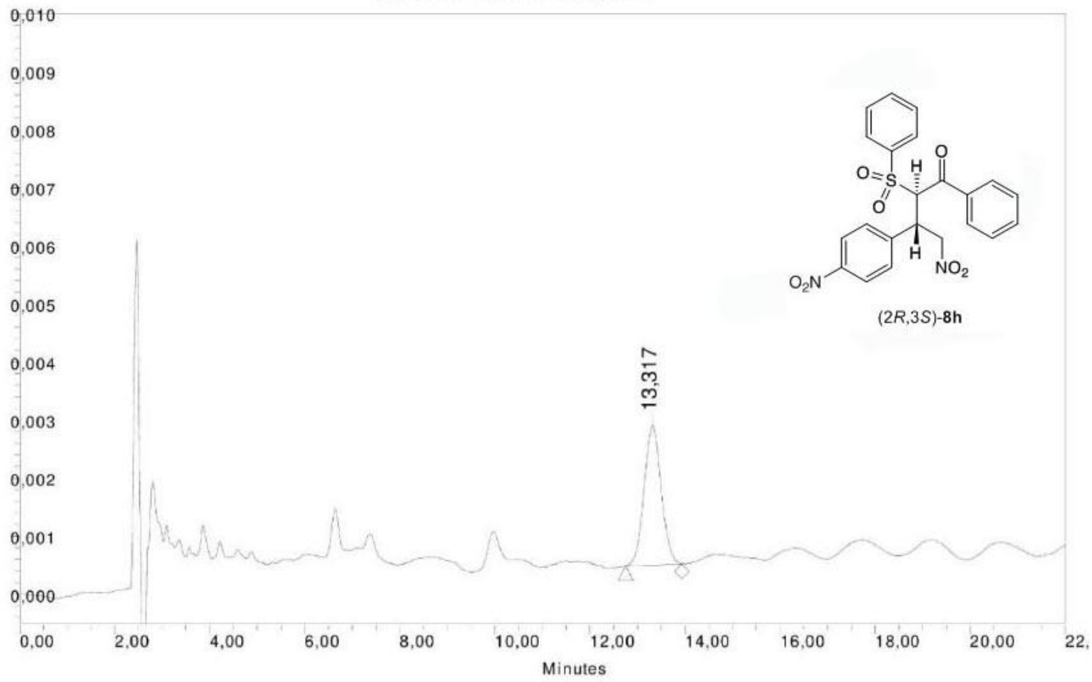

Peak Results

	RT	Height	Area	% Area
1	13,317	2405	60224	100,00

Reported by User: System
Report Method: chrom2
Report Method ID 10979
Page: 1 of 1

Project Name: Default
Date Printed:
17.11.2017
16:37:35 Europe/Moscow

Figure S68. HPLC for (2*R*,3*S*)-**8h**.

Empower 2 software

chrom1

SAMPLE INFORMATION

Sample Name:		Acquired By:	System
Sample Type:	Unknown	Sample Set Name:	
Vial:	1	Acq. Method Set:	Hex_Pr_Meth_210
Injection #:	3	Processing Method	ljhggf
Injection Volume:	10,00 ul	Channel Name:	2487Channel 1
Run Time:	250,0 Minutes	Proc. Chnl. Descr.:	210nm
Date Acquired:	15.11.2017 14:16:02 MSK		
Date Processed:	15.11.2017 15:08:50 MSK		

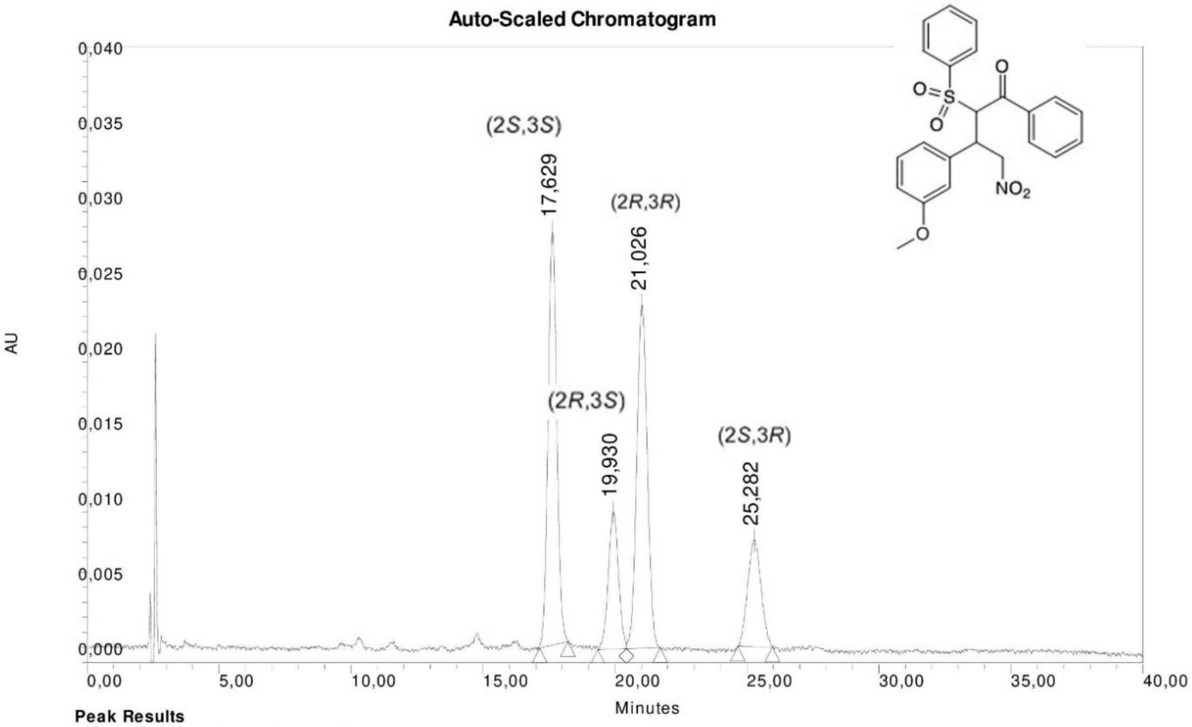

Peak Results

	RT	Height	Area	% Area
1	17,629	27513	635081	35,98
2	19,930	9152	246827	13,98
3	21,026	22884	634410	35,94
4	25,282	7082	248780	14,09

Reported by User: System
Report Method: chrom1
Report Method ID 10836
Page: 1 of 1

Project Name: Default
Date Printed:
15.11.2017
15:11:39 Europe/Moscow

Figure S69. HPLC for racemic **8i**/**9i** (a mixture of diastereomers).

Empower 2 software

chrom1

SAMPLE INFORMATION

Sample Name:		Acquired By:	System
Sample Type:	Unknown	Sample Set Name:	
Vial:	1	Acq. Method Set:	Hex_Pr_Meth_210
Injection #:	5	Processing Method	II
Injection Volume:	10,00 ul	Channel Name:	2487Channel 1
Run Time:	250,0 Minutes	Proc. Chnl. Descr.:	210nm
Date Acquired:	15.11.2017 15:40:36 MSK		
Date Processed:	15.11.2017 16:16:52 MSK		

Auto-Scaled Chromatogram

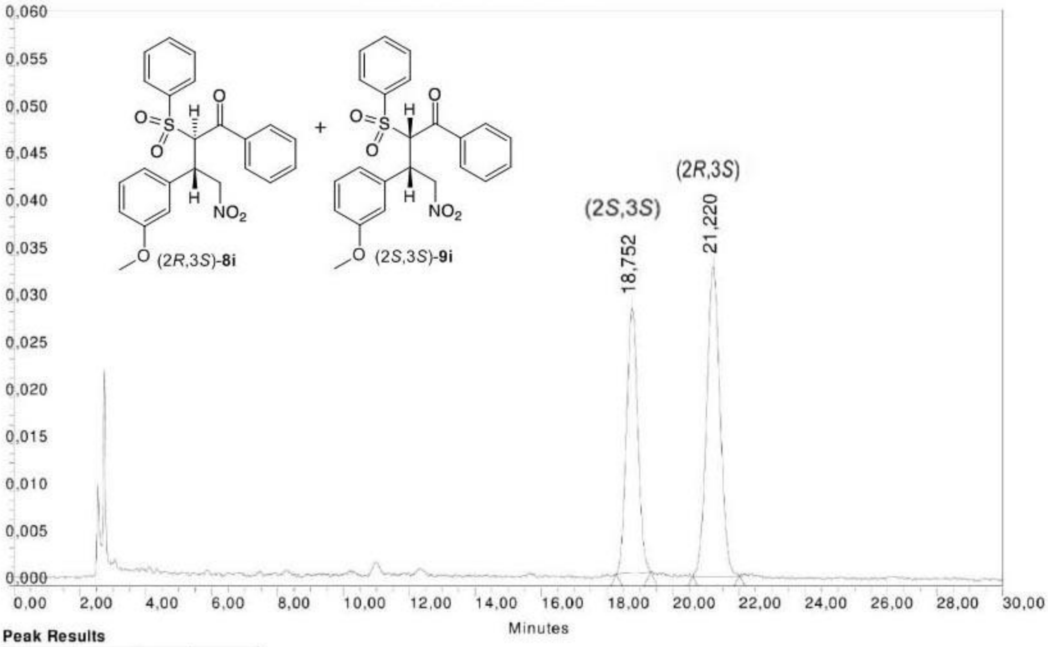

Peak Results

	RT	Height	Area	% Area
1	18,752	28081	671133	41,91
2	21,220	32709	930414	58,09

Reported by User: System
Report Method: chrom1
Report Method ID 10855
Page: 1 of 1

Project Name: Default
Date Printed:
15.11.2017
16:17:49 Europe/Moscow

Figure S70. HPLC for (2*R*,3*S*)-**8i** and (2*S*,3*S*)-**9i**.

X-ray diffraction data of compound 8d

X-ray data for (2*R*,3*S*)-1-(1-adamantyl)-4-nitro-3-phenyl-2-(phenylsulfonyl)-butane-1-on (8d). Crystals of ($C_{26}H_{29}NO_5S$, *M* = 467.56) are orthorhombic, space group $P2_12_12_1$, at 295 K: *a* = 10.0147(2), *b* = 14.0144(3), *c* = 17.4625(5) Å, α = 90.000°, β = 90.000°, γ = 90.000°, *V* = 2450.86(10) $Å^3$, *Z* = 4, $d_{exp.}$ = 1.267 g·cm^{-3}, μ(Cu*K*α) = 1.472 mm^{-1}, *F*(000) = 992. Intensities of 4874 reflections were measured with a Pilatus Stoe STADI VARI diffractometer [λ(Cu*K*α) = 1.54180 Å, ω-scans, θ < 73.127°], and 3537 independent reflections with R_{int} = 0.0497 (before absorption correction) were used in further refinement. Flack parameter 0.024(15). CCDC deposit 1590390 contains the supplementary crystallographic data for this paper. These data can be obtained free of charge from The Cambridge Crystallographic Data Centre *via* http://www.ccdc.cam.ac.uk.

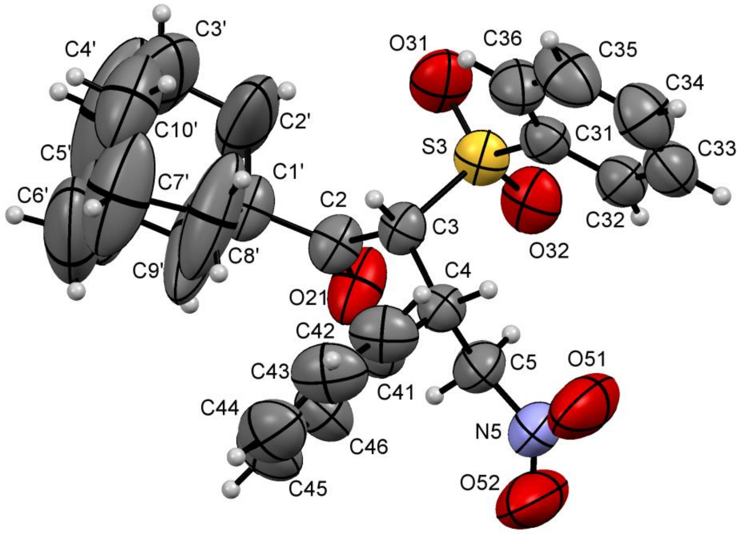

Figure S1. ORTEP diagram of (2*R*,3*S*)-**8d**.

Table S1. Selected bond lengths of (2*R*,3*S*)-**4d**.

№	Bond	Bond length (Å)		№	Bond	Bond length (Å)
**1**	C(2)-O(21)	1.216(4)		**20**	C(41)-C(46)	1.379(5)
**2**	C(2)-C(1)'	1.513(5)		**21**	C(41)-C(42)	1.390(5)
**3**	C(2)-C(3)	1.537(4)		**22**	C(42)-C(43)	1.387(7)
**4**	C(3)-C(4)	1.536(4)		**23**	C(43)-C(44)	1.376(8)
**5**	C(3)-S(3)	1.821(3)		**24**	C(44)-C(45)	1.373(7)
**6**	C(4)-C(41)	1.524(4)		**25**	C(45)-C(46)	1.378(6)
**7**	C(4)-C(5)	1.533(4)		**26**	C(1)'-C(8)'	1.464(7)
**8**	C(5)-N(5)	1.495(5)		**27**	C(1)'-C(2)'	1.468(9)
**9**	N(5)-O(51)	1.179(5)		**28**	C(1)'-C(9)'	1.472(7)
**10**	N(5)-O(52)	1.182(5)		**29**	C(2)'-C(3)'	1.562(11)
**11**	S(3)-O(31)	1.432(3)		**30**	C(3)'-C(10)'	1.457(15)
**12**	S(3)-O(32)	1.444(3)		**31**	C(3)'-C(4)'	1.47(2)
**13**	S(3)-C(31)	1.733(3)		**32**	C(4)'-C(5)'	1.43(2)
**14**	C(31)-C(36)	1.384(5)		**33**	C(5)'-C(6)'	1.426(17)
**15**	C(31)-C(32)	1.389(5)		**34**	C(5)'-C(9)'	1.560(11)
**16**	C(32)-C(33)	1.387(5)		**35**	C(6)'-C(7)'	1.480(19)
**17**	C(33)-C(34)	1.377(7)		**36**	C(7)'-C(10)'	1.449(16)
**18**	C(34)-C(35)	1.390(7)		**37**	C(7)'-C(8)'	1.533(10)
**19**	C(35)-C(36)	1.387(6)				

Table S2. Selected bond angles of (2*R*,3*S*)-**4d**.

№	Angle	(°)		№	Angle	(°)
**1**	O(21)-C(2)-C(1)'	121.7(3)		**29**	C(46)-C(41)-C(4)	123.3(3)
**2**	O(21)-C(2)-C(3)	116.8(3)		**30**	C(42)-C(41)-C(4)	117.7(3)
**3**	C(1)'-C(2)-C(3)	121.5(3)		**31**	C(43)-C(42)-C(41)	120.2(4)
**4**	C(4)-C(3)-C(2)	114.6(3)		**32**	C(44)-C(43)-C(42)	120.0(4)
**5**	C(4)-C(3)-S(3)	111.5(2)		**33**	C(45)-C(44)-C(43)	119.9(5)
**6**	C(2)-C(3)-S(3)	109.4(2)		**34**	C(44)-C(45)-C(46)	120.4(5)
**7**	C(41)-C(4)-C(5)	112.2(3)		**35**	C(45)-C(46)-C(41)	120.6(4)
**8**	C(41)-C(4)-C(3)	111.5(2)		**36**	C(8)'-C(1)'-C(2)'	103.3(7)
**9**	C(5)-C(4)-C(3)	113.7(3)		**37**	C(8)'-C(1)'-C(9)'	111.9(7)
**10**	N(5)-C(5)-C(4)	109.6(3)		**38**	C(2)'-C(1)'-C(9)'	105.2(8)
**11**	O(51)-N(5)-O(52)	123.0(4)		**39**	C(8)'-C(1)'-C(2)	113.8(3)
**12**	O(51)-N(5)-C(5)	119.3(3)		**40**	C(2)'-C(1)'-C(2)	112.0(4)
**13**	O(52)-N(5)-C(5)	117.5(4)		**41**	C(9)'-C(1)'-C(2)	110.1(4)
**14**	O(31)-S(3)-O(32)	118.17(16)		**42**	C(1)'-C(2)'-C(3)'	115.0(7)
**15**	O(31)-S(3)-C(31)	109.54(16)		**43**	C(10)'-C(3)'-C(4)'	109.8(10)
**16**	O(32)-S(3)-C(31)	107.58(16)		**44**	C(10)'-C(3)'-C(2)'	106.0(8)
**17**	O(31)-S(3)-C(3)	107.76(15)		**45**	C(4)'-C(3)'-C(2)'	99.3(11)
**18**	O(32)-S(3)-C(3)	109.80(15)		**46**	C(5)'-C(4)'-C(3)'	117.3(8)
**19**	C(31)-S(3)-C(3)	102.93(14)		**47**	C(6)'-C(5)'-C(4)'	111.8(11)
**20**	C(36)-C(31)-C(32)	121.3(3)		**48**	C(6)'-C(5)'-C(9)'	101.6(14)
**21**	C(36)-C(31)-S(3)	119.2(3)		**49**	C(4)'-C(5)'-C(9)'	107.0(11)
**22**	C(32)-C(31)-S(3)	119.5(3)		**50**	C(5)'-C(6)'-C(7)'	113.8(9)
**23**	C(33)-C(32)-C(31)	118.8(4)		**51**	C(10)'-C(7)'-C(6)'	112.7(8)
**24**	C(34)-C(33)-C(32)	120.5(4)		**52**	C(10)'-C(7)'-C(8)'	97.3(12)
**25**	C(33)-C(34)-C(35)	120.2(4)		**53**	C(6)'-C(7)'-C(8)'	111.7(9)
**26**	C(36)-C(35)-C(34)	120.0(4)		**54**	C(1)'-C(8)'-C(7)'	112.5(5)
**27**	C(31)-C(36)-C(35)	119.1(4)		**55**	C(1)'-C(9)'-C(5)'	113.4(6)
**28**	C(46)-C(41)-C(42)	119.0(4)		**56**	C(7)'-C(10)'-C(3)'	115.3(9)

References

1. Hamed, E.A.; El-Saadi, M. S. M.; El-Hegazy, F. M. *Synth. Commun.* **1995**, *25* (21), 3471–3478. doi: 10.1080/00397919508013871.

2. Furniss, B.; Hannaford, A.; Smith, P.; Tatchell, A. *Vogel's Textbook of Practical Organic Chemistry 5th Ed.*; Longman Science & Technical: London, 1989; p. 1035.

3. Evans, D. A.; Mito, S.; Seidel, D. *J. Am. Chem. Soc.* **2007**, *129*, 11583–11592. doi: 10.1021/ja0735913

4. Sibiryakova, A. E.; Reznikov, A. N.; Rybakov, V. B.; Klimochkin, Yu. N. *Russ. J. Gen. Chem.* **2016**, *86* (11), 2477–2483. doi: 10.1134/S107036321611013X.

5. Reznikov, A. N.; Kapranov, L. E.; Ivankina, V. V.; Sibiryakova, A. E.; Rybakov, V. B.; Klimochkin, Yu. N. *Helv. Chim. Acta* **2018**, *101*, e1800170. doi: 10.1002/hlca.201800170.
